# Cost–effectiveness analysis of prostate-specific antigen screening in China: a middle-income population-based microsimulation study

**DOI:** 10.1016/j.lanwpc.2025.101683

**Published:** 2025-09-16

**Authors:** Jiacheng Liu, Yuanshi Jiao, Yueting Huang, Yongle Zhan, Ruofan Shi, Xiaohao Ruan, Chi Yao, Ruochen Ma, Salida Ali, Tsun Tsun Chun, Da Huang, Danfeng Xu, Qian Zhang, Di Gu, Xue Li, Rong Na

**Affiliations:** a Department of Urology, Ruijin Hospital, Shanghai Jiao Tong University School of Medicine, Shanghai, China; b Department of Surgery, School of Clinical Medicine, LKS Faculty of Medicine, The University of Hong Kong, Hong Kong Special Administrative Region of China; c Department of Surgery, Queen Mary Hospital, Hong Kong Special Administrative Region of China; d Department of Medicine, School of Clinical Medicine, LKS Faculty of Medicine, The University of Hong Kong, Hong Kong Special Administrative Region of China; e Centre for Safe Medication Practice and Research, Department of Pharmacology and Pharmacy, LKS Faculty of Medicine, The University of Hong Kong, Hong Kong Special Administrative Region of China; f Laboratory of Data Discovery for Health (D^2^4H), Hong Kong Special Administrative Region of China; g Guangdong Provincial Key Laboratory of Urological Diseases, Department of Urology, The First Affiliated Hospital of Guangzhou Medical University, Guangzhou, China; h Guangdong Engineering Research Centre of Urinary Minimally Invasive Surgery Robot and Intelligent Equipment, Guangzhou Medical University, Guangzhou, China; i Guangzhou Institute of Urology, Guangzhou Medical University, Guangzhou, China; j Department of Urology, Capital Medical University Affiliated Beijing Shijitan Hospital, Beijing, China

**Keywords:** Prostate cancer, Prostate-specific antigen, Screening, Early detection, Risk assessment, Polygenic risk score, Cost-effectiveness analysis, Middle-income countries

## Abstract

**Background:**

The significance of prostate-specific antigen (PSA) in reducing the health burden of prostate cancer is widely deliberated. We conducted this study, utilising real-world data, to develop a comprehensive, cost-effectiveness analysis model for PSA screening. By evaluating various screening strategies, we aim to provide policymakers with robust research evidence to inform future PSA screening policies.

**Methods:**

We constructed a microsimulation model to assess 56 conventional population-wide PSA screening strategies based on data from a five-year trial with 104,751 participants, a 20-year multicentre database and parameters collected across China, along with approaches involving genetic risk stratification based on family history, polygenic risk scores, and high-penetrance genes. Clinical outcomes including incidence, metastasis-incidence ratio (M/I ratio), and annual case-fatality rate (CFR) were evaluated. Cost-effectiveness was evaluated using incremental quality-adjusted life years (QALYs) and incremental cost-effectiveness ratios (ICERs). Sensitivity and scenario analyses were conducted to test the robustness of the results.

**Findings:**

All strategies led to QALY gains and were considered cost-effective under a willingness-to-pay threshold equal to China's per capita GDP ($12,510.12 per QALY). The most intensive protocol (45–74 years, annually, with age-specific PSA cutoffs) had an ICER of 5535.25USD/QALY, yielding 2.79 incremental QALYs compared to non-screening, reducing M/I ratio from 39.05% to 1.04%, and CFR from 6.14% to 2.85%. The genetic risk-specific protocol offered comparable QALYs (2.79 vs. 2.76) and ICERs (5287.23 vs. 4904.90USD/QALY), allowing for 66.8% of average-risk individuals with extended screening intervals or postponed screening start age.

**Interpretation:**

PSA screening in China has the potential to improve overall health outcomes in a cost-effective manner. Personalised screening based on genetic risk may provide an efficient alternative to uniform strategies, potentially reducing unnecessary interventions among those at lower risk. This study provides a solid evidence base for Chinese policymakers to consider establishing a cost-effective, risk-stratified PCa screening programme.

**Funding:**

Shenzhen-Hong Kong-Macau S&T Programme, HKU Seed Fund, RJH Cultivating Star Programme, HK UGC Research Impact Fund and Guangdong High-Level Talents Programme.

**Research in context**

**Evidence before this study**

We searched PubMed for research articles published between database inception and 22 July 2024, using the search terms (“Prostate-Specific Antigen” [Mesh]) AND (“Mass Screening” [Mesh] OR “Early Detection of Cancer” [Mesh]) AND (“Cost–Effectiveness Analysis” [Mesh] OR “Cost-Benefit Analysis” [Mesh]). We subsequently reviewed 105 returned titles and abstracts. The overall cost-effectiveness of routine PSA screening remains controversial and highly sensitive to assumptions. Moreover, the majority of these findings are based on Western populations in developed countries. Only two studies evaluated PSA screening in Chinese populations, which are characterised by substantial regional disparities and notably different healthcare systems and costs compared to developed regions. Furthermore, these two studies typically evaluate only a single screening strategy although variations in screening age ranges and screening intervals may significantly influence economic outcomes, and do not model the dynamic changes in incidence in the population-ageing background, potentially limiting their robustness and reliability.

**Added value of this study**

This is the first cost-effectiveness study applying microsimulation model for projecting the incidence of prostate cancer in China. By evaluating 56 different screening strategies, we demonstrate that all the PSA screening strategies among Chinese males aged 45–74 years can improve overall QALYs while remaining cost-effective. Furthermore, tailoring PSA screening based on polygenic risk scores and family history may provide a viable alternative, enabling approximately two-thirds of the population to postpone the initial screening age without compromising clinical benefit.

**Implications of all the available evidence**

This study provides robust evidence of the cost-effectiveness of the PSA screening policy in China. We propose inclusion of polygenic risk score testing to reduce unnecessary PSA screening without compromising QALY gains, offering a precision-based alternative to streamline screening strategies among high-risk populations. Our findings indicate that the application of PSA screening strategies in China may also serve as a reference for other countries with similar disease burden and social economic conditions.

## Introduction

Prostate cancer (PCa) is one of the most prevalent cancers among males worldwide. In the United States, it is the cancer with the highest incidence and the second highest number of deaths in male population.^1^ In China, PCa ranked sixth in incidence rate among males in 2022.^2^ This discrepancy of incidence may be attributed to lower screening rates in Asian populations.^3^ Many prominent guidelines endorse a shared decision-making approach to prostate-specific antigen (PSA) screening, where the test is offered to high-risk population, or well-informed men who express a preference for it.^4^, ^5^, ^6^ Currently, PSA screening coverage varies significantly across countries. In Switzerland, the coverage rate of PSA screening is around 70%.^7^ In the United States, 32.1% of men aged 50 or older undergo routine PSA screening.^8^ China, as a middle-income economy, has yet to officially propose a national PSA screening programme. The majority of prostate cancer cases are incidentally detected during examinations for symptoms related to benign prostatic hyperplasia.^9^ Despite the relatively low incidence of PCa in China, patients are often diagnosed at an advanced stage. Metastatic PCa accounted for 40%–70% of newly diagnosed cases in China, whereas localised PCa comprised approximately 40%.^10^ Similarly, more than 50% of initially detected PCa patients in Mexico are at metastasis stage.^11^ Thus, the high mortality related to metastasis PCa imposes a substantial public health burden and an urgent need for PCa screening in China as well as other middle-income countries (MICs).

PSA screening has been shown to reduce disease-related mortality and extend life expectancy; however, concerns about overdiagnosis of non-aggressive diseases and unnecessary invasive biopsies have also been raised.^12^ The cost-effectiveness of PSA screening strategies remains a topic of debate. Previous cost-effectiveness analyses (CEAs) have examined PSA screening mainly in Western male populations such as the European Randomized Study of Screening for Prostate Cancer (ERSPC) trial. It showed that screening can be cost-effective when it is limited to two or three screenings between ages 55 and 59 years in Europe.^13^ However, screening beyond age 63 is less cost-effective due to the loss of quality-adjusted life years (QALYs) caused by overdiagnosis.^13^ To date, no evidence has been reported regarding the cost-effectiveness of PSA screening in China and other MICs.

Genetic risk of PCa could be another important factor in the screening decision. In our previous study, family history, rare pathogenic mutations (RPMs) in high-penetrance genes (HPGs), and single nucleotide polymorphism (SNP)-based polygenic risk scores (PRS) were found to be consistently associated in individuals with inherited-risk of PCa.^14^ In a latest large-scale RCT with 40,292 invited males, PRS demonstrated strong diagnostic performance for identifying clinically significant disease and reducing the percentage of overtreatment.^15^ Therefore, some guidelines now note that incorporating PRS may be considered when personalizing screening decisions.^4^, ^5^, ^6^ However, the cost-effectiveness of screening in high-risk populations is yet to be evaluated given the additional costs of genetic testing or sequencing.

In this study, we aim to comprehensively evaluate the efficacy and cost-effectiveness of PSA screening with multiple screening strategies in China. We constructed a dynamic Markov micro-simulation model, incorporating the anticipated population aging pattern in coming years. To illustrate the overall scenario of China, a country with extensive geographic diversity, regional disparities, and variations in clinical practice, the heterogenic socioeconomic factors from different regions in the country were also fully evaluated in the model. To minimise potential bias stemming from ethnic and treatment pattern variations, we calibrated the model based on local prospective screening trials, consulted experts from multiple regions across the nation, and utilised a local 20-year multicentre database to estimate transition probabilities. By evaluating various PSA screening strategies under different scenarios, our findings seek to provide policymakers with robust research evidence to inform future PSA screening policies in China, as well as in other MICs.

## Methods

### Model overview

**Fig. 1:**
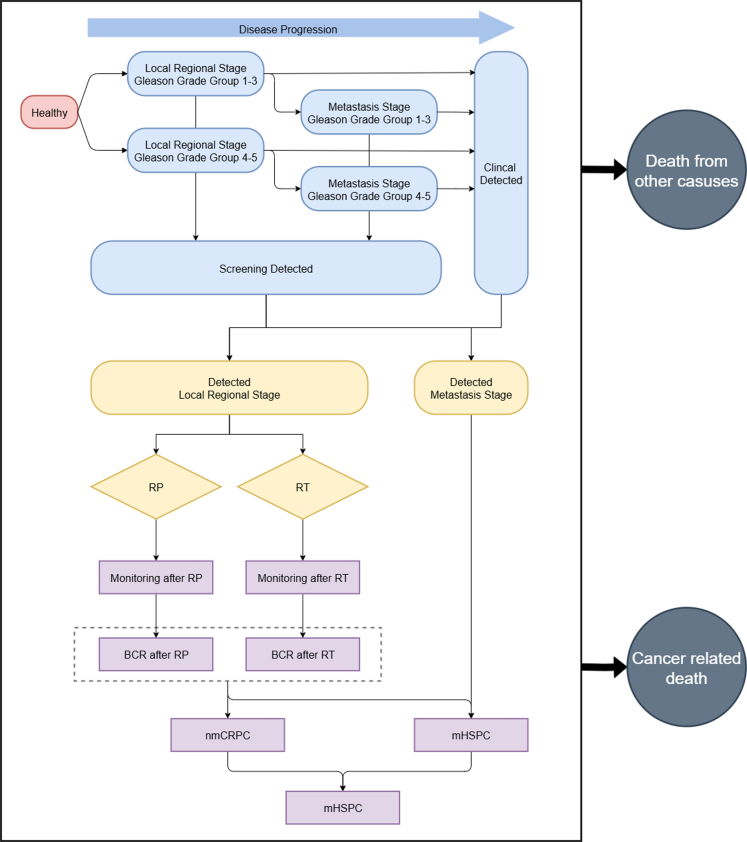
**Flowchart of the model**. The Figure illustrates the workflow of the research. The blue section represents the Fred Hutchinson Cancer Research Center Prostate Cancer Incidence Model, which simulates the epidemiology of PCa. The yellow section is the decision tree model, reflecting the treatment patterns of PCa in China. The purple section is the prognosis Markov model, which simulates disease progression and outcomes.

Model development and reporting were conducted according to the Consolidated Health Economic Evaluation Reporting Standards (CHEERS) reporting guideline.^16^ In this study, we constructed a comprehensive cohort simulation dynamic model to simulate cancer onset, progression, diagnosis, treatment, and prognosis in Chinese males (Fig. 1). This model is composed of three parts; (1) a Fred Hutchinson Cancer Research Centre microsimulation framework of prostate cancer^17^ based epidemiology model to project cancer incidence, (2) a decision tree model to simulate the PCa screening protocol and treatment pattern, and (3) an eight-state Markov cohort simulation model to mimic cancer prognosis with standard treatment. The incidence model operates on a yearly cycle length and the Markov model operates on a monthly cycle length. Only at the end of each 1-year cycle in the incidence model, the entire cohort of men newly diagnosed with cancer is evenly distributed across the 12 monthly entry cycles of the post-diagnosis Markov model. We have projected annual changes in the distribution of Chinese males aged 40 years and older by using a multi-age cohort and age-specific mortality rates of male in China 2020^18^ (Supplementary Figure S1. China Population Distribution Changing Trend). Our model naturally simulates a rising incidence of PCa as the population ages. The age distribution of Chinese males in 2020 (based on national census), 2030, 2040, and 2050 (estimated) are shown in Supplementary Figure S1. To be mentioned, new births were not incorporated, as they would not reach the screening start age (45 years) within the model's time horizon. Additionally, net migration, which only represents less than 5% of the crude death rate,^19^ was also not included into the model. Trends of PSA levels under different clinical scenarios are shown in Supplementary Figure S2. PSA Growth Trend, which are associated with age. With an increasingly ageing population in China, the incidence of PCa in this simulation increased gradually (Supplementary Figure S3a. Incidence rate among men aged 45–84). Detailed information of the model is described in Supplementary Methods. This study was approved by the Institutional Review Board of the University of Hong Kong/Hospital Authority Hong Kong West Cluster (IRB No. UW 22-766, UW 22-279) and of the First Affiliated Hospital of Guangzhou Medical University (IRB No. ES-2025-056-04). The study was registered at Chinese Clinical Trial Registry (No. ChiCTR2500104154). We confirm that all necessary consents required by applicable law from any relevant patient, research participant and/or other individual whose information is included in the article have been obtained in writing.

### Clinical input parameters

Parameters input into the model are described in Table 1.

**Table 1:** Parameters in the model.

Variables	Value	Range	Reference
**Parameters in cancer incidence model**^a^			
γ_o_ (hazard of tumour onset)	0.00024	0.00018–0.00032^b^	^20^
γ_m_ (hazard of tumour metastasis)	0.0004	0.0003–0.00052^b^	^21^
γ_c_ (hazard for a tumour being clinically detected)	0.0005	0.00038–0.00067^b^	^20^
γ_LR_ (proportion of low–risk cancers)	0.673	0.505–0.897^b^	e
θ_c_ (multiplier for clinical detection hazard after metastasis)	19.1334	14.3500–25.5112^b^	^21^
a_0_ (PSA growth slope in the healthy individuals)	0.0215	0.0210–0.0220^c^	e
a_1_ (PSA growth slope in low–risk tumours)	0.0566	0.0430–0.0693^c^	e
a_2_ (PSA growth slope in high–risk tumours)	0.1051	0.0870–0.1247^c^	e
b (PSA growth intercept)	−0.1061	−0.1670 to −0.046^c^	e
MRI sensitivity	0.89	0.83–0.93^c^	^22^
MRI specificity	0.80	0.62–0.90^c^	^22^
Biopsy sensitivity	0.64	0.48–0.80^d^	^23^
Biopsy specificity	1.00	0.90–1.00^c^	^24^
**Transition probability (per month)**			
From RP to BCR_RP	0.011431	0.010574–0.012297^c^	e
From RP to mHSPC	0.002305	0.001927–0.002685^c^	e
From RP to Death	0.000434	0.000270–0.000598^c^	e
From RT to BCR_RT	0.037776	0.034893–0.040758^c^	e
From RT to mHSPC	0.008584	0.007293–0.009895^c^	e
From RT to Death	0.070772	0.066492–0.075280^c^	e
From BCR_RP to nmCRPC	0.000045	0.000000–0.000132^c^	e
From BCR_RP to mHSPC	0.001437	0.000941–0.001936^c^	e
From BCR_RP to Death	0.000761	0.000400–0.001123^c^	e
From BCR_RT to nmCRPC	0.000904	0.000315–0.001496^c^	e
From BCR_RT to mHSPC	0.005351	0.003911–0.006813^c^	e
From BCR_RT to Death	0.053892	0.048843–0.059255^c^	e
From mHSPC to MCRPC	0.000657	0.000501–0.000814^c^	e
From mHSPC to Death	0.018824	0.017956–0.019700^c^	e
From nmCRPC to MCRPC	0.001153	0.000503–0.001807^c^	e
From nmCRPC to Death	0.040735	0.036570–0.045109^c^	e
From MCRPC to Death	0.045317	0.040162–0.050797^c^	e
**Costs (USD)**			
PSA	3.78	2.84–4.73	e
MRI	148.05	111.04–185.06	e
Prostate Biopsy	161.70	121.28–202.13	e
RP	6594.70	4946.03–8243.38	e
RT	4550.00	3412.50–5687.50	e
Post–RP/RT ADT (per month)	312.23	234.18–390.29	e
Post–RP/RT Monitoring (per month)	3.78	2.84–4.73	e
Post–RP/RT BCR ADT (per month)	297.81	223.36–372.27	e
mHSPC Treatment	1060.98	795.74–1326.23	e
nmCRPC Treatment	1004.09	753.06–1255.11	e
mCRPC Treatment	2054.00	1540.50–2567.50	e
WGS	252.00	189.00–315.00	
PRS	16.8	12.60–21.00	
**Utility**			
Post RP	0.800	0.600–1.000^d^	^25^
Post RT	0.800	0.600–1.000^d^	^25^
BCR Post RP	0.790	0.593–0.988^d^	^25^
BCR Post RT	0.790	0.593–0.988^d^	^25^
mHSPC	0.755	0.566–0.944^d^	^25^
nmCRPC	0.775	0.581–0.969^d^	Estimated
mCRPC	0.726	0.545–0.908^d^	^25^

RP, radical prostatectomy; RT, radiotherapy; BCR, biochemical recurrence; mHSPC, metastatic hormone–sensitive prostate cancer; nmCRPC, nonmetastatic castration–resistant prostate cancer; mCRPC, metastatic castration–resistant prostate cancer; WGS, whole-genome sequencing; PRS, polygenic risk score.

a The Fred Hutchinson Cancer Research Centre Prostate Cancer Incidence Model is fundamentally based on these assumptions: (a) The logarithm of PSA (denoted as P) growth is linearly correlated with age and the state of disease $P\left(t\right)=b+a_{0}t+a_{x}t\left(t-t_{o}\right)I\left(t>t_{o}i\right)+\varepsilon$, I(.) is an indicator function, $t_{o}$ denotes the age at onset of a preclinical tumour, and $a_{x}$ takes the value $a_{1}$ or $a_{2}$ depending on the Gleason Grade of the disease. (b) Disease progression is driven by age or PSA growth. Specifically, a hazard of disease onset is proportional to age $\lambda_{o}\left(t\right)=\gamma_{o}t$, while hazards of metastasis $\lambda_{m}\left(t\right)=\exp\left(P\right)\gamma_{m}$ and clinical detection $\lambda_{c}\left(t\right)=\exp\left(P\right)\gamma_{c}$ The hazard of clinical detection increases once the tumour metastasises: $\lambda_{c}\left(t\right)=\exp\left(P\right)\gamma_{c}\theta_{c}$. $\gamma_{LR}$ is the proportion of patients with tumour Gleason Grade Group ≤3.

b Indicates parameters varied from 0.75 to 1.33 times the baseline value.

c Indicates that the given range is a 95% confidence interval.

d Indicates parameters varied from 0.75 to 1.25 times the baseline value.

e Indicates that the parameter is derived from local data.

The PCa incidence in China is much lower than that in the Western countries^2^ and controversial remains whether the discrepancy is due to the differences of cancer management or the inherent factors.^3^ Thus, we calibrated the PSA growth based on the data from a Chinese single-tertiary-centre 5-year prospective screening trial involving 104,751 participants by Markov Chain Monte Carlo (MCMC) method. Tumour onset risk was assumed to be linearly correlated with age.^21^ For the onset rate and clinical diagnosis parameter, we performed a systematic parameter search, began with initial estimates, using a range of factors (from 1.0 to 4.0, in strides of 0.2) to multiply or to divide the initial parameters, until the lowest Mean Absolute Error (MAE) of age-specific incidence compared with WHO reported results^20^ was reached (Supplementary Table S2. Validation of the Fred Hutchinson Cancer Research Centre Prostate Cancer Incidence Model against WHO Estimates in China). We conservatively estimated cancer incidence, in order to project smaller benefits from screening while relatively magnifying its costs and extra management processes. This provides a buffer to our conclusions and enhances the robustness of our study. We assumed disease grade (low-risk defined as Gleason grade group ≤3 or high-risk defined as Gleason grade group ≥4) is determined at onset and does not change over time.^17^^,^^26^ The proportion was calculated based on data from the five-year prospective screening trial. Transition probabilities (including survival) between eight states in the Markov model were generated from retrospective analysis of territory-wide electronic medical records (EMR) administered by the Hong Kong Hospital Authority (HA). The HA oversees all public hospitals, specialist, and general outpatient clinics in Hong Kong, and is responsible for approximately 80% of all hospital admissions within the region. This data consists of male patients diagnosed with PCa, identified by the ICD-9 code 185, between 1 January 2001 and 31 December 2021, offering a 20-year window to observe disease trends in Chinese. All the patients had been followed up for over 1 year. The castration-resistance of PCa patients was identified by the follow-up PSA rise.^27^ The monthly transition probabilities for the model were calculated directly from this empirical real-world database by identifying patients' disease states and tracking the observed transitions between them.

### Cost related to screening and treatment

Proportion of treatment, all the costs of tests, examinations and interventions (PSA test, MRI, biopsy, radical prostatectomy (RP) and radiotherapy) in this study (Table 1) were derived from a survey of 28 urology experts from districts across the country, compared with and adjusted according to the *National Technical Specifications for Medical Service Projects*^28^ and *Medical Service Price Catalogue of Each Province*.^29^ Drug costs were also determined based on the survey and were cross verified with publicly available information. The costs for each state per cycle in the Markov model were calculated using the recommended dosage. All costs are reported in USD (exchange rate of **1 CNY = 0.14 USD** in 2023).

### Utility, perspective, cycling length and discounting rate

Utilities were EQ-5D scores obtained from a previous 8 Asian countries/regions population-based multicentre study.^25^

Cycle length incidence model was set to 1 year, and that of prognosis Markov model was set to one month, aligning with the standard screening and monitoring schedule for PCa. The study adopted a time horizon of 30 years to emulate life-long effects. Both utility values and costs were discounted at an annual rate of 3%.

Detailed information of input parameters is described in Supplementary Methods.

### Screening Strategies

Based on recommendations from the 2022 Chinese PCa screening guidelines,^30^ we fixed the screening ending age at 74 years, with varying starting ages from 45 to 60 years and intervals with 1, 2, 3, and 5 years. Previous studies suggest that increasing the PSA threshold among elderly populations can effectively reduce overdiagnoses.^26^ Thus, we incorporated age-specific PSA thresholds (2.0 ng/mL, 3.0 ng/mL, 4.0 ng/mL, and 7.0 ng/mL for individuals aged <50 years, 50–59 years, 60–69 years, and ≥70 years, respectively)^31^ into our model and evaluated its effectiveness. In strategies without age-specific PSA thresholds, we fixed the test-positive threshold to be 4 ng/mL. Individual with PSA over threshold will experience a following mpMRI examination. PI-RADS ≥4 is regarded as the indicator for a subsequent cognitive MRI-targeted biopsy. Individual with PSA >10 ng/mL will directly undergo a systematic biopsy procedure. Sensitivity of the biopsy was defined as 64%^23^ (Supplementary Methods).

Additionally, we categorised the population into high- and average-risk groups based on family history, PRS and RPMs. We simulated the application of either a one-time WGS test or a PRS test for all males to distinguish high- and average-risk individuals. The proportion of high- and average-risk population was based on our previous study.^14^ Patients with family history, or top 25% of PRS, or carrying rare pathogenic mutations are identified as high-risk populations (Supplementary Table S1). For high-risk men, we modelled annual screening beginning at age 45. For average-risk men, we assessed a range of scenarios including no screening, screening starting ages of 50, 55, and 60, and screening intervals of one and two years. We assumed 100% adherence to screening visits, MRI, biopsy and treatment.

### Statistical analysis

Clinical outcomes including annual incidence rate, the metastasis rate at initial detection, metastasis-to-incidence ratio (M/I ratio) and annual case-fatality rate (CFR) were calculated and compared. Incremental costs, incremental QALYs, and incremental cost-effectiveness ratios (ICERs) were calculated by comparing screening strategies to the current non-screening status quo. The ICERs were then evaluated against a willingness-to-pay (WTP) threshold of 1 time the GDP per capita in China for 2023 (USD 12,510.12 per QALY gained), as recommended by the World Health Organization and previous studies.^32^^,^^33^ To provide a nationwide perspective, we also analysed the cost-effectiveness across a range of WTP thresholds. These thresholds were derived from the 2023 GDP per capita of economically diverse provinces and municipalities, representing varying income levels across China. The regions included Gansu ($6738.06), Hunan ($10,602.35), Guangdong ($15,007.15), Zhejiang ($17,493.90), Jiangsu ($21,081.75), and Shanghai ($26,699.95).

All analysis was conducted with Python (version: 3.11.7 | packaged by Anaconda, Inc.), and cross-validated with R version 4.4.2 (R Project for Statistical Computing). Model construction and input parameter generation were cross-checked independently by two coauthors (JC.L., YS.J.) for quality control.

### Sensitivity analyses

One-way sensitivity analysis was conducted by varying each parameter within its corresponding range independently. In probabilistic sensitivity analysis, all tested parameters were varied simultaneously with a predefined distribution for 1000 iterations. For parameters where standard deviations (SDs) were not available, we applied a range of 0.75–1.25 for linear parameters and a range of 0.75–1.33 for exponential parameters in the simulation referred to a previous study.^34^ The cost-effectiveness acceptability curve (CEAC) displayed the probability of each comparator being a cost-effective strategy across WTP threshold range USD 0 to USD 20,000.

### Scenario analysis

Our base–case parameter for the proportion of men with a positive family history was based on UKB databases^14^ (Supplementary Table S1). In China, the reported rate may be lower, partly reflecting China's under-diagnosis or under-reporting of familial cases.^35^ To account for this potential discrepancy and to test the robustness of our model to variations in the high-risk group classification, we performed a scenario analysis assuming a zero prevalence of family history. The outcomes of this conservative assumption are presented in Supplementary Table S5. Robustness Analysis: The Influence of High-Risk Group Proportion on Cost-Effectiveness Results, allowing assessment of the impact of potential underestimation in this subgroup.

### Role of the funding source

None of the funders had any role in study design, data collection, data analysis, interpretation, decision to publish, or preparation of the manuscript.

## Results

### Impact of screening on the PCa outcomes

According to our simulation (Supplementary Figure S1), the area under curve among male >60 years old is increasing along with the time. This indicates aging is an ongoing phenomenon in China. Over a 30-year perspective without screening, the average incidence of prostate cancer among Chinese men aged 45–84 years will be 99.46 cases per 100,000 population. At diagnosis, 39.05% of patients already have metastatic disease, resulting in an annual CFR of 6.14% (Supplementary Table S4. Full Incremental Cost-Effectiveness Results for All Simulated Prostate Cancer Screening Strategies). Nationwide universal PSA screening would initially lead to a surge in PCa incidence in the first few years (Supplementary Figure S3a and b), followed by a stabilisation in subsequent years. Despite the overall increase of incidence, the newly diagnosed metastasis and the M/I ratio decreased under this universal screening scenario (Supplementary Figure S3c and d).

We then simulated and evaluated the efficacy of screening using different screening strategies. Compared with non-screening, the least costly screening strategy (fixed PSA cutoff = 4.0 ng/mL; age 60–74 years; screening interval of 5 years) substantially reduces the number of metastatic cases at initial detection (from 38.84 to 19.20 per 100,000 population), the M/I ratio (from 39.05% to 4.46%), and improves survival outcomes (CFR decreases from 6.14% to 3.16%). Generally, compared with no screening, routine PSA screening is projected to reduce the 30-year average incidence of metastatic disease at diagnosis from 38.84 to 9.18–20.02 cases per 100,000 population and to lower the annual case-fatality rate from 6.14% to 2.86–3.21%.

### Base-case analysis

**Fig. 2:**
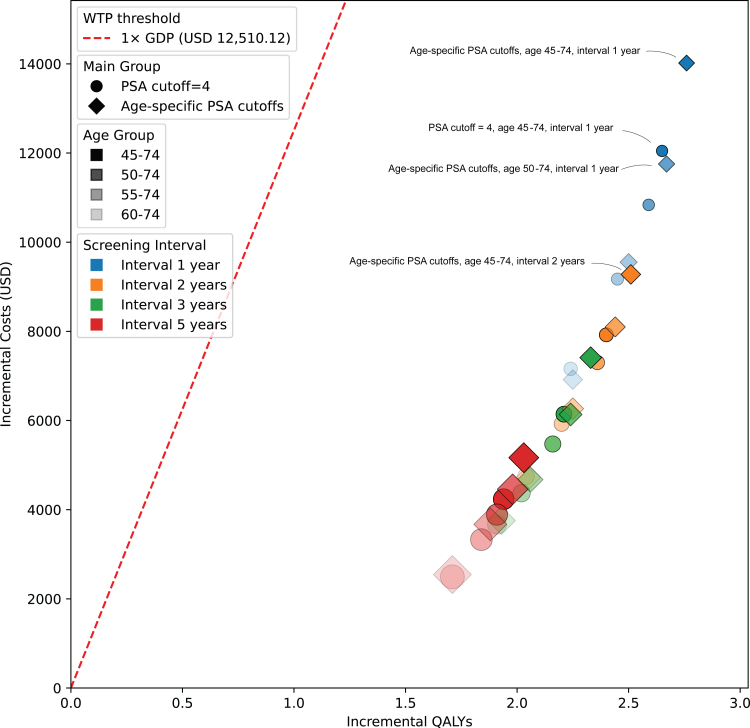
**Cost–effectiveness plane of PSA screening strategies**. This figure illustrates the incremental cost (USD) vs. incremental QALYs (Quality-Adjusted Life Years) gained for various prostate-specific antigen (PSA) screening strategies, each compared to a no-screening baseline. Each point represents a distinct combination of screening interval (color) and screening age group (color shade), with the PSA cutoff approach indicated by the marker shape (circles for a standard PSA cutoff of 4, diamonds for age-specific cutoffs). Age-specific PSA thresholds are defined as 2.0 ng/mL, 3.0 ng/mL, 4.0 ng/mL, and 7.0 ng/mL for individuals aged <50 years, 50–59 years, 60–69 years, and ≥70 years, respectively. The dashed red line marks the willingness-to-pay (WTP) threshold at 1 × GDP per capita (USD 12,510.12), guiding interpretation of cost-effectiveness. Points lying below and to the right of this line are generally more likely to be considered cost-effective, given the specified WTP. The size of each point is correlated to the annual death rate of prostate cancer in 30-year horizon. A larger size indicates a higher death rate.

Fig. 2 illustrates the incremental costs vs. incremental QALYs gained for various PSA screening strategies, compared to a non-screening baseline. Results showed that any screening strategy may have significant high cost-effectiveness, of which the incremental cost will be beneath the WTP threshold at 1 × GDP per capita (12,510.12 USD/QALY).

Additionally, strategies applying a fixed PSA cutoff of 4.0 ng/mL detect slightly more PCa cases than strategies using age-specific cutoffs and identify slightly more metastatic cases at initial detection (e.g., fixed PSA cutoff = 4.0 vs. age-specific PSA cutoffs, 45–74 years, annually; incidence 857.94 vs. 819.47 per 100,000; metastasis 9.18 vs. 9.26 per 100,000). However, there is no significant survival difference between these two strategies (CFR: 2.86% vs. 2.86%).

PSA screening is beneficial for increasing QALYs across all scenarios. Every proposed strategy met the criteria for cost-effectiveness, based on a predefined WTP threshold equal to China's per capita GDP. An earlier initiation of screening and a frequent screening strategy would lead to a better QALY gain but higher costs. For instance, when using age-specific PSA thresholds, annual screening beginning at age 45 yielded higher QALYs compared to starting at age 50 (2.76 vs. 2.67) but also resulted in a higher ICER ($5083.31 vs. $4408.12 per QALY). In contrast, a biannual screening strategy starting at age 45 produced a lower QALY gain (2.51) but had a substantially reduced ICER ($3693.24 per QALY). The most intensive strategy—annual screening from ages 45 to 74 using age-adjusted PSA cutoffs—achieved an ICER of $5083.31 per QALY, which represents approximately 42% of the WTP threshold, reaffirming its cost-effectiveness.

Furthermore, age-specific PSA cutoffs demonstrate greater efficiency in prolonged QALYs compared to the fixed 4.0 ng/mL cutoff. Under the same screening age and interval, applying age-specific PSA thresholds results in higher QALY gains (e.g., 2.76 vs. 2.65 for age 45–74 years with annual screening).

### Sensitivity and scenario analysis

In the one-way sensitivity analysis (Supplementary Figure S4. One-way Sensitivity Analysis), all corresponding ICERs remained constantly below the WTP threshold. Even the costliest protocol—annual screening from ages 45 to 74 using age-specific PSA cutoffs—remained cost-effective, with ICERs ranging from $4506.69 to $6331.21 per QALY, well within the threshold defined by China's GDP per capita. Furthermore, the probabilistic sensitivity analysis confirmed the stability of these findings. All screening strategies were found to be cost-effective under the WTP threshold in this analysis as well (Supplementary Figure S5. Probabilistic-Sensitivity Analysis).

**Fig. 3:**
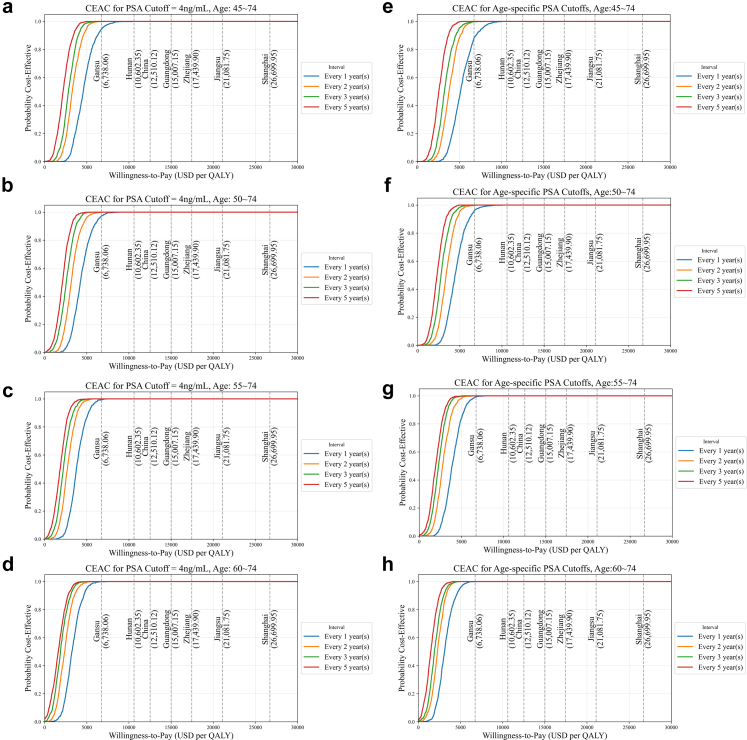
**Cost–effectiveness acceptability curves (CEACs) for different PSA cutoff thresholds and screening intervals**. This figure displays the Cost–effectiveness acceptability curves (CEACs) for various PSA cutoff thresholds (4 and age-specific) and screening intervals (every 1, 2, 3, and 5 years) across different age groups. Panels (a–d) show the CEACs for the screening age groups 45–74, 50–74, 55–74, and 60–74, respectively, under the PSA cutoff threshold of 4, while panels (e–h) represent the same age groups for age-specific PSA cutoff thresholds. The x-axis represents the willingness-to-pay (WTP) in USD per QALY, and the y-axis shows the probability of cost-effectiveness. The curves demonstrate the likelihood that each screening strategy is cost-effective at different levels of WTP, with different colors indicating the screening intervals. The data points on each curve represent the probability of cost-effectiveness at various WTP thresholds.

Fig. 3 indicated the CEACs for different WTP thresholds. With the exception of Gansu Province in Northwestern China—one of the country's least economically developed regions—all other analysed areas achieved 100% cost-effectiveness across all PSA screening strategies. In Gansu, while full cost-effectiveness was not observed across every scenario, the probability that PSA screening is cost-effective remained high, exceeding 88.0%. Notably, adjusting the screening approach—either by delaying the initiation age to 55–74 or 60–74 years, or by extending the screening interval to two years or more—resulted in 100% cost-effectiveness even in Gansu.

### Inherited-risk- specific screening strategies

**Fig. 4:**
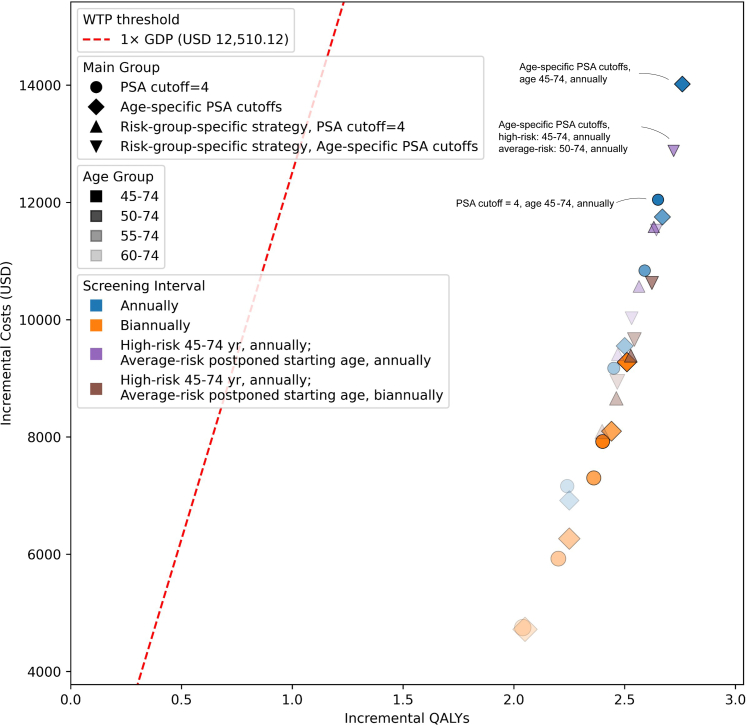
**Cost–effectiveness plane of PSA screening strategies (Considering Group-Risk-Specific Strategies)**. This figure illustrates the incremental cost (USD) vs. incremental QALYs (Quality-Adjusted Life Years) gained for various prostate-specific antigen (PSA) screening strategies, each compared to a no-screening baseline. Each point represents a distinct combination of screening interval (color) and screening age group (color shade), with the PSA cutoff approach indicated by the marker shape (circles: PSA cutoff = 4, diamonds: age-specific PSA cutoffs, upward triangles: risk-group-specific strategy with PSA cutoff = 4, downward triangles: risk-group-specific strategy with age-specific PSA cutoffs). Age-specific PSA thresholds are defined as 2.0 ng/mL, 3.0 ng/mL, 4.0 ng/mL, and 7.0 ng/mL for individuals aged <50 years, 50–59 years, 60–69 years, and ≥70 years, respectively. The dashed red line marks the willingness-to-pay (WTP) threshold at 1 × GDP per capita (USD 12,510.12), guiding interpretation of cost-effectiveness. Points lying below and to the right of this line are generally more likely to be considered cost-effective, given the specified WTP. The size of each point is correlated to the annual death rate of prostate cancer in 30-year horizon. A larger size indicates a higher death rate.

A one-time targeted genotyping for PRS provides individuals with comparable QALYs and ICERs while enabling two-thirds of average-risk males to postpone their screening starting age or increase their screening interval (Fig. 4). Specifically, a strategy involving annual screening for high-risk individuals aged 45–74, and for average-risk individuals aged 50–74, using age-specific PSA thresholds, resulted in 2.72 of incremental QALYs, 4743.38 of ICER, 1.21% of M/I ratio and 2.87% CFR. These results suggest that this risk-stratified approach may serve as a viable alternative to universal annual screening for all men aged 45–74, which yielded slightly higher QALYs (2.76) but also a higher ICER ($5083.31), with an M/I ratio of 1.13% and a CFR of 2.86%.

A one-time WGS did not improve cost-effectiveness compared to universal whole-population PSA screening for PCa diagnosis, as it yielded similar QALYs but higher ICERs compared to universal screening (e.g., using age-specific cutoffs, high-risk individuals aged 45–74 annually and average-risk group 50–74 annually vs. entire population aged 45–74 annually, ICER 5880.06 vs. 5083.31 USD, incremental QALYs 2.72 vs. 2.76, respectively; Supplementary Table S4). However, the ICER is still under the WTP threshold (12,510.12 USD).

## Discussion

To our knowledge, this is the first cost-effectiveness study leveraging the Fred Hutchinson Cancer Research Centre microsimulation model^17^ and real-world data from China to project the incidence of prostate cancer in a middle-income population while comparing various screening strategies based on age period, screening interval and the incorporation of genetic testing.

The present study has several important findings. Firstly, our results showed that PSA screening can significantly improve survival outcomes for patients with PCa in China while remaining highly cost-effective. On one hand, in China, a high proportion of men (historically 40%–70%) present with metastatic disease at their initial diagnosis,^10^ and this leads to a poor prognosis. By earlier detection before metastasis occurs, the model simulates a direct reduction in the incidence of incurable disease, which drives the projected reduction in prostate cancer mortality. This survival benefit is aligned with the outcome in previous studies.^36^^,^^37^ On the other hand, ICERs projected in all tested scenarios are within the pre-defined WTP threshold. In the context of cancer-related health interventions, patients are generally considered willing to pay up to three times the GDP per capita per additional QALY.^33^ According to our results, even in the least economically developed region of China, universal annual screening showed a probability higher than 88.0% of being cost-effective under the conservative threshold of one-time the GDP per capita. Performing the most conservative strategy (screening from 60 to 74 years, every 5 years) is projected to gain 1.71 QALYs while cost only an ICER of $1458.85–$1491.49 per QALY, one-fifth GPD per capita of the least economically develop region (Gansu $6738.06) in China. This suggests that such a programme is economically sustainable on a national scale. While our study provides a solid evidence base supporting the value of screening, a thorough assessment of the current healthcare infrastructure and workforce will be needed before widespread implementation. Secondly, our findings suggest that employing age-specific PSA thresholds, as opposed to a fixed cutoff of 4.0 ng/mL across all age groups, results in fewer prostate cancer diagnoses but greater gains in QALYs. This may be attributed to the ability of age-specific cutoffs to reduce the risk of overdiagnosis and subsequent overtreatment, thereby minimising long-term harms associated with treatment-related complications. Thirdly, genetic testing is emerging as a promising tool for identifying individuals at elevated risk of prostate cancer.^14^^,^^15^^,^^35^ In this study, we found applying family history and PRS test to stratify high- and average-risk populations proved to be a more cost-effective approach. This strategy allows more than two-thirds of the population to delay the screening age without compromising the overall QALYs gained.

While benefit of PSA screening for improving survival is obvious, substantial unfavourable effects still exist.^12^ We calculated the extra managements (extra test, extra biopsies and extra treatments would not be performed in no-screening scenario) to quantify harm-benefit trade-off (Supplementary Table S6). For strategies starting at 45 or 50 years old, age-specific PSA cutoff showed superiority compared with the fixed threshold of 4 ng/mL by improving QALYs gained with less extra diagnoses or treatments used. Using risk-stratified strategies, can substantially reduce the extra managements without compromising QALYs gained. Our most conservative strategies (3 times every five years from 60 to 74 years old) projected 118.44 extra tests, 29.52 extra biopsies and 3.65 extra treatments per life gained. These are similar with results of a study performed in the Bahamas (for a one-time test at 60 years of age, around 74–84 tests, 2.6–3.8 biopsies were cost for each life saved).^37^ In more intensive scenario within high-income countries, for example, screening annually from 55 to 69 years, 916 tests, 32 biopsies and 5 treatments were cost for saving one life.^12^ Strategy with the similar PSA cutoff, screening initiating age and frequency projected by our model yields less PSA tests but more biopsies (PSA cutoff = 4; Age 55–74 Interval 1 year, 585.26 PSA tests, 122.36 biopsies and 5.13 treatments are cost for one life saved).

There is another stratification approach yet to be modelled in our study. Using baseline PSA (PSA test at age 45 yr) threshold of 1.5 ng/mL to stratify risk group can help to avoid 89% unnecessary PSA retesting for 5 years.^38^ Evaluating the cost-effectiveness of such PSA baseline-informed screening strategy represents an important next step for future research. The optimal biopsy strategy also remains controversial. Our study modelled a pathway that MRI is performed prior to biopsy, which has been recommended to reduce the overdiagnosis. However, emerging evidence from the PROBASE study,^39^ suggests that relying solely on MRI-targeted biopsies may miss a proportion of clinically significant prostate cancers (csPCa). This more comprehensive, which is also more costly approach requires further health-economic evaluations.

Two studies have assessed the cost-effectiveness of PSA screening in the Chinese population.^40^^,^^41^ Both evaluated only a single strategy with simple disease outcome model which did not include either natural disease onset and progression or comprehensive post-onset disease transition, lacking a comparison between different screening approaches. Consequently, these analyses may have failed to capture evolving incidence patterns associated with an ageing population, as well as the nuanced costs and utilities linked to different disease stages. To the best of our knowledge, no prior studies have incorporated the potential influence of genetic testing—such as family history or polygenic risk scores—which may influence screening outcomes and cost-effectiveness.

Our study adopted a payer perspective within the Chinese healthcare system. The ICERs reported in our model range from 1458.85 to 5083.31 USD and are substantially lower than observed in developed countries, where ICERs typically range from 31,467 to 72,971 USD.^13^ In another PSA CEA analysis performed in Sweden, using MRI-pathway approach to reduce overdiagnosis in clinically insignificant PCa, the ICER was 53,736 USD per QALY gained.^42^ The primary reason for these considerable differences is medical services costs. For example, the costs of PSA testing, RP, radiotherapy in Netherlands vs. those in China are 39 vs. 3.78, 19,235 vs. 6594 and 23,110 vs. 4550 USD, respectively. However, regional economic variation within China is substantial. The GDP per capita in the most developed area (Shanghai: $26,699.95) is nearly four times that of the least developed region (Gansu: $6738.06). To capture this economic diversity, we gathered cost and pricing data from 28 medical experts across five representative provinces. These regions span a wide range of GDP per capita levels, offering a nationally representative economic sample.

Our study has some strengths. Firstly, the model incorporates key parameters—such as tumour onset hazard, PSA growth slopes, and intercepts—that are inherently unobservable and cannot be directly measured. Unlike previous studies, which may overlook or simplify this challenge, we addressed this by calibrating these parameters using the MCMC method based on local epidemiological data. To enhance robustness, we conservatively calibrated onset hazard and detection hazard. Specifically, we adjusted the model so that the simulated prostate cancer incidence (38.1 per 100,000) was slightly lower than the actual reported rate (45.9 per 100,000), as higher incidence rates tend to bias results in favour of screening by artificially inflating its cost-effectiveness (Supplementary Table S2).

Secondly, we defined eight distinct disease states in the Markov model, reflecting the natural history and clinical prognosis of PCa. To inform the model, we utilised real-world data obtained from a territory-wide electronic medical record system encompassing a 20-year period. Transition probabilities between disease states were derived from a long-term, retrospective, multicentre database, providing a robust and contextually accurate estimation of prostate cancer prognosis within the Chinese healthcare setting (Supplementary Table S3).

Thirdly, this study provided inaugural evidence on the cost-effectiveness of PSA screening incorporating genetic testing for personalised screening. Inherited-risk assessment has become increasingly significant in the context of precision medicine. In this study, we evaluated the cost-effectiveness of two genetic testing strategies—targeted genotyping for PRS and WGS. As screening succeeds and the rate of metastatic presentation declines to level like that in developed countries, the harm-benefit balance of a whole population-based PSA screening strategy would change. And this is one of the primary reasons for incorporating risk-stratified strategies in our study. By incorporating PRS, WGS and family history, screening can be de-intensified for men at lower or average risk, without compromising QALYs gained. This provides an implication that in future China with a reduced rate of metastatic presentation, a personalised strategy could be applied. It is important to note, despite WGS yielding a comparatively higher ICER and modest QALY improvements compared with PRS, it offers comprehensive genetic data that extend beyond prostate cancer and could have value in detecting or managing other conditions. The true value of WGS is a one-time test for lifelong utility. The established large-scale biobank initiatives, in the long run, have demonstrated that WGS can uncover a wealth of clinically relevant genetic information with broad health implications.^43^ Therefore, while not the most efficient strategy when assessed for prostate cancer alone, future studies could assess the cost-effectiveness of incorporating WGS, particularly in economically developed regions, to form a comprehensive, multi-disease preventative health system.

Outside China, population ageing is also driving a sharp rise in PCa burden across other MICs. Global projections indicate that annual incidence will almost double to 2.9 million new cases by 2040, with deaths increasing by 85% over the same period. Unlike high-income countries, where PCa mortality has fallen, MICs are seeing stable or climbing death rates because under-detection results in a high proportion of advanced disease at diagnosis.^44^ Our microsimulation, which covered Chinese provinces spanning a four-fold range in per capita GDP (US $6738–26,700), shows that routine PSA screening remains cost-effective across this economic spectrum. Although unit prices, service availability and treatment capacity differ among countries, the health economics evidence provided here—together with forthcoming real-world experience from China's PSA programme—may serve as a practical reference for MIC policymakers aiming to improve PCa prognosis and population quality of life. However, one discrepancy needs to be mentioned, is the limited use of radical radiation therapy (RT) in management of local-regional stage patients in China. In the Hong Kong setting, patients selected for primary RT are often those considered poor candidates for surgical intervention. Consequently, this patient cohort has an inherently poorer prognosis (Supplementary Table S3. Markov Model Output). Similarly, only around 10% of men in China receive RT as their initial treatment according to our survey and a previous study.^9^ We retained this observed survival rate rather than adjusting it to match external literature from different populations, hope to model the cost-effectiveness of screening within the specific context of local treatment patterns.

Several limitations should be noted. Firstly, while our model relies on disease transition and outcome data derived from real-world evidence, it does not account for grade progression in undetected tumours, primarily due to the absence of longitudinal data on tumour evolution in unscreened individuals. To address this limitation, we assumed a constant distribution of low-risk and high-risk tumours at onset in the Chinese population, based on data from a five-year prospective screening trial. Although this simplification may not fully capture the biological heterogeneity of undiagnosed prostate cancer, one-way sensitivity analyses indicated that variations in these proportions had minimal effect on the ICERs. Secondly, due to China's opportunistic screening environment, we lacked long-term follow-up data from distinct screening and non-screening cohorts, which is important for precisely calculate tumour onset and clinical detection parameters. To implement this, we performed a systematic parameter search for the accurate λo and λc. We used MAE as the measure of fit to ensure the objectivity of this process (Supplementary Table S2), and sensitivity analysis to prove the robustness of our model (Supplementary Methods & Supplementary Table S4). Both the one-way sensitivity analysis and the probabilistic-sensitivity analysis proved that our qualitative conclusions about cost-effectiveness and preferred strategies do not appear to be sensitive to plausible combinations of these parameter values. Thirdly, a key assumption in our model is that while men identified as high-risk by family history or PRS have an increased risk of cancer onset, we do not model a separate worse prognosis for this group. We acknowledge this is a simplification, necessitated by a specific gap in the current evidence. While many studies confirm that high PRS is associated with worse outcomes in men with established or advanced disease, there is a lack of long-term evidence on the natural history and prognosis of cancers detected early as a direct result of risk-stratified screening. Our future work will seek to address this limitation as more granular data becomes available. Fourthly, the active surveillance, as a recommended management guideline for low-risk PCa patients, was not included in our model. Only 2.33% of low-risk patients with PCa in China opt for active surveillance due to cultural differences,^45^ resulting in insufficient data on these individuals. This inevitably introduces a potential risk of overdiagnosis and overtreatment. Our model mimics this management and treatment reality in China. Nevertheless, we used mpMRI in prior of biopsy, an approach has been recommended to reduce overdiagnosis.^42^^,^^46^ Lastly, our model used data from Hong Kong (a developed region in China) to simulate the PCa prognosis pattern, matched with relatively low medical costs of mainland China. However, previous evidence indicated that major urban regions in mainland China have already achieved prostate cancer incidence and mortality rates comparable to those in Hong Kong.^47^^,^^48^ Due to the inequity in health resources and economic conditions, outcomes in less-developed regions appear different. In past decades, a major government initiative was launched to allocate more health resources to less develop regions areas.^49^ In light of these efforts, health insurance coverage surged from 21.0% to 97.4% from 2003 to 2011, and hospital delivery reached 95.9% in 2011 in rural areas.^50^ Therefore, the disparity in PCa prognosis within the country will be continually reduced in the foreseeable future. We have also performed probabilistic sensitivity analysis (Supplementary Figure S5) by varying all transition probabilities across wide ranges to simulate cost-effectiveness outcomes in different scenarios. The results of the sensitivity analysis demonstrates that our conclusions are robust and reliable.

To summarize, PSA screening among Chinese males can improve disease outcomes and overall QALYs while remaining cost-effective. Using age-specific PSA cutoffs is a superior screening strategy compared to using a constant PSA cutoff in improving QALYs across all age groups. Additionally, novel PRS test offers a cost-effective alternative, allowing average-risk individuals to postpone their screening start age or extend screening intervals without compromising QALYs gained. This study provides a solid evidence base for Chinese policymakers to consider establishing a cost-effective, risk-stratified PCa screening programme.

## Contributors

All authors contributed to the study conception. Jiacheng Liu, Yuanshi Jiao, Xiaohao Ruan, Yongle Zhan, Salida Ali, Da Huang, Xue Li and Rong Na designed the methodology. Jiacheng Liu and Yuanshi Jiao performed software and formal analysis, cross-verified the analysis. Resources were organised by Jiacheng Liu, Yueting Huang, Yuanshi Jiao, Ruocheng Ma and Rong Na. Jiacheng Liu and Yuanshi Jiao conducted the investigation. Jiacheng Liu, Rong Na, Yuanshi Jiao, Xue Li, Yueting Huang and Di Gu had access to raw data. Data Curation was performed by Yuanshi Jiao, Yueting Huang, Yongle Zhan, Ruofan Shi, Xiaohao Ruan, Chi Yao, Ruocheng Ma, Tsun Tsun Chun, Danfeng Xu, Qian Zhang, Di Gu, Xue Li and Rong Na. Jiacheng Liu, Yuanshi Jiao, and Yueting Huang verified data. Jiacheng Liu drafted the initial manuscript, and all authors commented on subsequent versions of the manuscript. Jiacheng Liu and Rong Na are the guarantors of this manuscript. All authors had read and approved the final manuscript.

## Data sharing statement

Datasets generated and analysed in this study are available from the corresponding authors on reasonable request.

## Editor note

The Lancet Group takes a neutral position with respect to territorial claims in published maps and institutional affiliations.

## Declaration of interests

AstraZeneca supported the coordination among 28 experts nationwide to facilitate our team's collection of treatment patterns and pricing data via a survey. No financial sponsorship was received from AstraZeneca. All authors declare that they have no competing interests.
