# Supplementary material for: Cost–effectiveness analysis of prostate-specific antigen screening in China: a middle-income population-based microsimulation study
Source: Lancet Reg Health West Pac. 2025 Sep 16;62:101683. doi: 10.1016/j.lanwpc.2025.101683 (PMC12465055; doi:10.1016/j.lanwpc.2025.101683)
Supplement: Supplementary Materials [file mmc1.docx]

**Index**

[Details of model 3](#_Toc195391551)

[Prostate cancer incidence model 3](#_Toc195391552)

[Decision tree 3](#_Toc195391553)

[Prognosis Markov model 3](#_Toc195391554)

[Survey information 3](#_Toc195391555)

[Time horizon 4](#_Toc195391556)

[Model Validation 4](#_Toc195391557)

[Supplementary Figure 1. China Population Distribution Changing Trend 5](#_Toc195391558)

[Supplementary Figure 2. PSA Growth Trend 6](#_Toc195391559)

[Supplementary Figure 3a. Incidence rate among men aged 45-84 7](#_Toc195391560)

[Supplementary Figure 3b. Local-regional Incidence rate among men aged 45-84 7](#_Toc195391561)

[Supplementary Figure 3c. Metastasis rate among men aged 45-84 8](#_Toc195391562)

[Supplementary Figure 3d. Metastasis/Incidence ratio among men aged 45-84 8](#_Toc195391563)

[Supplementary Figure 4. One‐way Sensitivity Analysis 9](#_Toc195391564)

[PSA Cutoff 4, Age 45-74, Interval 1 year 9](#_Toc195391565)

[PSA Cutoff 4, Age 45-74, Interval 2 years 10](#_Toc195391566)

[PSA Cutoff 4, Age 45-74, Interval 3 years 11](#_Toc195391567)

[PSA Cutoff 4, Age 45-74, Interval 5 years 12](#_Toc195391568)

[PSA Cutoff 4, Age 50-74, Interval 1 year 13](#_Toc195391569)

[PSA Cutoff 4, Age 50-74, Interval 2 years 14](#_Toc195391570)

[PSA Cutoff 4, Age 50-74, Interval 3 years 15](#_Toc195391571)

[PSA Cutoff 4, Age 50-74, Interval 5 years 16](#_Toc195391572)

[PSA Cutoff 4, Age 55-74, Interval 1 year 17](#_Toc195391573)

[PSA Cutoff 4, Age 55-74, Interval 2 years 18](#_Toc195391574)

[PSA Cutoff 4, Age 55-74, Interval 3 years 19](#_Toc195391575)

[PSA Cutoff 4, Age 55-74, Interval 5 years 20](#_Toc195391576)

[PSA Cutoff 4, Age 60-74, Interval 1 year 21](#_Toc195391577)

[PSA Cutoff 4, Age 60-74, Interval 2 years 22](#_Toc195391578)

[PSA Cutoff 4, Age 60-74, Interval 3 years 23](#_Toc195391579)

[PSA Cutoff 4, Age 60-74, Interval 5 years 24](#_Toc195391580)

[Age-specific PSA Cutoff, Age 45-74, Interval 1 year 25](#_Toc195391581)

[Age-specific PSA Cutoff, Age 45-74, Interval 2 years 26](#_Toc195391582)

[Age-specific PSA Cutoff, Age 45-74, Interval 3 years 27](#_Toc195391583)

[Age-specific PSA Cutoff, Age 45-74, Interval 5 years 28](#_Toc195391584)

[Age-specific PSA Cutoff, Age 50-74, Interval 1 year 29](#_Toc195391585)

[Age-specific PSA Cutoff, Age 50-74, Interval 2 years 30](#_Toc195391586)

[Age-specific PSA Cutoff, Age 50-74, Interval 3 years 31](#_Toc195391587)

[Age-specific PSA Cutoff, Age 50-74, Interval 5 years 32](#_Toc195391588)

[Age-specific PSA Cutoff, Age 55-74, Interval 1 year 33](#_Toc195391589)

[Age-specific PSA Cutoff, Age 55-74, Interval 2 years 34](#_Toc195391590)

[Age-specific PSA Cutoff, Age 55-74, Interval 3 years 35](#_Toc195391591)

[Age-specific PSA Cutoff, Age 55-74, Interval 5 years 36](#_Toc195391592)

[Age-specific PSA Cutoff, Age 60-74, Interval 1 year 37](#_Toc195391593)

[Age-specific PSA Cutoff, Age 60-74, Interval 2 years 38](#_Toc195391594)

[Age-specific PSA Cutoff, Age 60-74, Interval 3 years 39](#_Toc195391595)

[Age-specific PSA Cutoff, Age 60-74, Interval 5 years 40](#_Toc195391596)

[Supplementary Figure 5. Probabilistic-Sensitivity Analysis 41](#_Toc195391597)

[Supplementary Table 1. Multipliers for Tumour Onset Hazard among High-Risk Populations 42](#_Toc195391598)

[Supplementary Table 2. Validation of the Fred Hutchinson Cancer Research Centre Prostate Cancer Incidence Model Against WHO Estimates in China 43](#_Toc195391599)

[Supplementary Table 3. Markov Model Output 44](#_Toc195391600)

[Supplementary Table 4. Full Incremental Cost‐Effectiveness Results for All Simulated Prostate Cancer Screening Strategies 45](#_Toc195391601)

[Supplementary Table 5. Robustness Analysis: The Influence of High-Risk Group Proportion on Cost‐Effectiveness Results 49](#_Toc195391602)

[Reference 51](#_Toc195391603)

# Details of model

## Prostate cancer incidence model

The Fred Hutchinson Cancer Research Centre Prostate Cancer Incidence Model was developed as part of the Cancer Intervention and Surveillance Modelling Network (<http://cisnet.cancer.gov>).

It is fundamentally based on these assumptions: (a) The logarithm of PSA (denoted as P) growth is linearly correlated with age and the state of disease $P\left( t \right)=b+a_{0}t+a_{x}t\left( t-t_{o} \right)I\left( t>t_{o}i \right)+\varepsilon$, $I\left( \cdot\right)$ is an indicator function, $t_{o}$ denotes the age at onset of a preclinical tumour, and and $a_{x}$takes the value $a_{1}$or$a_{2}$ depending on the Gleason Grade of the disease. (b) Disease progression is driven by age or PSA growth. Specifically, a hazard of disease onset is proportional to age $\lambda_{o}\left( t \right)=\gamma_{o}t$, while hazards of metastasis $\lambda_{m}\left( t \right)=exp\left( P \right)\gamma_{m}$ and clinical detection $\lambda_{c}\left( t \right)=exp\left( P \right)\gamma_{c}$ The hazard of clinical detection increases once the tumour metastasizes: $\lambda_{c}\left( t \right)=exp\left( P \right)\gamma_{c}\theta_{c}$ . $\gamma_{LR}$ is the proportion of patients with tumour Gleason Grade Group ≤ 3.

**Healthy PSA Growth Slope (a0)**: Using a linear regression model of log (PSA) against age, we calculated α0 of 0.0215 (95% CI: 0.0210–0.0220) based on the 5-year prospective screening trial. This finding aligns closely with a previously published pre-onset slope of 0.0200 (95% CI: 0.0188–0.0212) ^1^. While some other well-conducted studies have applied the reported parameter directly in different populations to mitigate verification bias^2–5^, the strong consistency evidenced by the overlapping confidence intervals, enhances the robustness of our model.

**Post-onset PSA growth slopes for low-risk (a1) and high-risk (a2)**: We estimated the post-onset PSA growth slopes for low-risk (a1) and high-risk (a2) cancer using a MCMC methodology. A mean sojourn time (MST, defined as the period in which the tumour has been onset but not clinically detected yet) of 11.3–12.6 years was reported by the UK ProtecT trial^6^, and 11.77 - 13.80 years by FRED HUTCHINSON CANCER RESEARCH CENTRE (PSAPC)^7^. Given the higher proportion of advanced-stage disease at diagnosis in the Chinese population^8^, and developing healthcare system compared with that in developed country^9^, we used a conservative MST as the prior in our MCMC simulation.

**λo and λc**: We performed a systematic parameter search, began with initial estimates for the onset rate and clinical diagnosis parameter, using a range of factors (from 1.0 to 4.0, in strides of 0.2) to multiply or to divide the initial parameters until the lowest Mean Absolute Error (MAE) was reached (**Supplementary Table 2**).

**θc:** We considered a variant of the model in which the hazard of clinical detection may differ for localized and metastatic tumours. This parameter was directly obtained from the original study in which constructed this model^1^.

**λ_LR_ and λ_HR_:** Our model relies on disease transition and outcome data derived from real-world evidence, it does not account for grade progression in undetected tumours, primarily due to the absence of longitudinal data on tumour evolution in unscreened individuals. To address this limitation based on patient proportion from the five-year prospective screening trial, we assumed disease grade (low- or high-risk) is determined at onset, proportioned to λ_LR_ and λ_HR_, and does not change over time^7^. The proportion was calculated based on data from the five-year prospective screening trial.

In brief, the model assumes the tumour onset hazard increases linearly with age, while PSA level increases exponentially with age in males. Following the PCa tumour onset, PSA growth accelerates **(Supplementary Figure 2)**, and the hazard of tumour clinical detection (due to symptoms, incidental findings, etc.) and metastasis which are linearly correlated to individual’s PSA level would increase correspondingly. Additionally, the probability of being clinically detected further increases after a metastasis event **(Table 1)**.

## Decision tree

Decision Tree is employed in both the screening part and the treatment part of the model.

During screening procedure, individuals with PSA level above the designated cutoff but below 10 ng/mL undergo MRI examination prior to biopsy. Based on MRI outcome, a decision is made regarding the necessity of further biopsy.

The performance of MRI is determined based on the threshold of PI-RADS 4^10,11^. The biopsy approach is defined as MRI followed by cognitive MRI-targeted biopsy (for PSA > 4 ng/mL & PI-RADS ≥ 4) or systematic biopsy (for PSA > 10 ng/mL) (no significant differences between targeted and systematic biopsy according to the study) ^12^. The sensitivity of biopsy was defined as 64%. According to a systematic review, the specificity of MRI pathway biopsy is 1.00 [95% CI 0.90–1.00] ^13^.

For patients with pathologically confirmed prostate cancer, subsequent categorization leads them to either the surgery group or the radiotherapy group for treatment. The ratio of surgery and radiotherapy is based on a survey of 28 urology experts from different regions across the country, reflecting both local specific treatment patterns and international guidelines^14,15^.

## Prognosis Markov model

Based on the natural disease progression procedure, we set 7 disease states: monitoring post RP, monitoring post RT, biochemical recurrence (BCR) after RP, BCR after RT, metastatic hormone-sensitive prostate cancer (mHSPC), none-metastatic castration-resistant prostate cancer (nmCRPC) and metastatic castration-resistant prostate cancer (mCRPC). Transition probabilities (including survival) between eight states in the Markov model were generated from retrospective analysis of territory-wide electronic medical records (EMR) administered by the Hong Kong Hospital Authority (HA). The transition probabilities for the model were derived directly from this empirical real-world database. We calculated these probabilities by identifying patients' disease states and tracking the observed transitions between them.

## Survey information

We conducted a survey among 28 urology experts from different districts across the country, from the least developed to the most developed region including Gansu, Hunan, Guangdong, Jiangsu and Shanghai, so as to obtain a comprehensive understanding of PCa treatment landscape in China. Proportion of treatment, all the medicine prices and costs of medical services including tests, examinations and interventions (PSA test, MRI, biopsy, RP and RT) in this study were mainly derived from this survey.

In China, the medical service prices in each province are guided by the central government. The costs of medical services were cross-checked and calibrated according to the *National Technical Specifications for Medical Service Projects* (in Chinese) and *Medical Service Price Catalogue* (in Chinese). Medicine costs were also cross checked with publicly available information. Given the variability in drug prices from different supply channels, the purpose of the cross-checking procedure was to exclude obvious outliers (exceeding 1.5 times the interquartile range from the median price). The final cost inputs incorporated expert-derived data, calibrated and adjusted based on official references where appropriate, enhancing the representativeness and robustness of the model inputs.

Utilities applied in Markov model is derived from a multi-national, longitudinal, observational study performed in tertiary hospitals in in eight Asian countries/regions, illustrating a comprehensive picture of quality-of-life among Asian PCa patients.

## Time horizon

The model was initialized using a multi-age cohort of male in China in 2020, which derives from the China Population Census Book 2020. Our simulation specifically focuses on males aged 35 and older, as the probability of prostate cancer onset is hypothesized to be 0 among male younger than 35 years old according to Fred Hutchinson Cancer Research Centre microsimulation model^7^.

The population changes were simulated on an annual cycle. At the end each simulation cycle, the number of individuals in each age group was adjusted based on the age-specific all-cause mortality rates, which were also derived from the China Population Census Book 2020. This subtraction of individuals simulates the natural death patterns within the population as it ages over time. According to our simulation illustrated in **Supplementary Figure 1**, the area under curve among the old male (>60 years old) is increasing along with the time. This indicates that aging is an ongoing phenomenon in China, and it is the primary driver of increasing prostate cancer burden in China.

To ensure we could capture the full range of clinical events in this 30-year horizon, we first ran 100 cycles (representing a life-long span) to allow the model’s key states to reach a steady-state distribution across different age groups. Only after this equilibrium was established, had we begun the actual cost-effectiveness analysis. The incidence, mortality, case-fatality rates of each year were adjusted to the age-group population in that year.

## Model Validation

We adopted a conservative approach in calibrating the Fred Hutchinson Cancer Research Centre prostate cancer incidence model, deliberately underestimating the incidence of PCa to strengthen the robustness of the model's outcomes (Supplementary Table 2). The survival estimates of each disease state, as simulated by Markov model are reported in Supplementary Table 3.


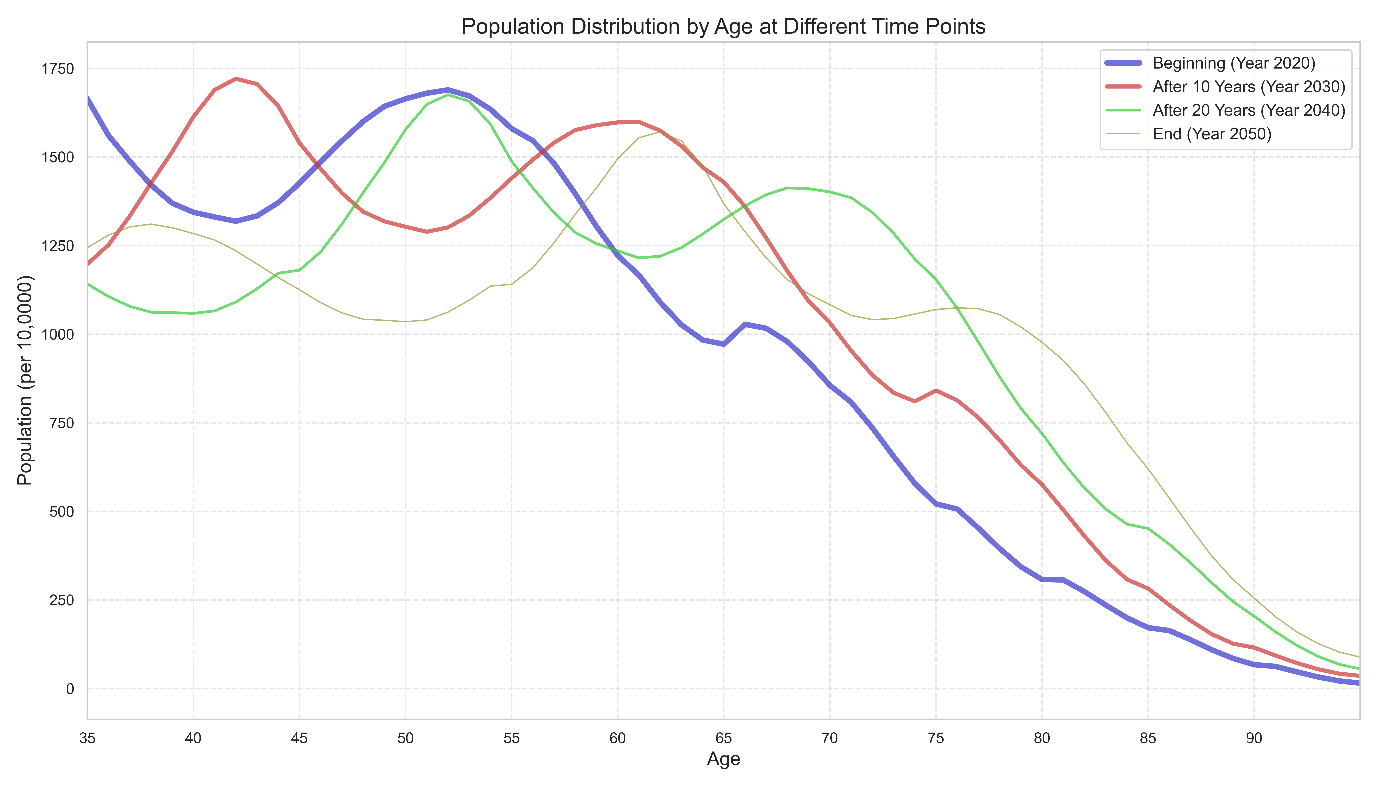


# Supplementary Figure 1. China Population Distribution Changing Trend

Each coloured curve corresponds to a specific year, with the thicker blue curve representing the distribution in 2020 and progressively thinner curves showing projections for 2030, 2040, and 2050. The area under curve represents the population of certain age group. Data derive from the China Population Census Book 2020. ^16^


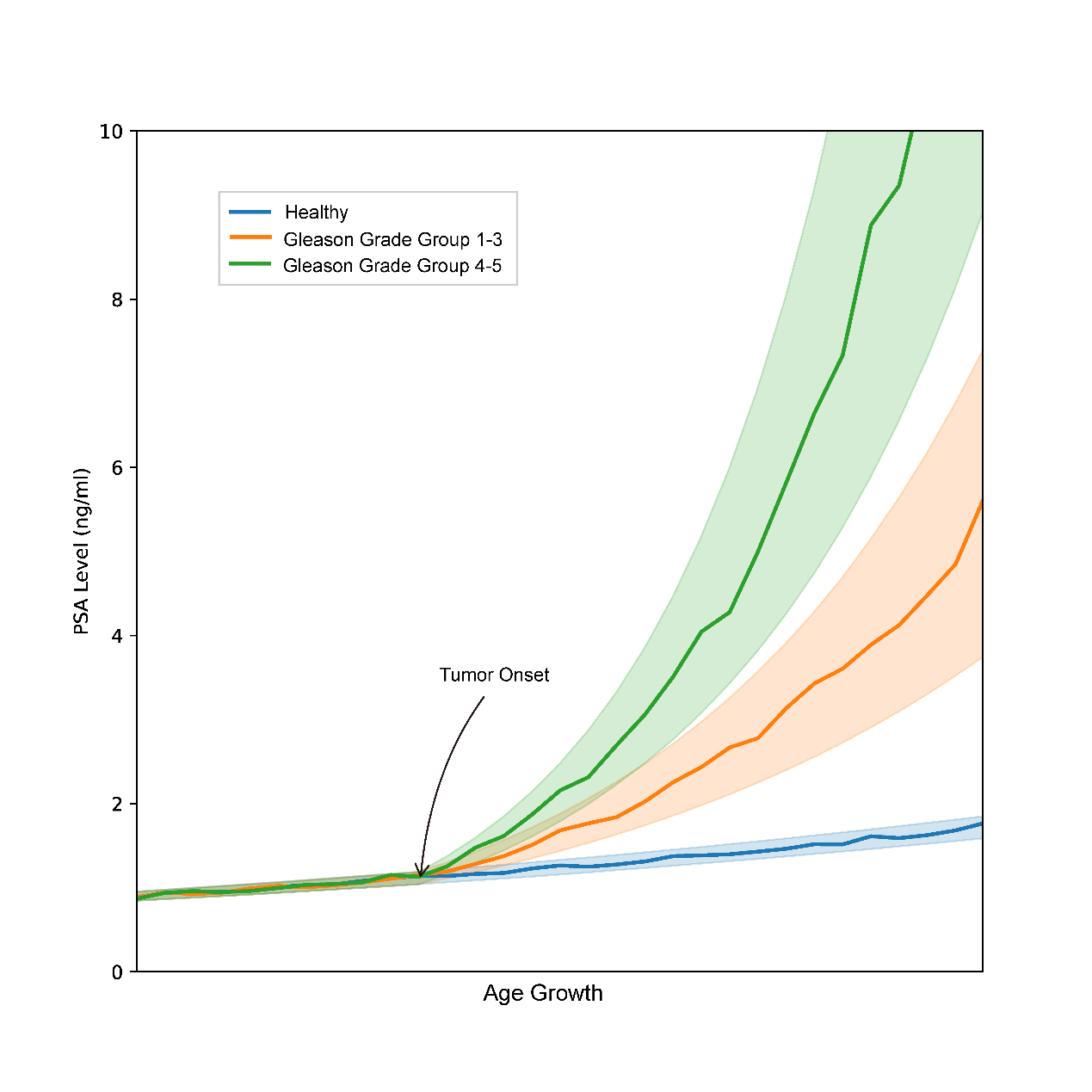


# Supplementary Figure 2. PSA Growth Trend

The figure shows the simulated PSA progression trends within the model. The blue line illustrates the PSA trajectory with age in healthy individuals. The orange line represents the PSA trend for individuals after tumour onset with Gleason Grade Group 1–3, while the green line represents those with Gleason Grade Group 4–5. Shaded bands around those lines illustrate between-person variability in PSA values based on 95% CI ranges.


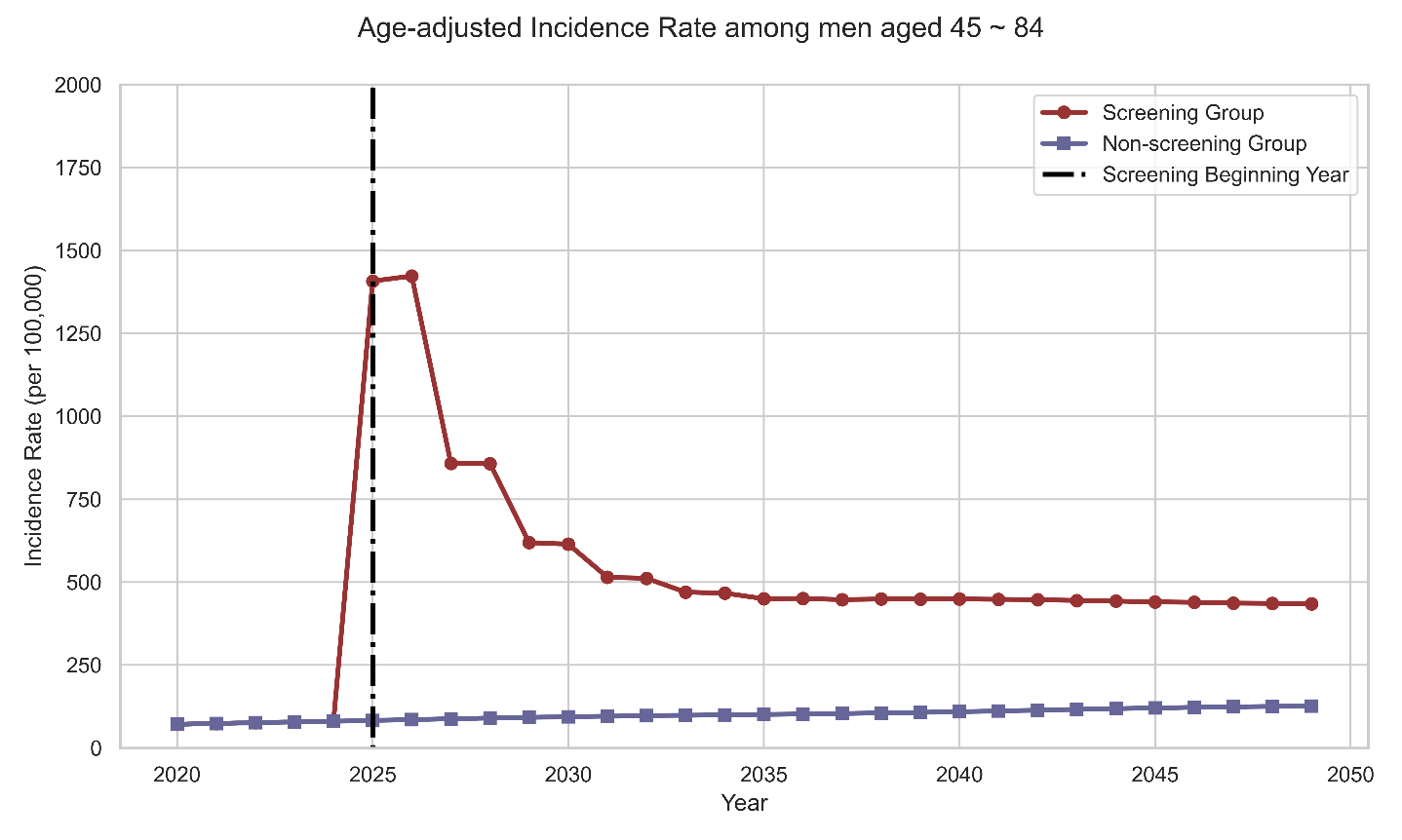


# Supplementary Figure 3a. Incidence rate among men aged 45-84

Annual incidence rates (age-adjusted) of prostate cancer among men aged 45–84 under screening (red line) versus no screening (blue line) from 2020 to 2050. The simulated screening starts in 2025 (The black dashed line). Rates are expressed per 100,000 men.


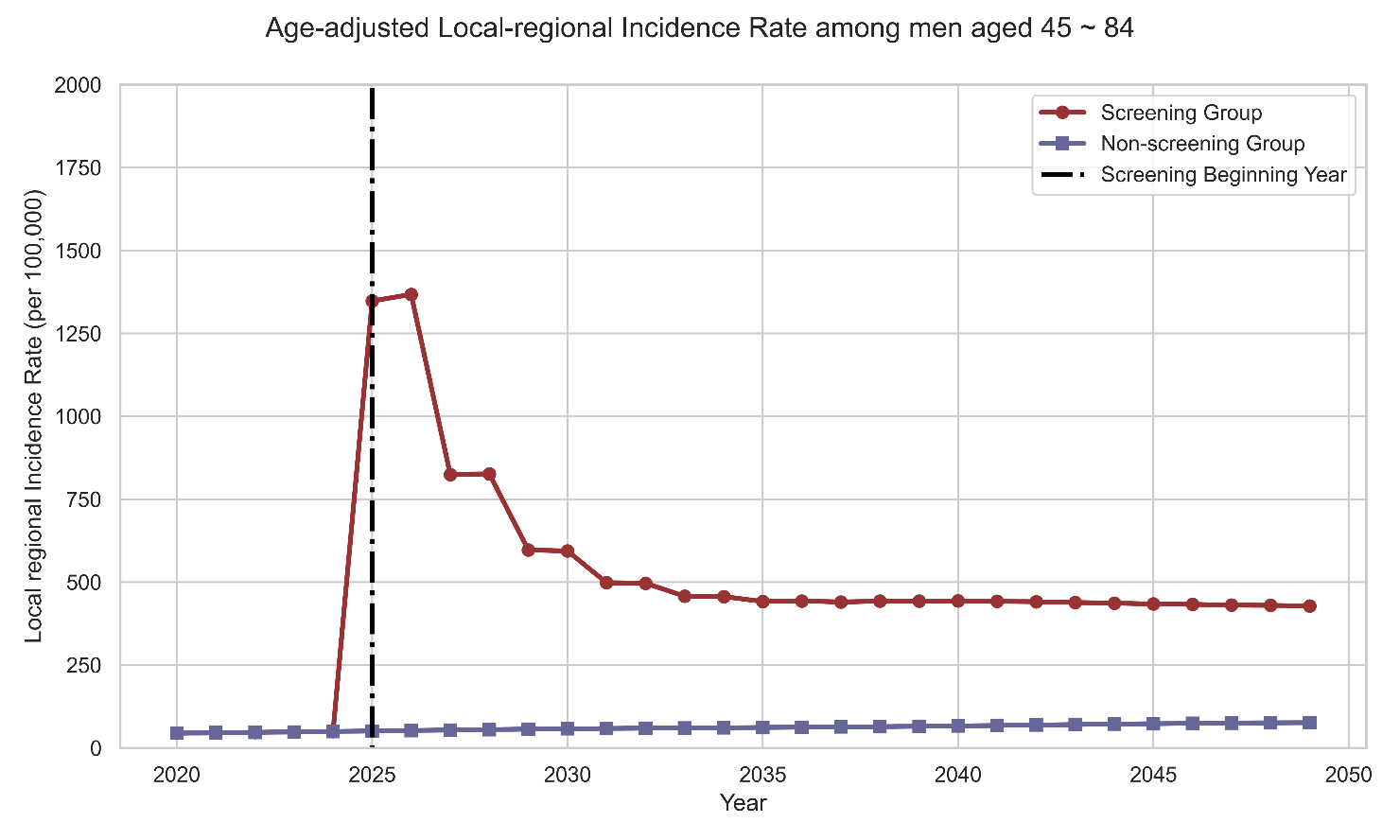


# Supplementary Figure 3b. Local-regional Incidence rate among men aged 45-84

Annual incidence rates of local-regional prostate cancer among men aged 45–84.


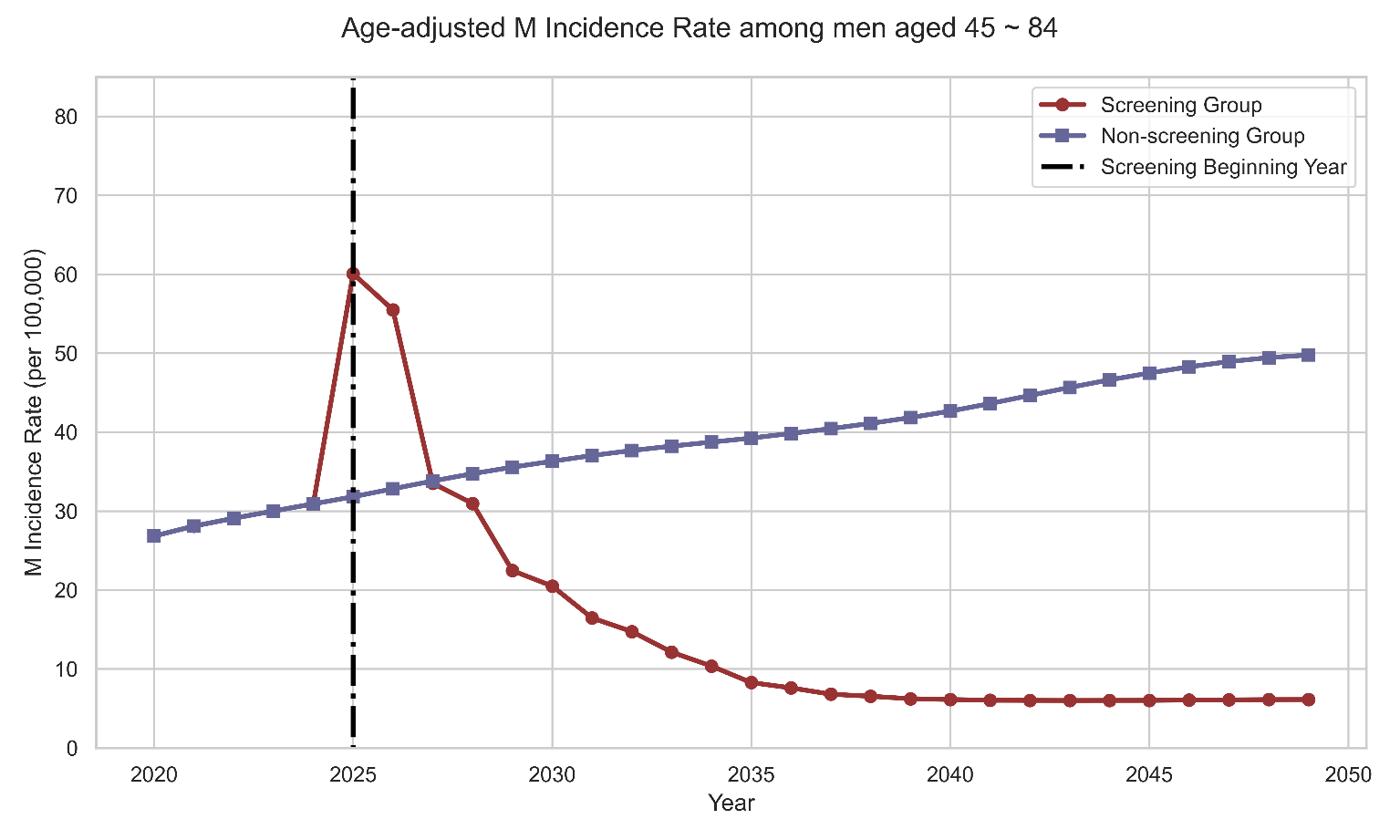


# Supplementary Figure 3c. Metastasis rate among men aged 45-84

Annual incidence rates of metastatic prostate cancer among men aged 45–84.


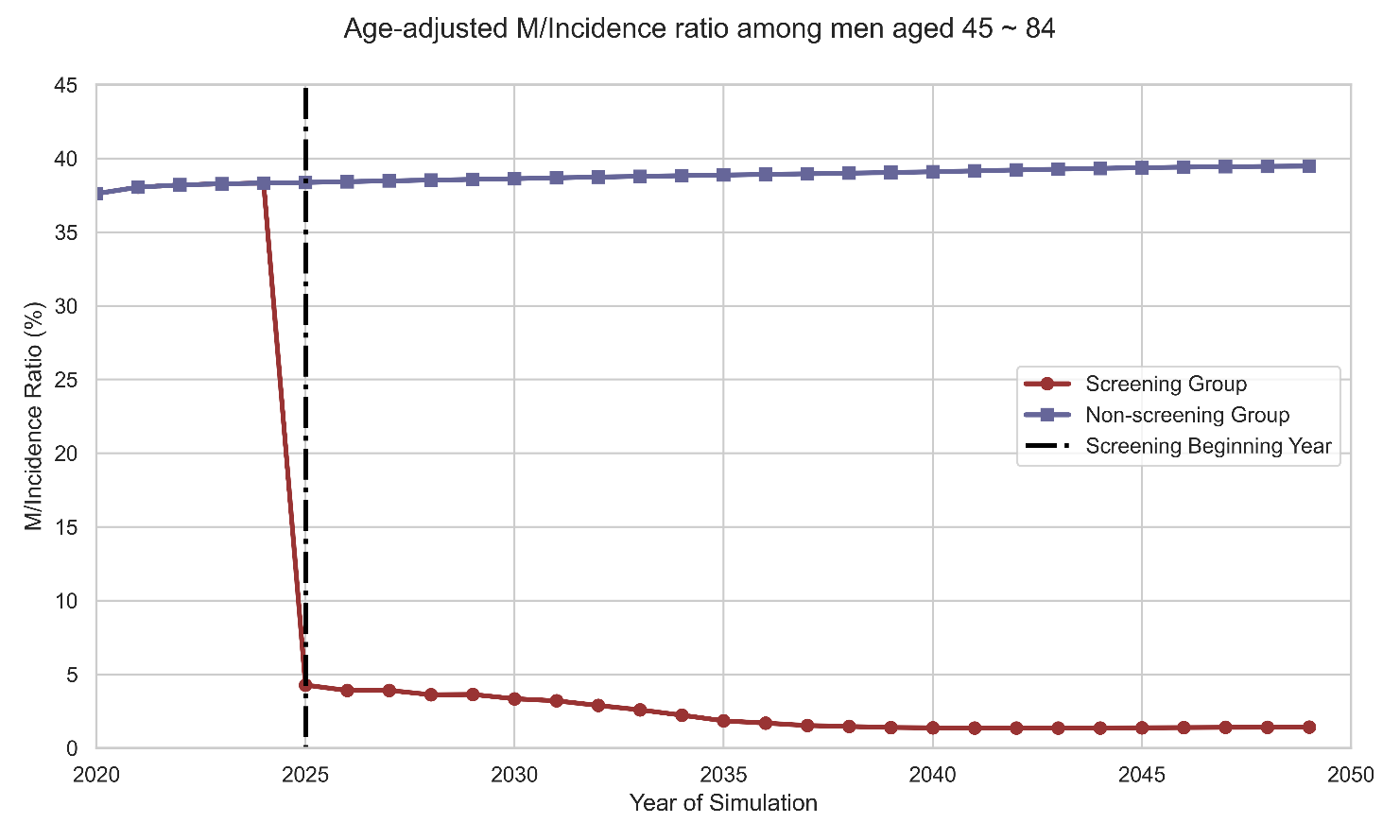


# Supplementary Figure 3d. Metastasis/Incidence ratio among men aged 45-84

Metastasis‐to‐incidence ratio among men aged 45–84 from 2020 to 2050.

# Supplementary Figure 4. One‐way Sensitivity Analysis

## PSA Cutoff 4, Age 45-74, Interval 1 year


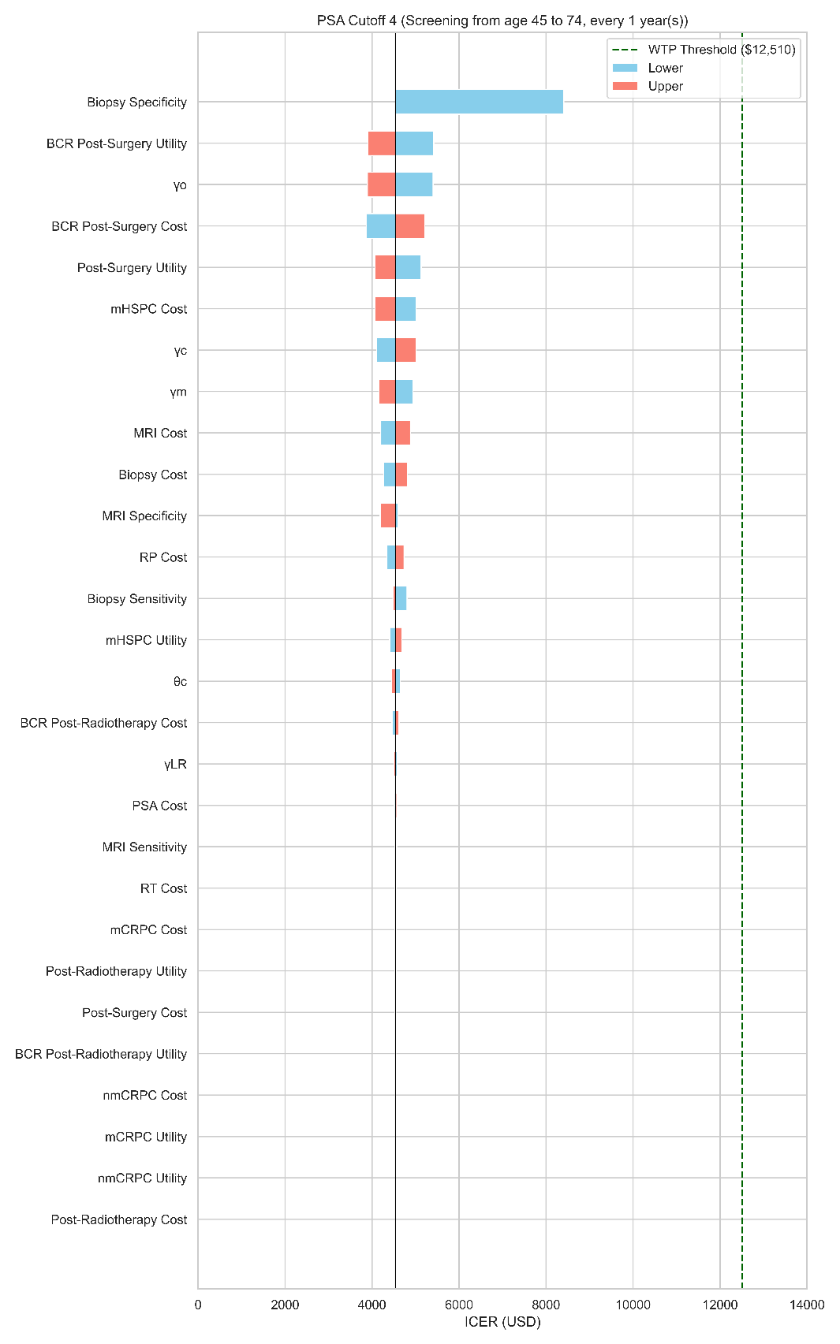


Determinant Parameters Sensitivity Analysis showing the impact of key model parameters on the ICERs of annual PSA screening (ages 45–74) at a cutoff of 4 ng/mL. The horizontal axis denotes the ICER in USD; the vertical black line represents the base‐case estimate. Each bar illustrates how varying a single parameter to its upper (red) or lower (blue) bound shifts the ICER from this base‐case value. Parameters at the top exert the greatest influence, while those at the bottom have a lesser effect.

γo (hazard of tumour onset); γm (hazard of tumour metastasis); γc (hazard for a tumour being clinically detected); γLR (proportion of low - risk cancers); θc (multiplier for clinical detection hazard after metastasis); a0 (PSA growth slope in the healthy individuals); a1 (PSA growth slope in low - risk tumours); a2 (PSA growth slope in high - risk tumours). BCR, biochemical recurrence; mHSPC, metastatic hormone‐sensitive prostate cancer; nmCRPC, nonmetastatic castration‐resistant prostate cancer; mCRPC, metastatic castration‐resistant prostate cancer.

## PSA Cutoff 4, Age 45-74, Interval 2 years


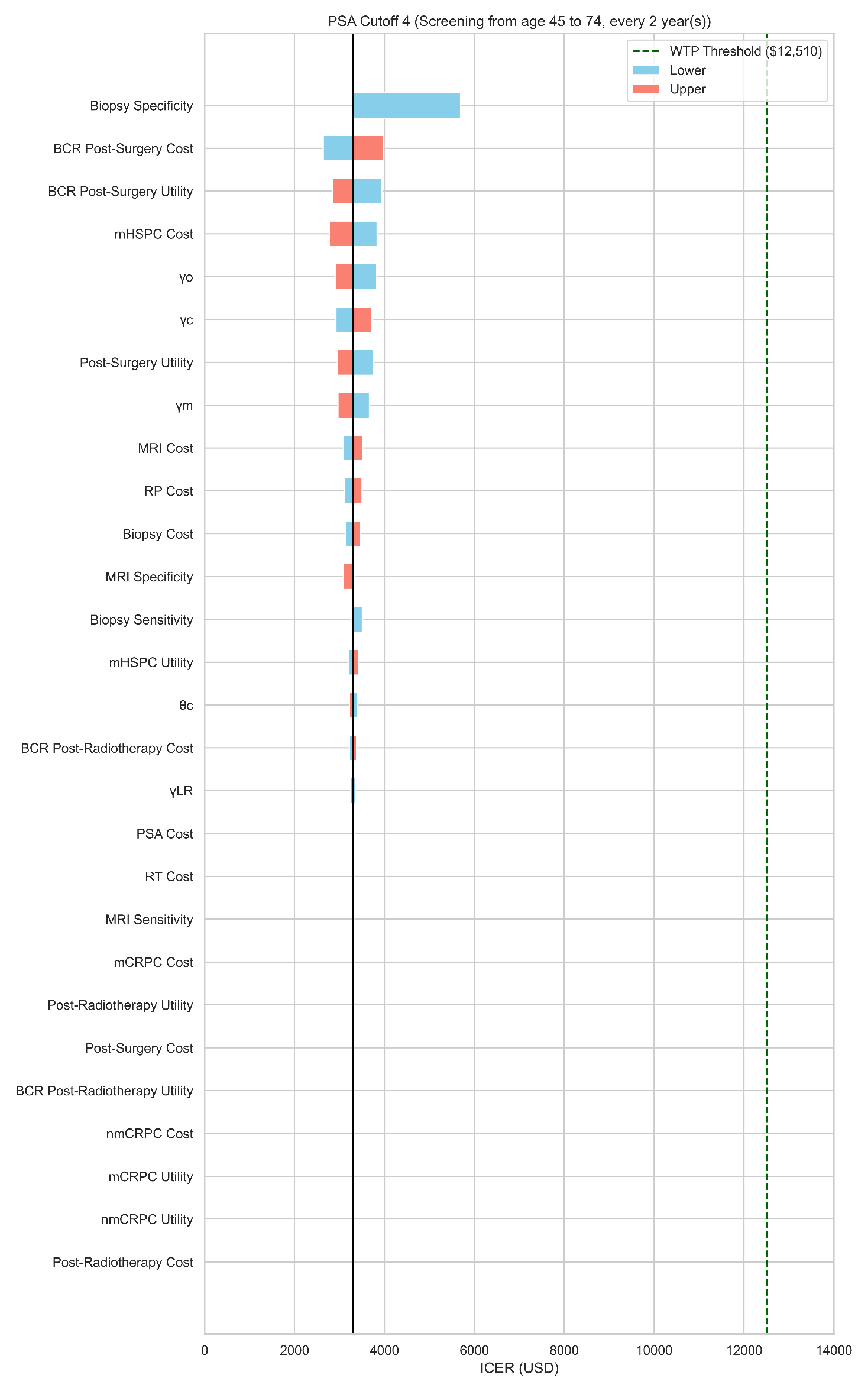


## PSA Cutoff 4, Age 45-74, Interval 3 years


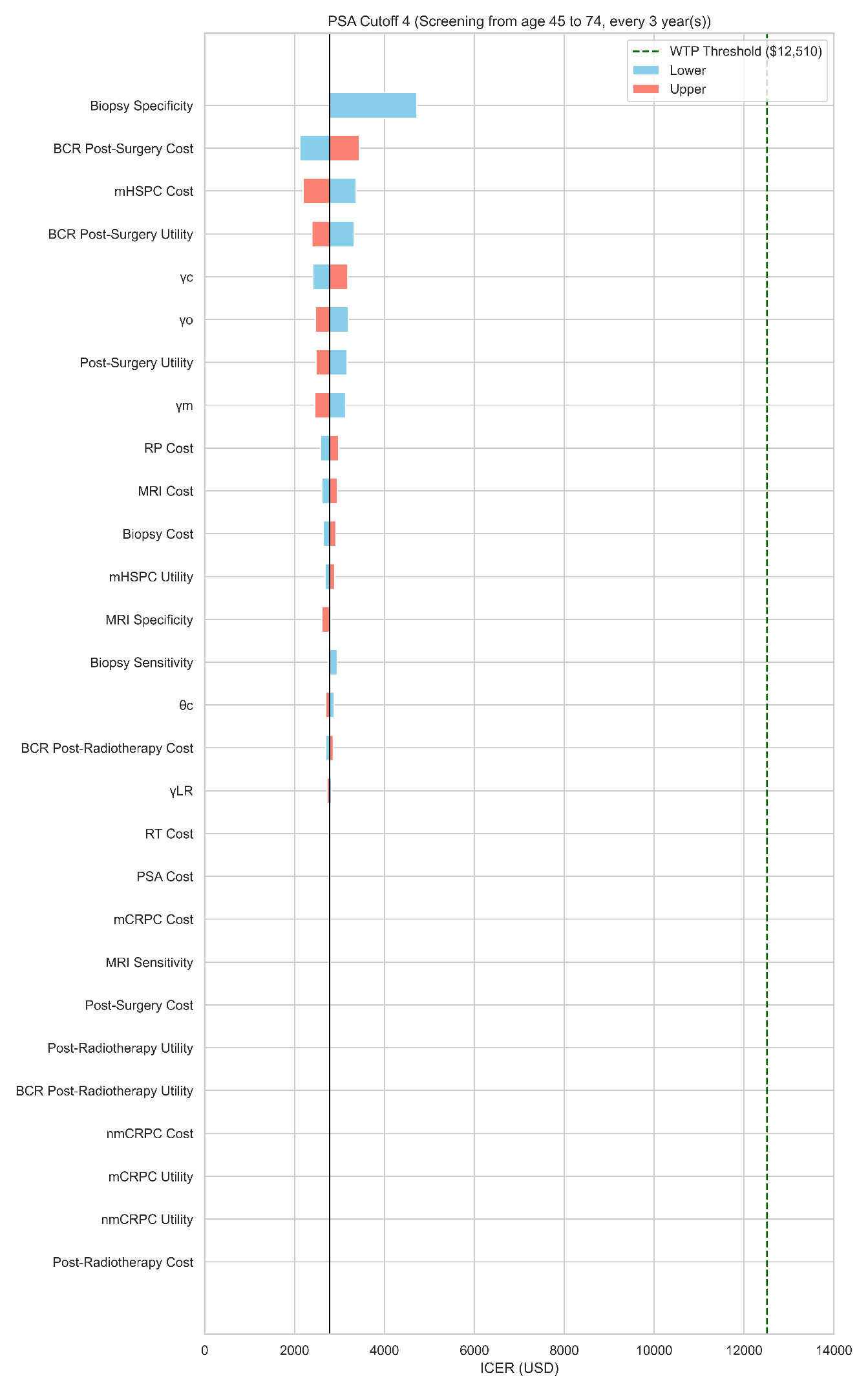


## PSA Cutoff 4, Age 45-74, Interval 5 years


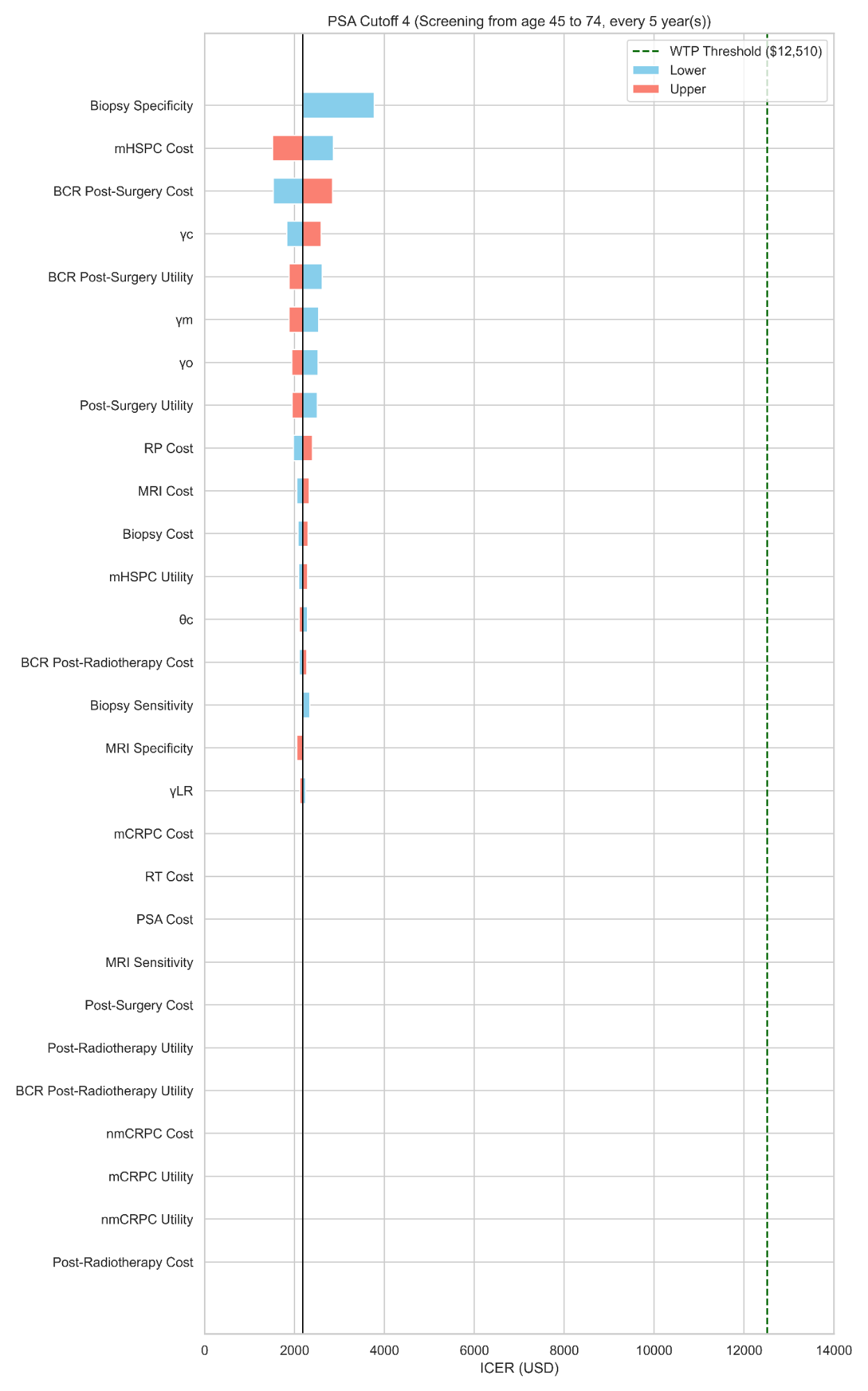


## PSA Cutoff 4, Age 50-74, Interval 1 year


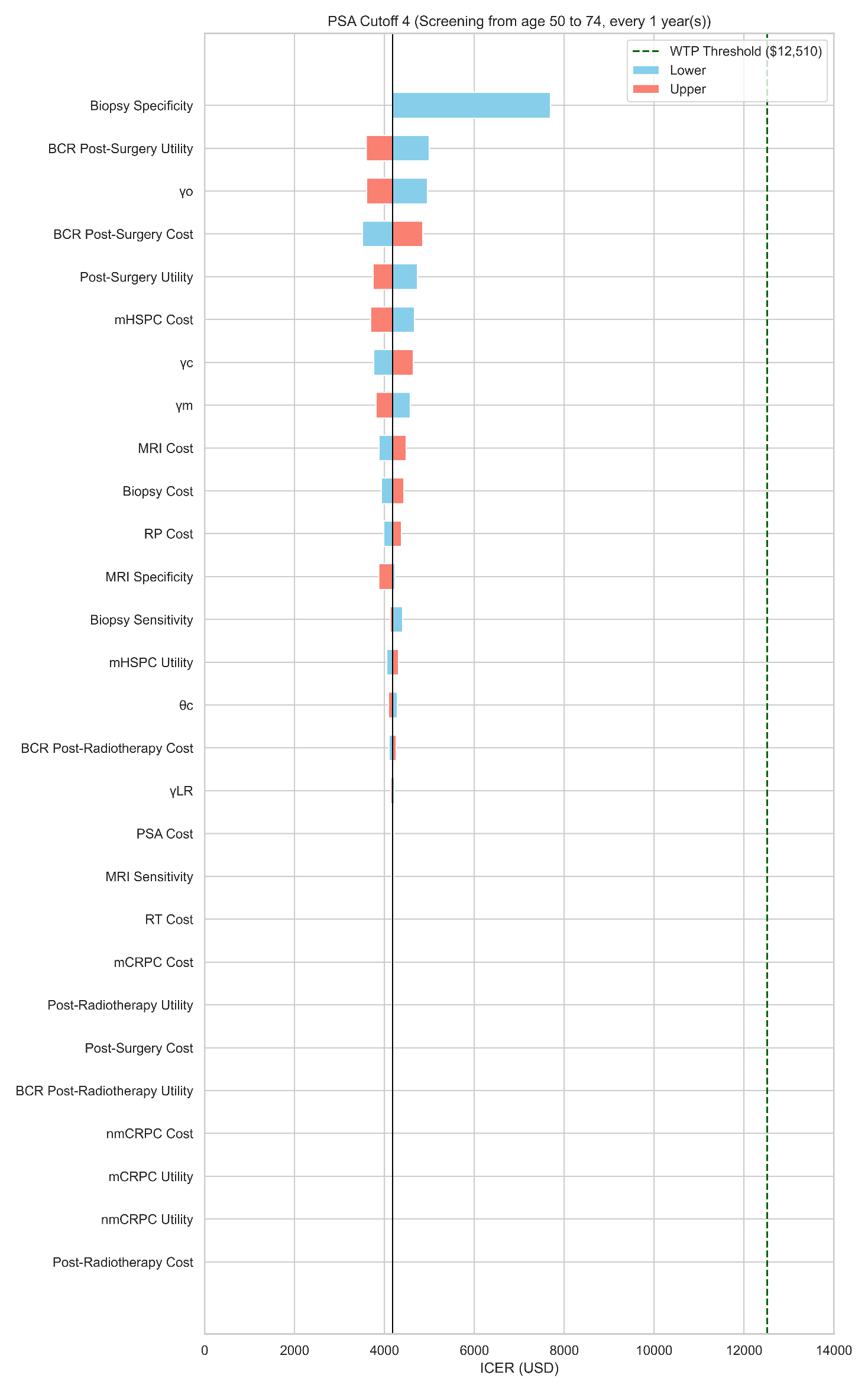


## PSA Cutoff 4, Age 50-74, Interval 2 years


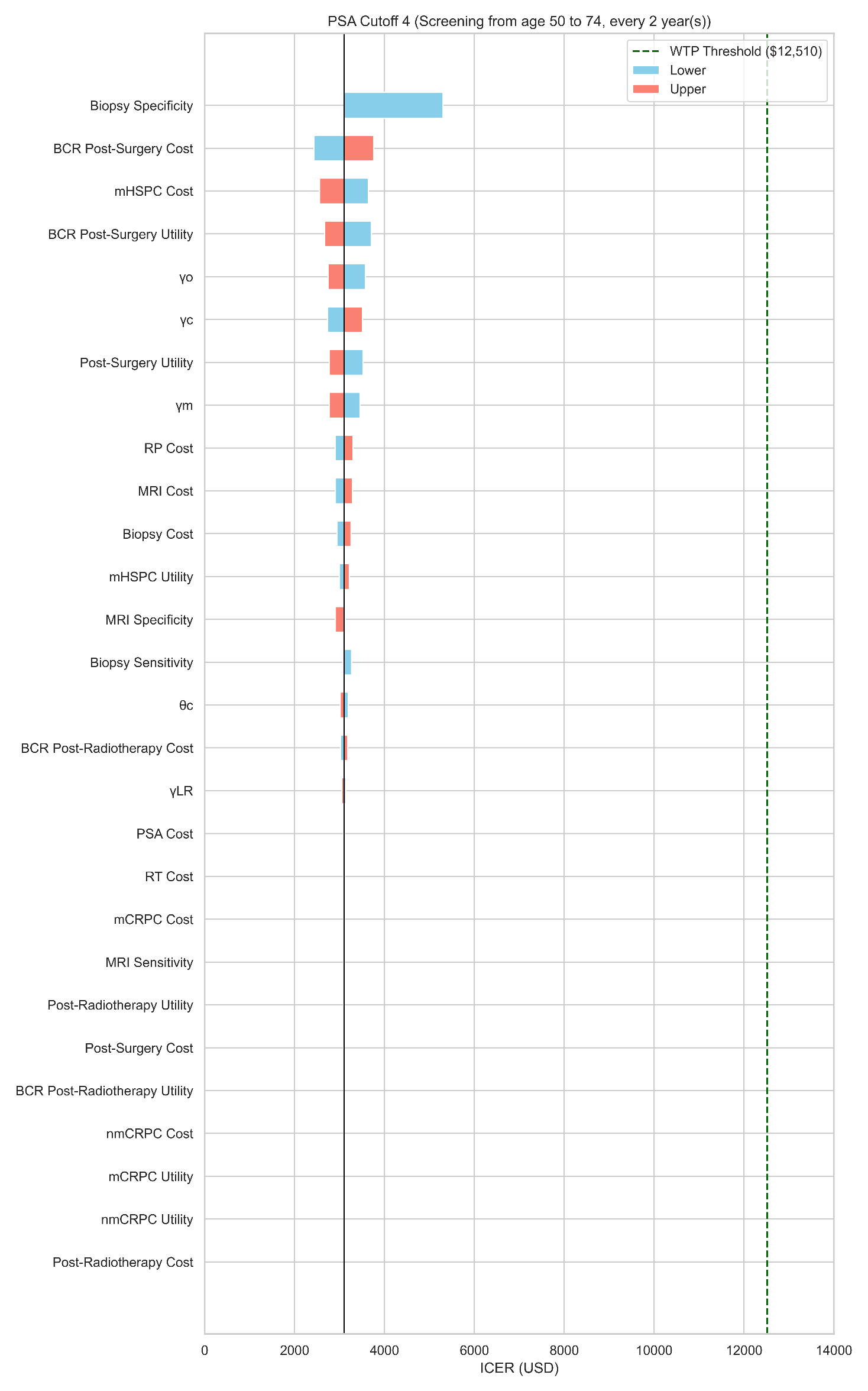


## PSA Cutoff 4, Age 50-74, Interval 3 years


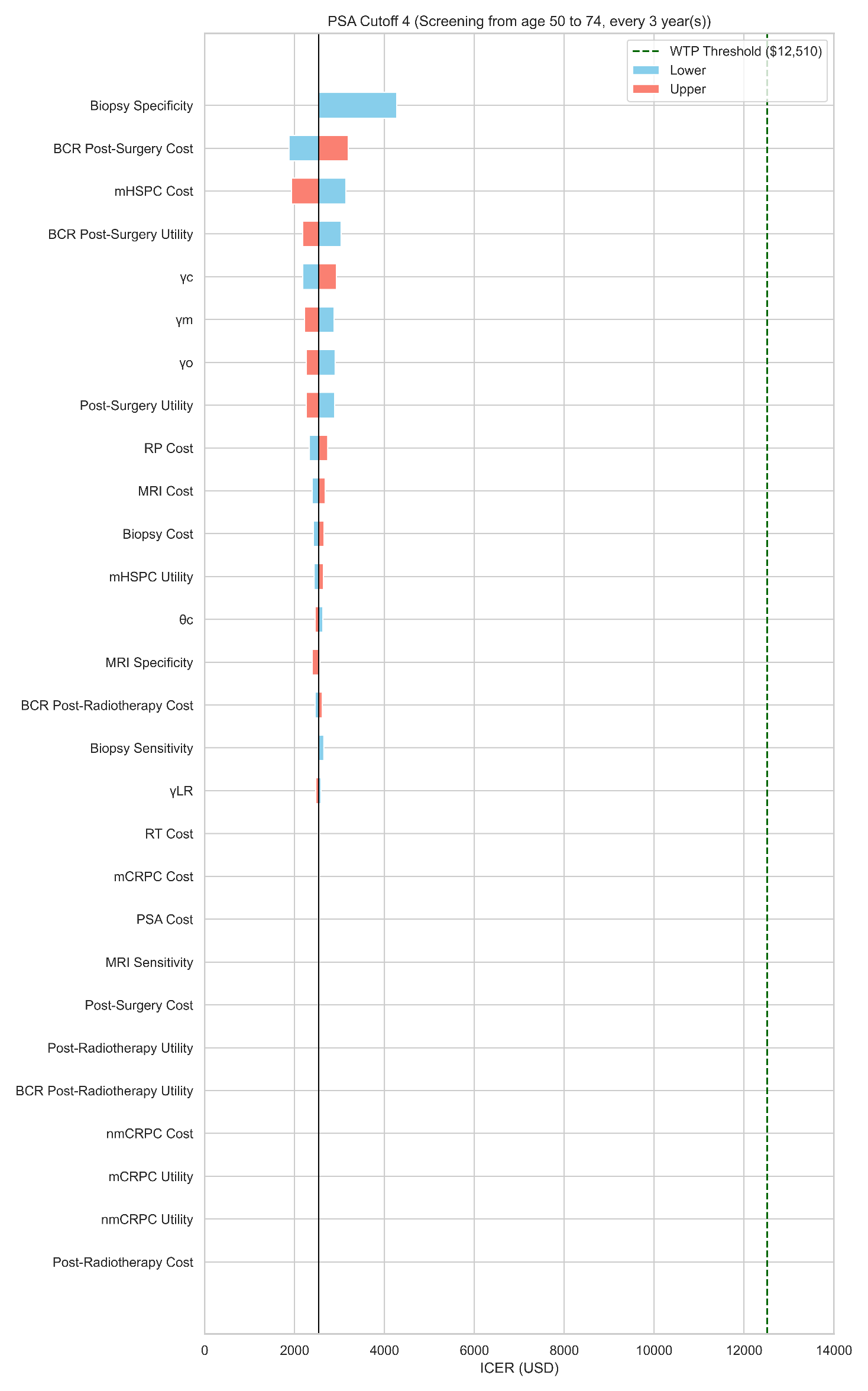


## PSA Cutoff 4, Age 50-74, Interval 5 years


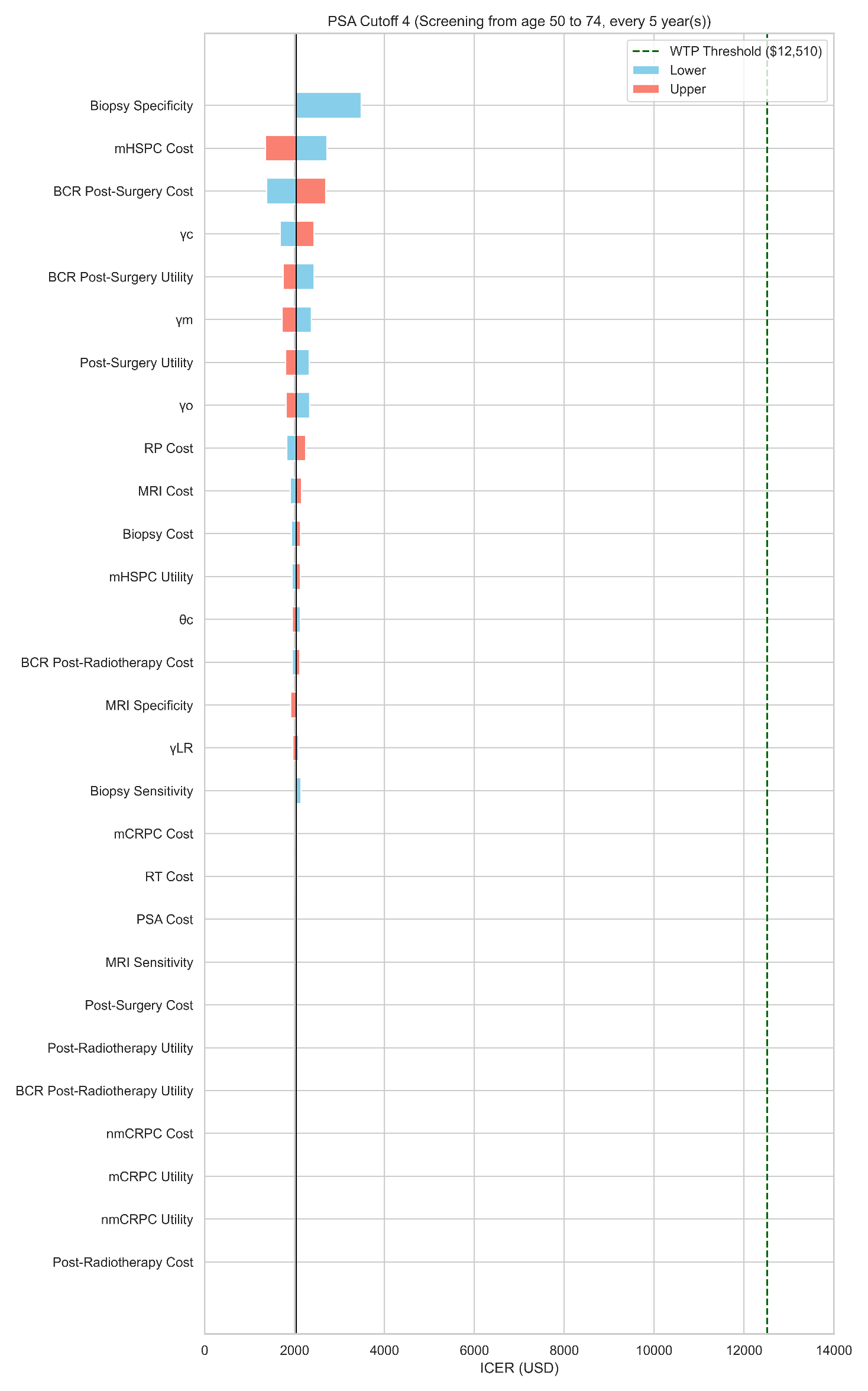


## PSA Cutoff 4, Age 55-74, Interval 1 year


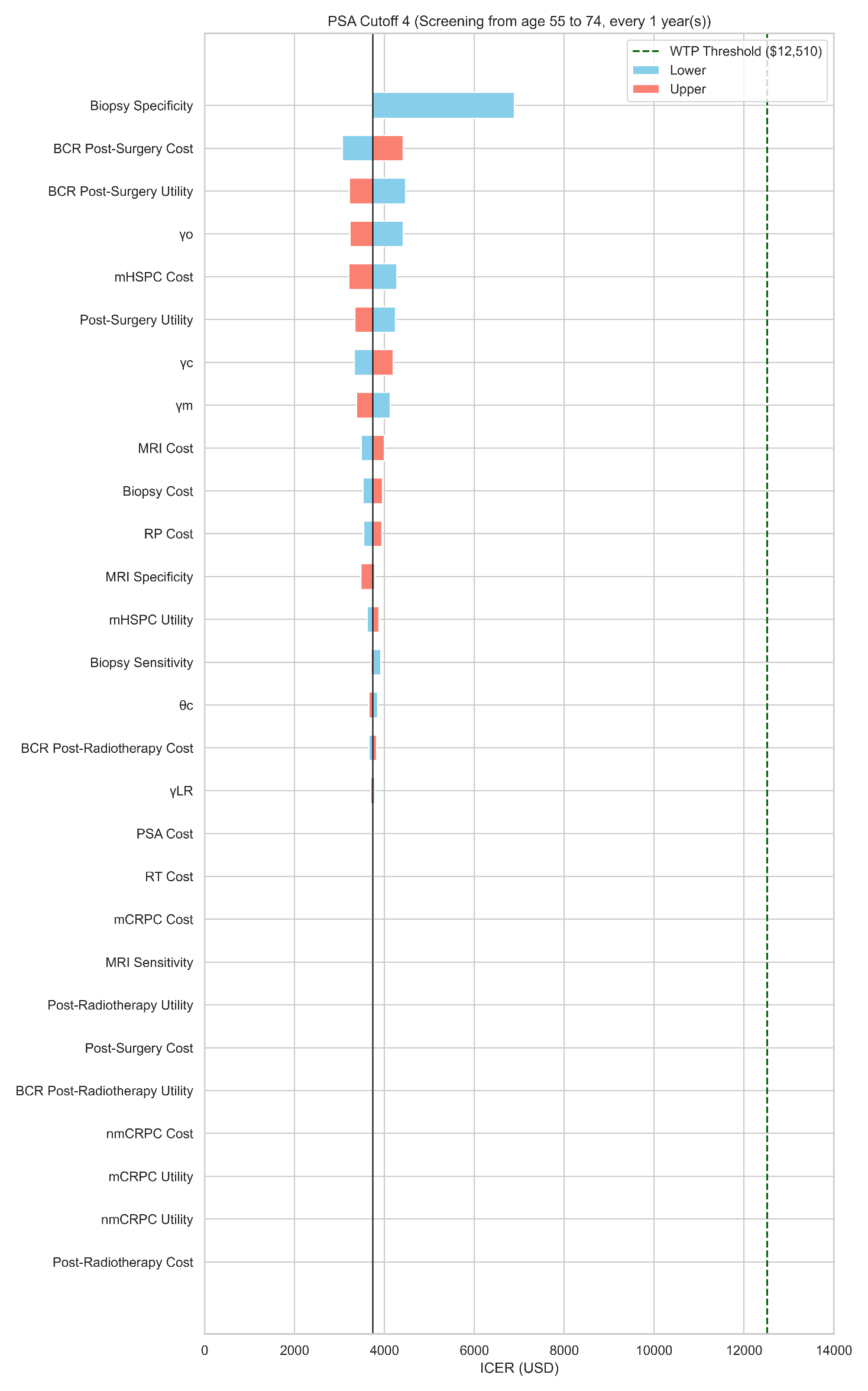


## PSA Cutoff 4, Age 55-74, Interval 2 years


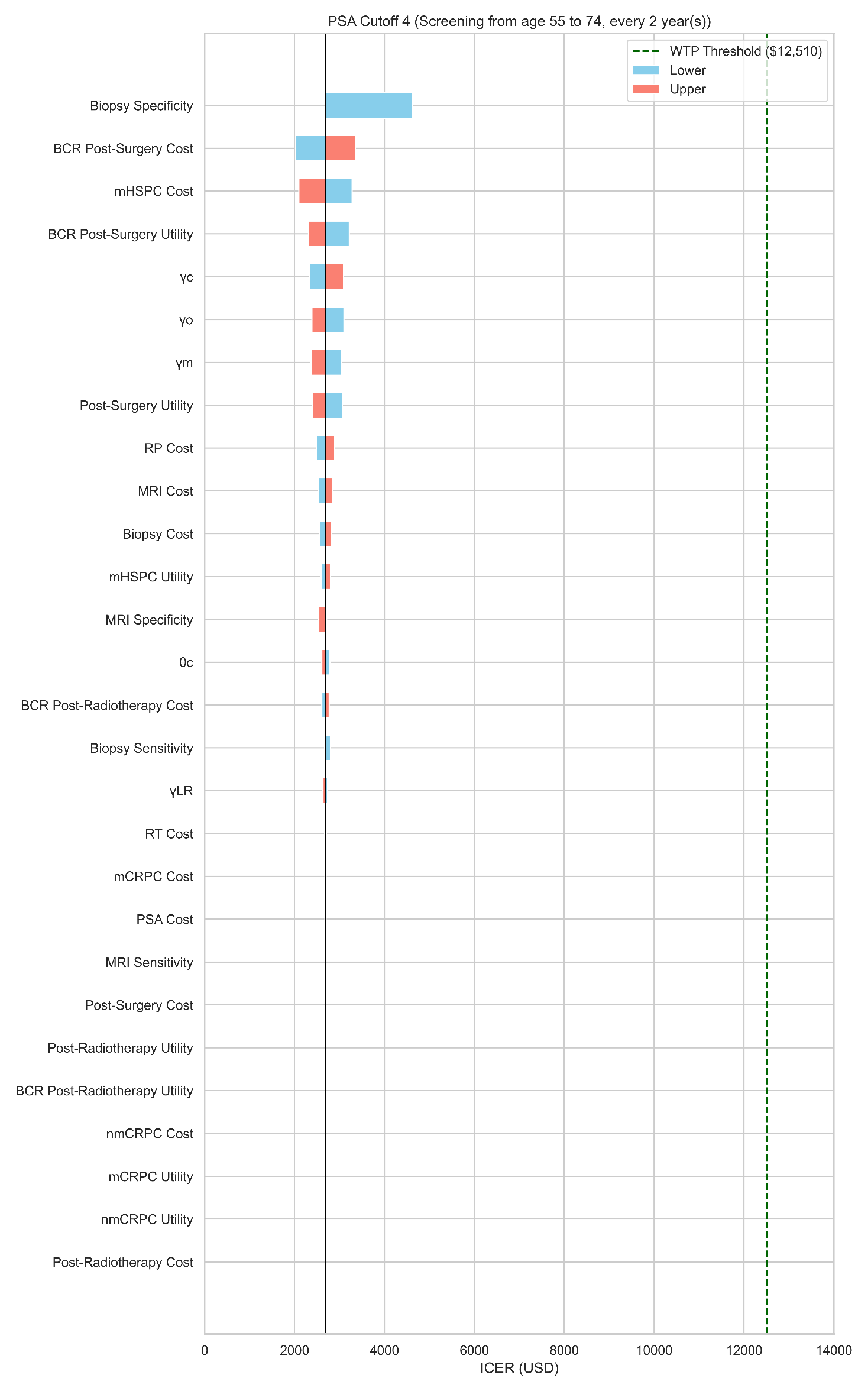


## PSA Cutoff 4, Age 55-74, Interval 3 years


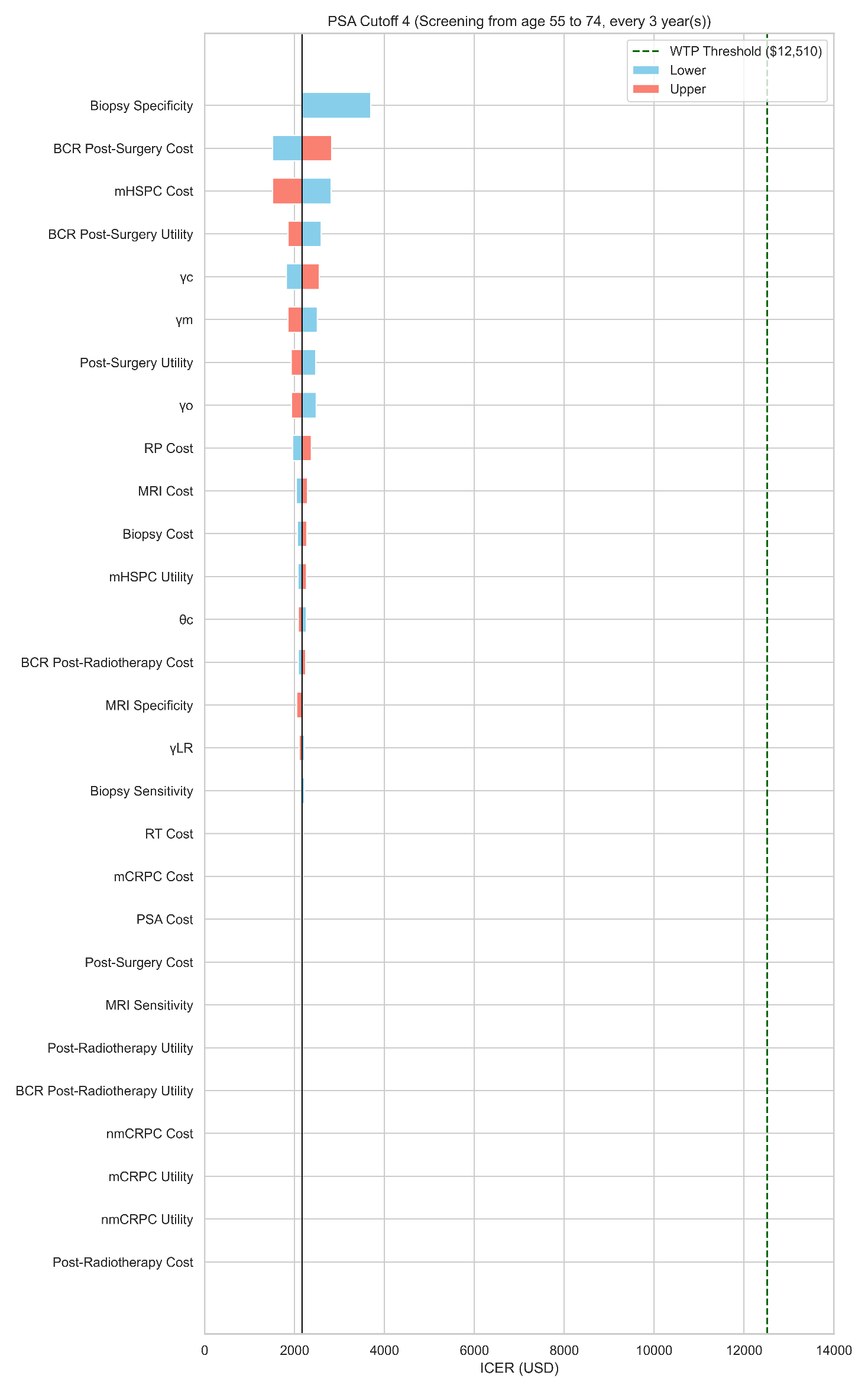


## PSA Cutoff 4, Age 55-74, Interval 5 years


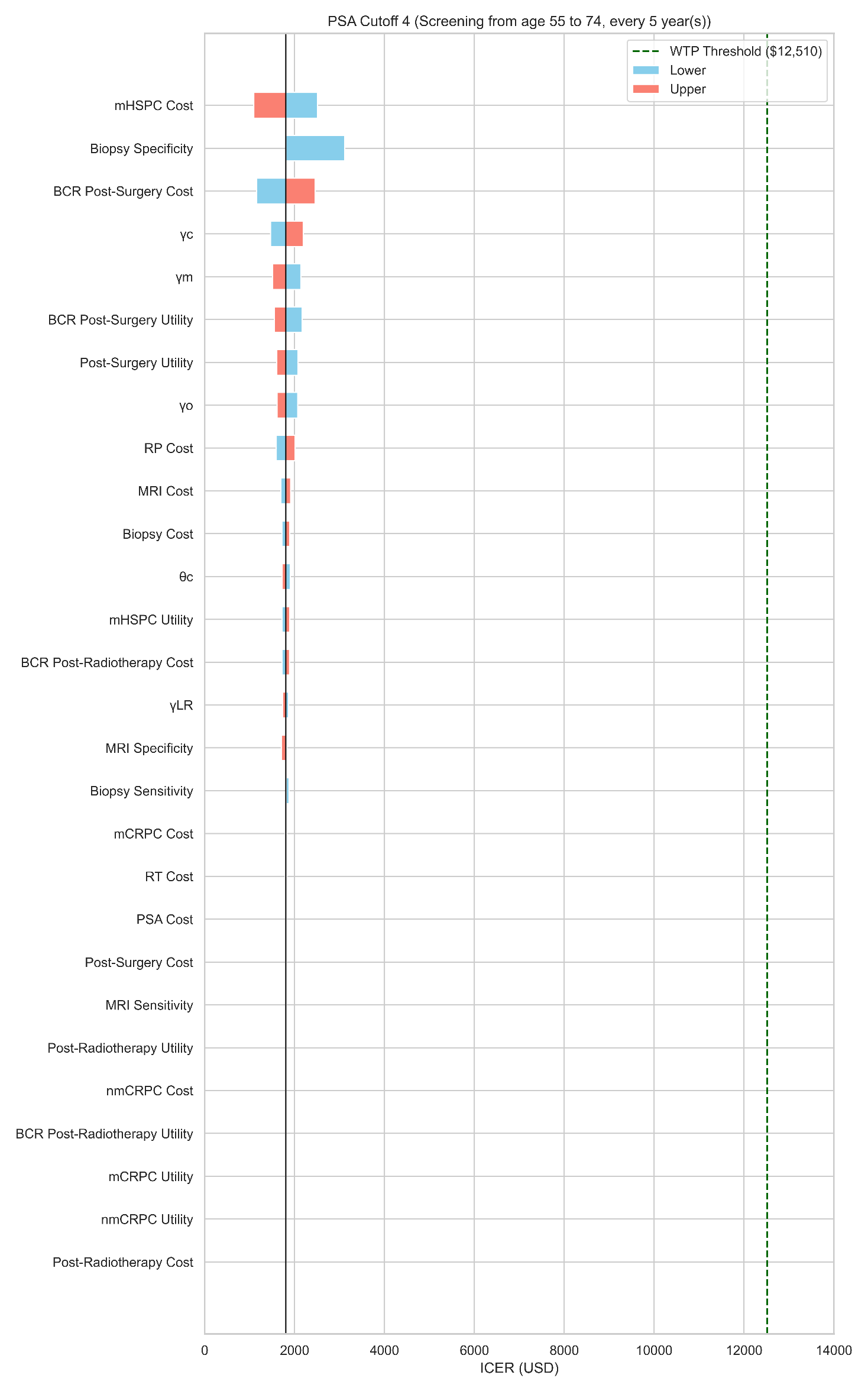


## PSA Cutoff 4, Age 60-74, Interval 1 year


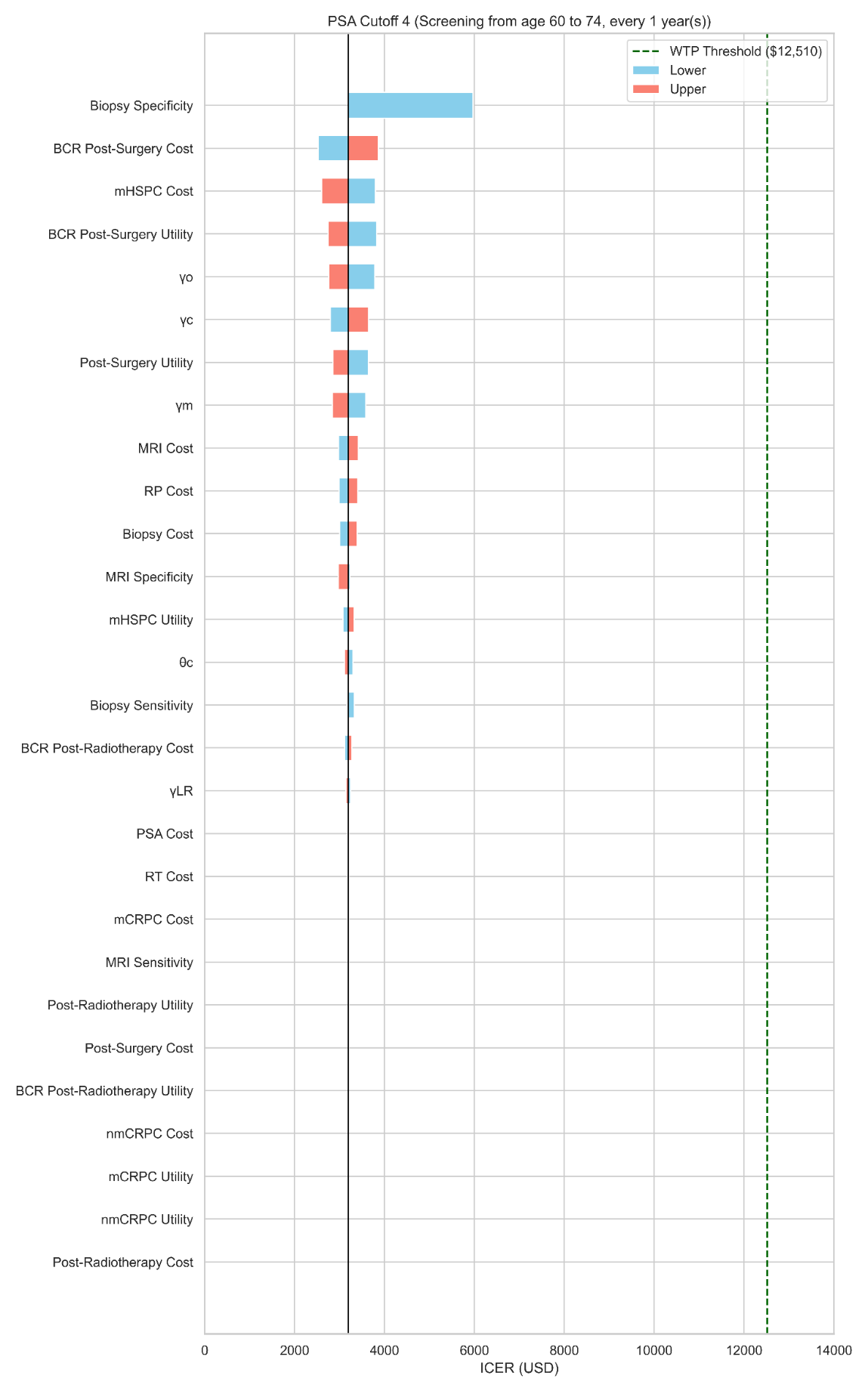


## PSA Cutoff 4, Age 60-74, Interval 2 years


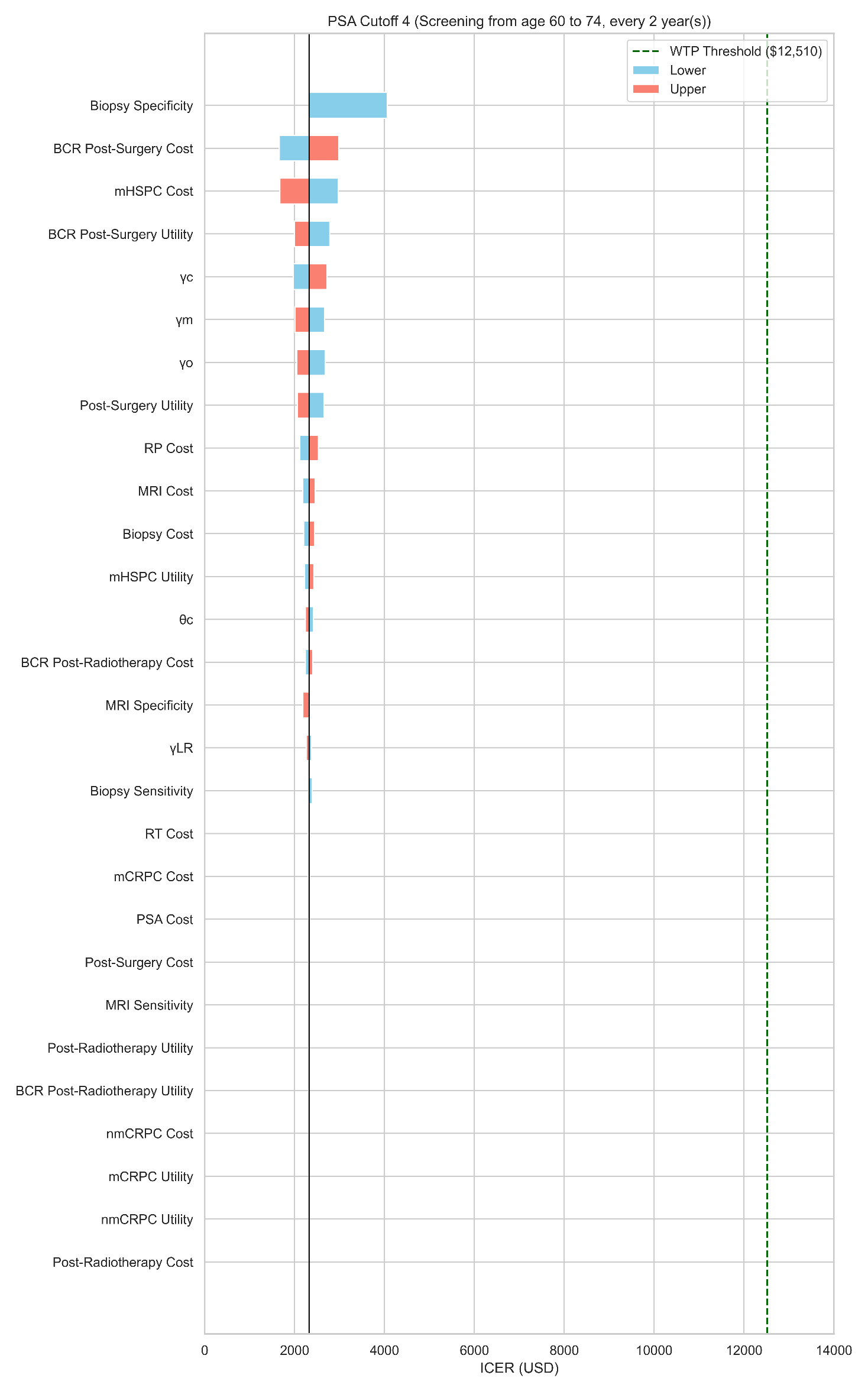


## PSA Cutoff 4, Age 60-74, Interval 3 years


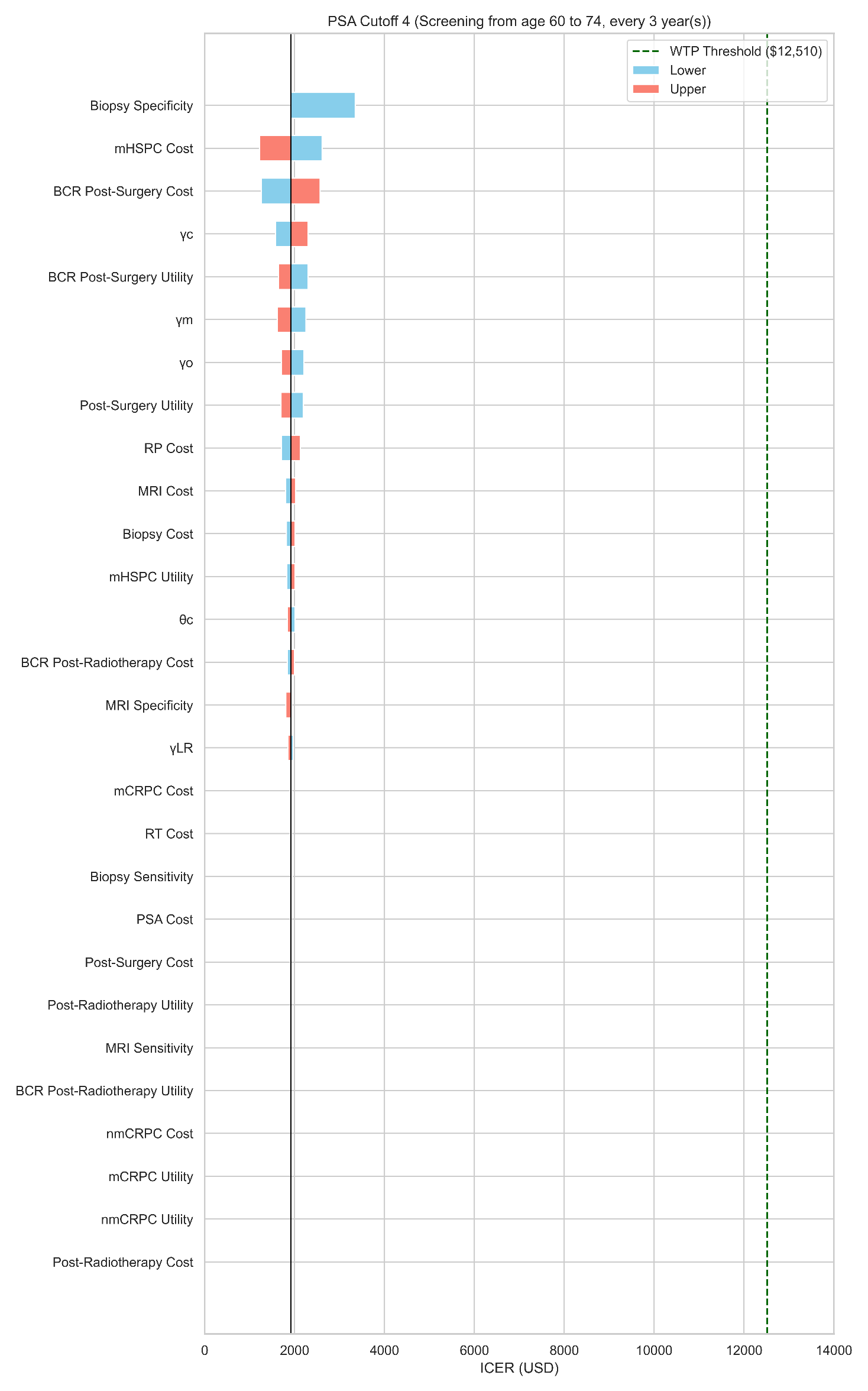


## PSA Cutoff 4, Age 60-74, Interval 5 years


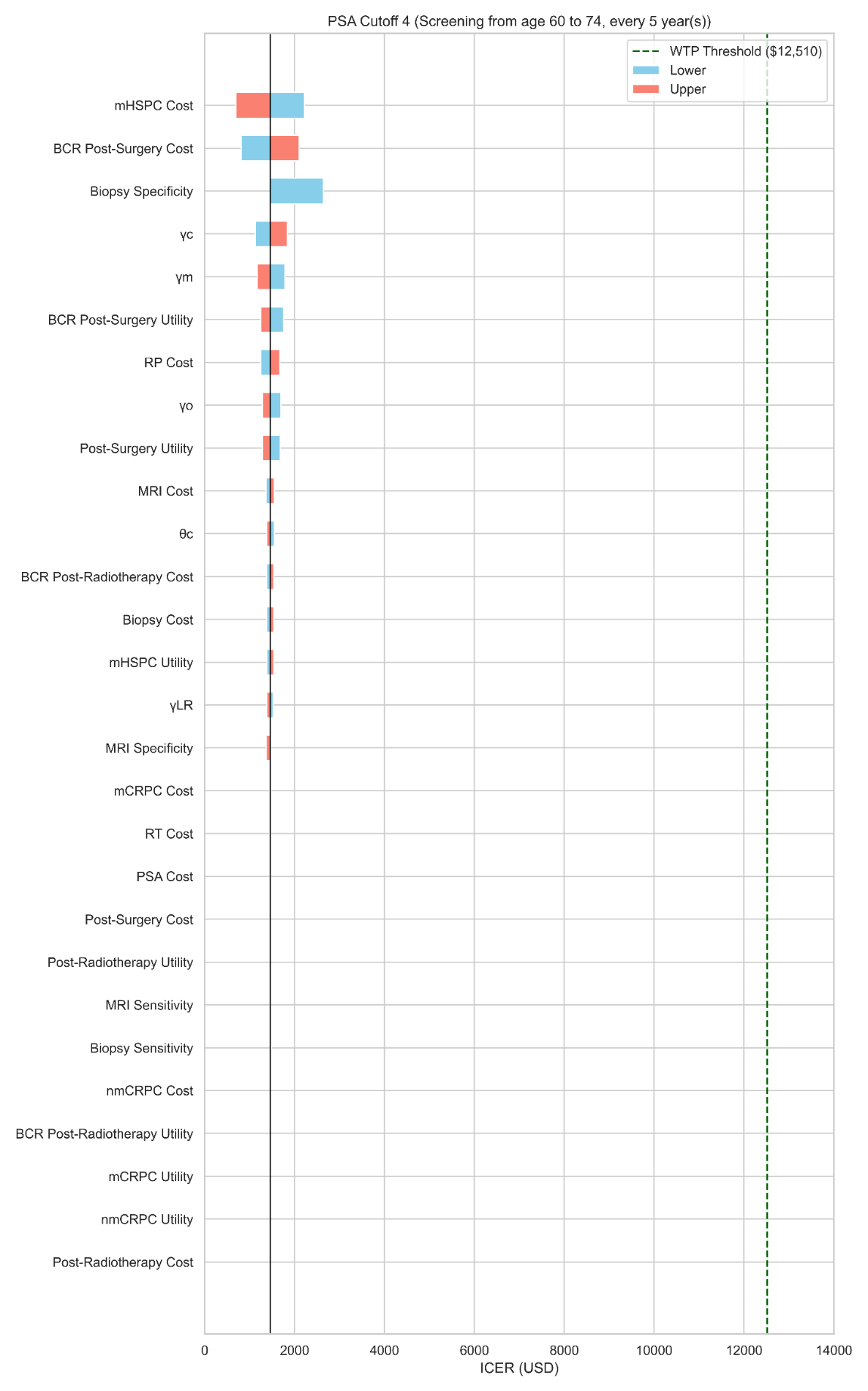


## Age-specific PSA Cutoff, Age 45-74, Interval 1 year


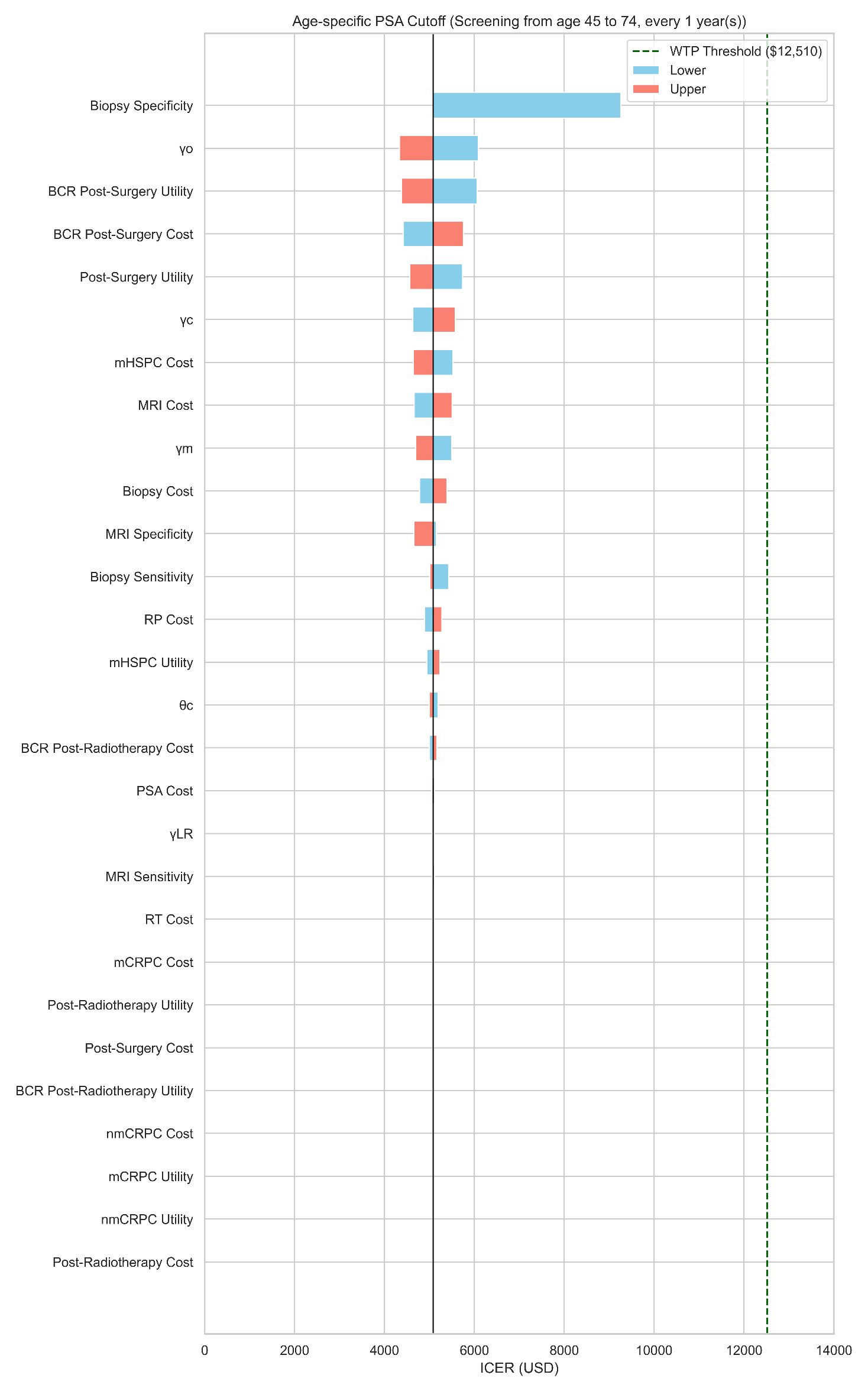


## Age-specific PSA Cutoff, Age 45-74, Interval 2 years


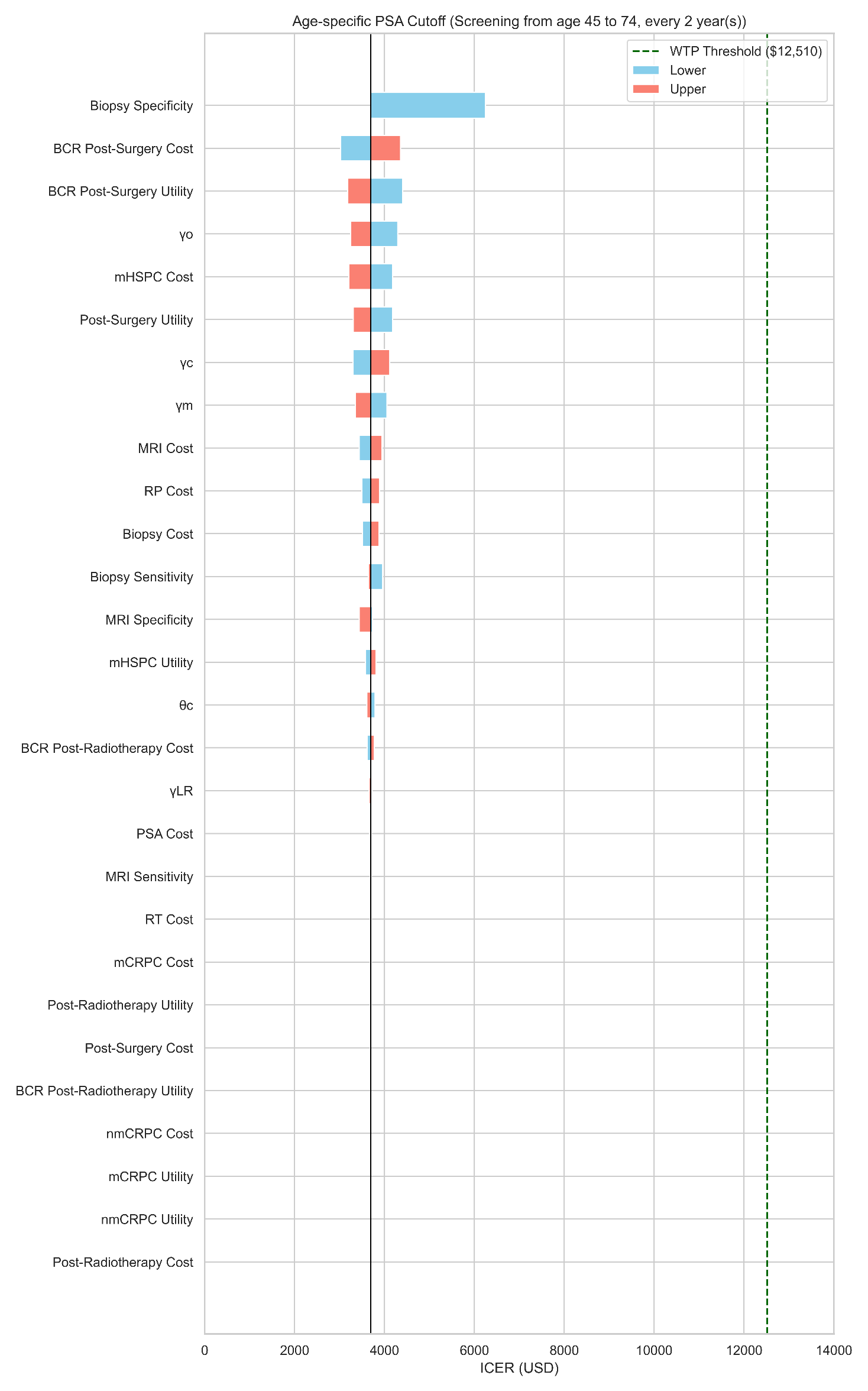


## Age-specific PSA Cutoff, Age 45-74, Interval 3 years


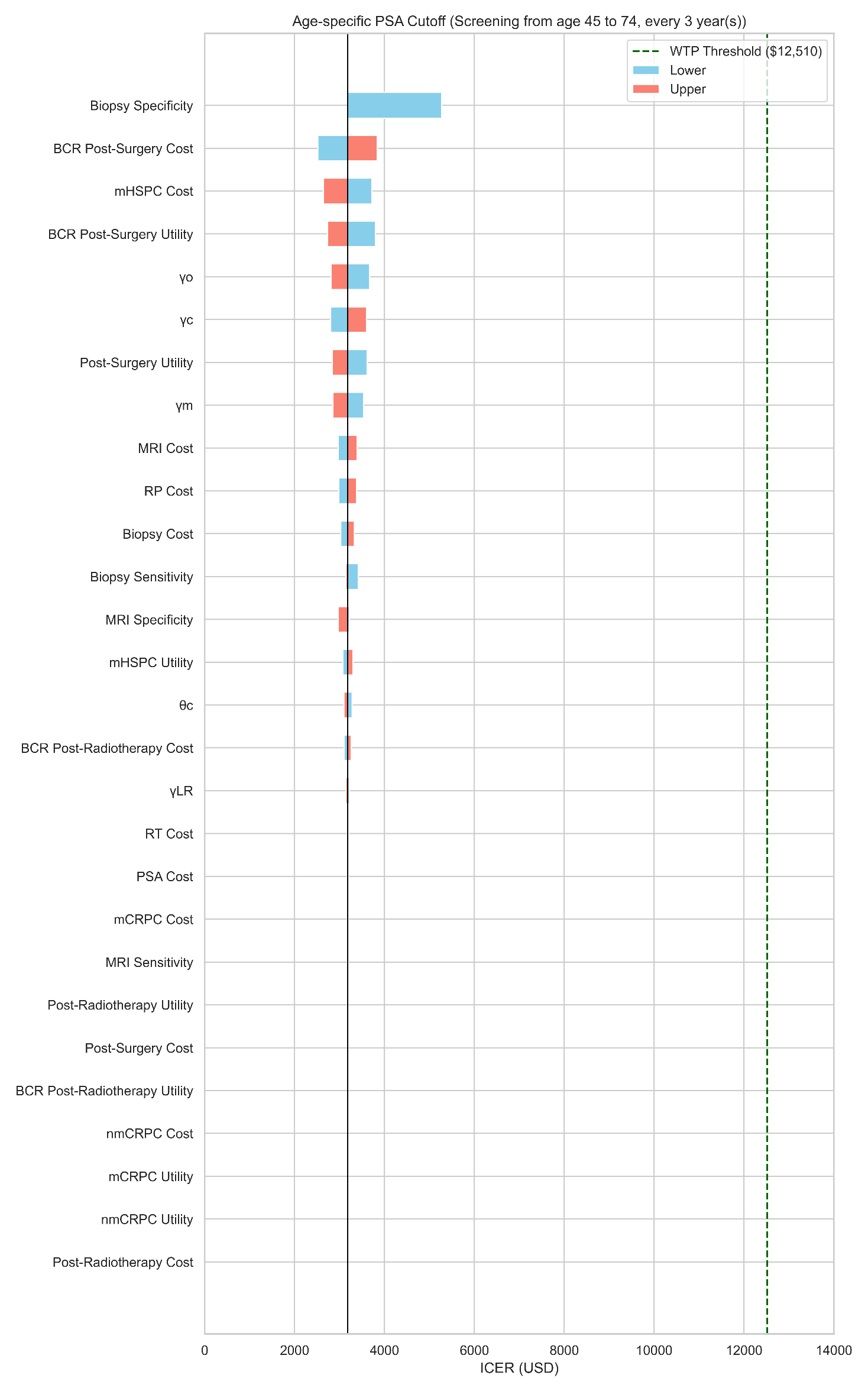


## Age-specific PSA Cutoff, Age 45-74, Interval 5 years


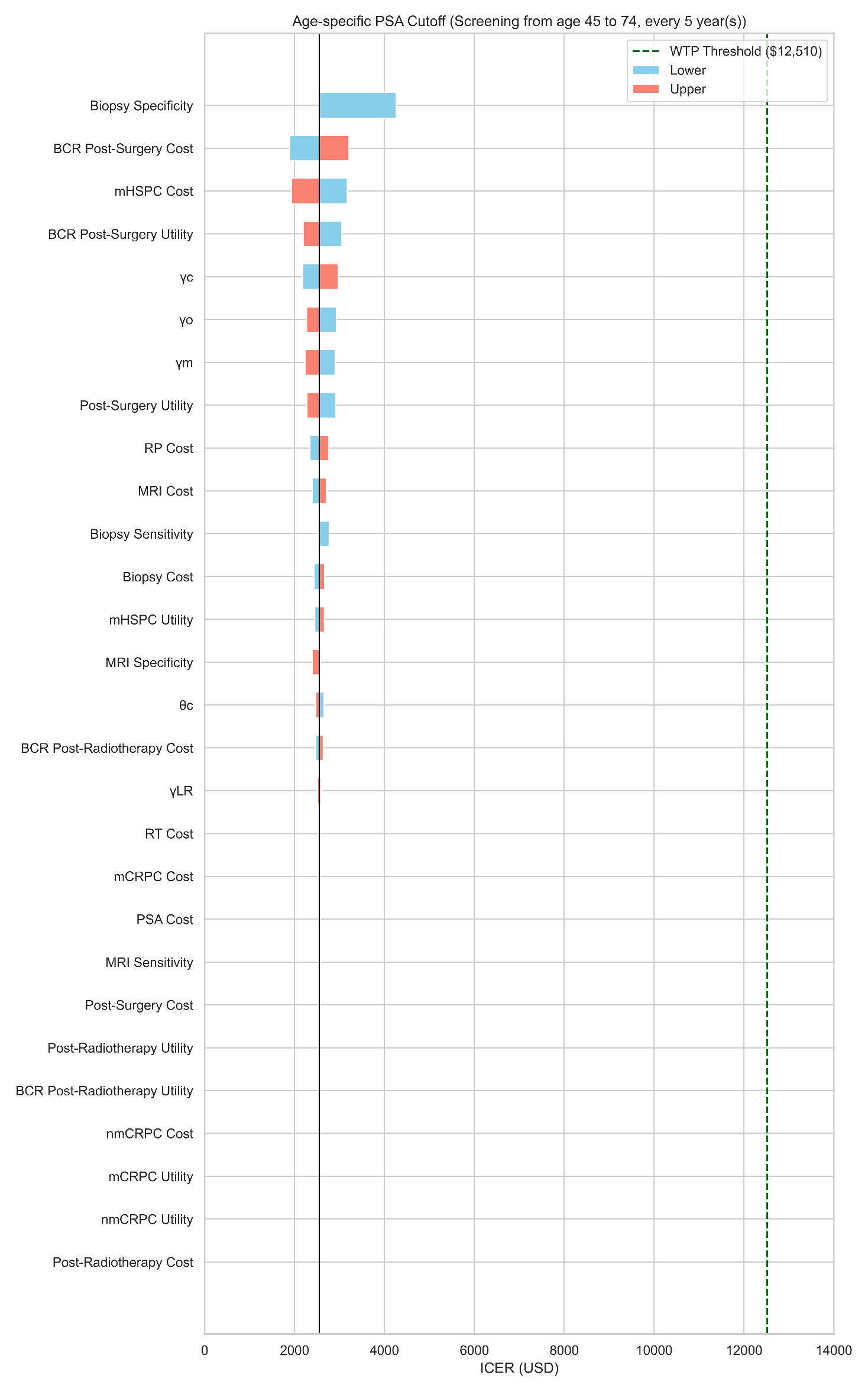


## Age-specific PSA Cutoff, Age 50-74, Interval 1 year


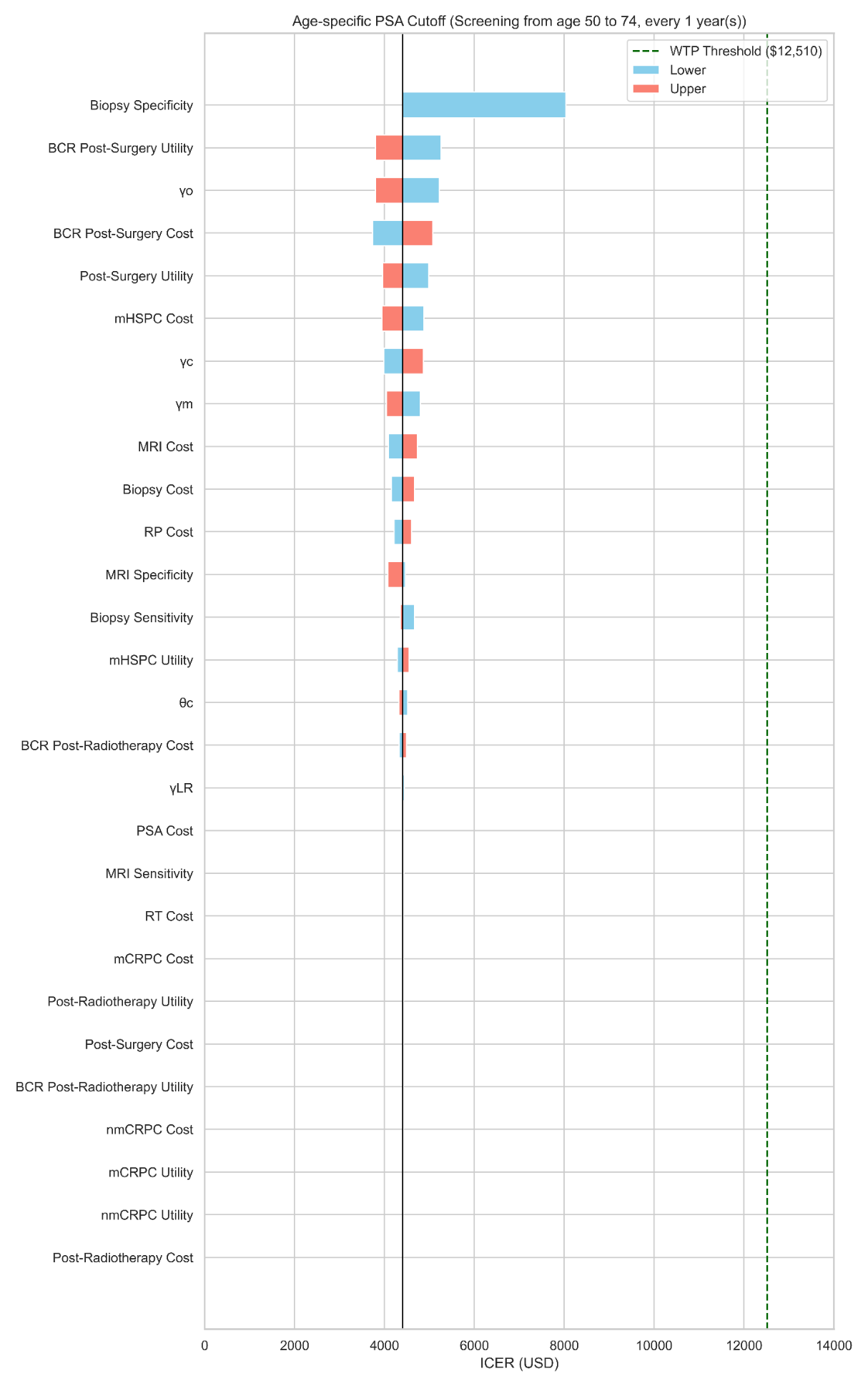


## Age-specific PSA Cutoff, Age 50-74, Interval 2 years


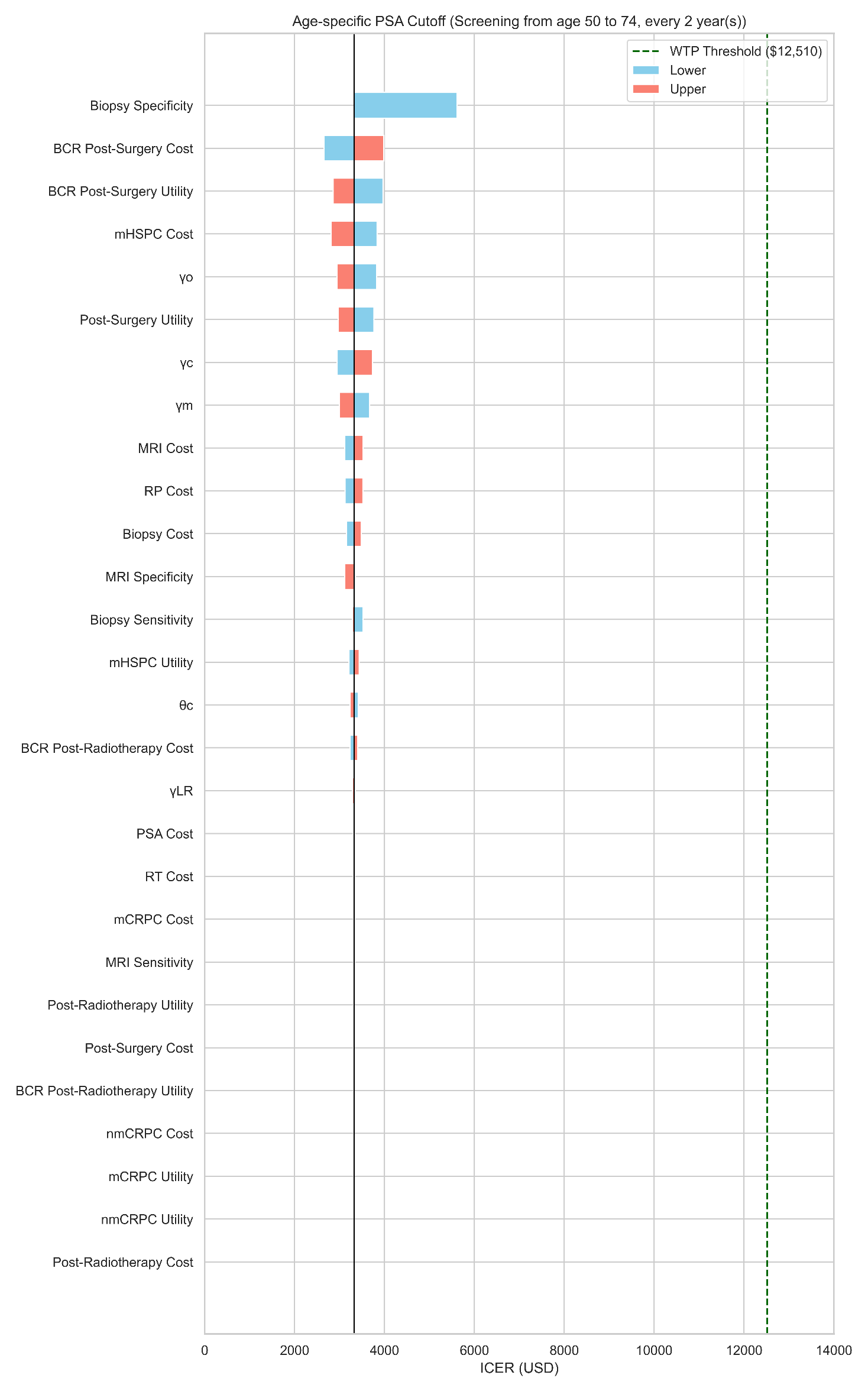


## Age-specific PSA Cutoff, Age 50-74, Interval 3 years


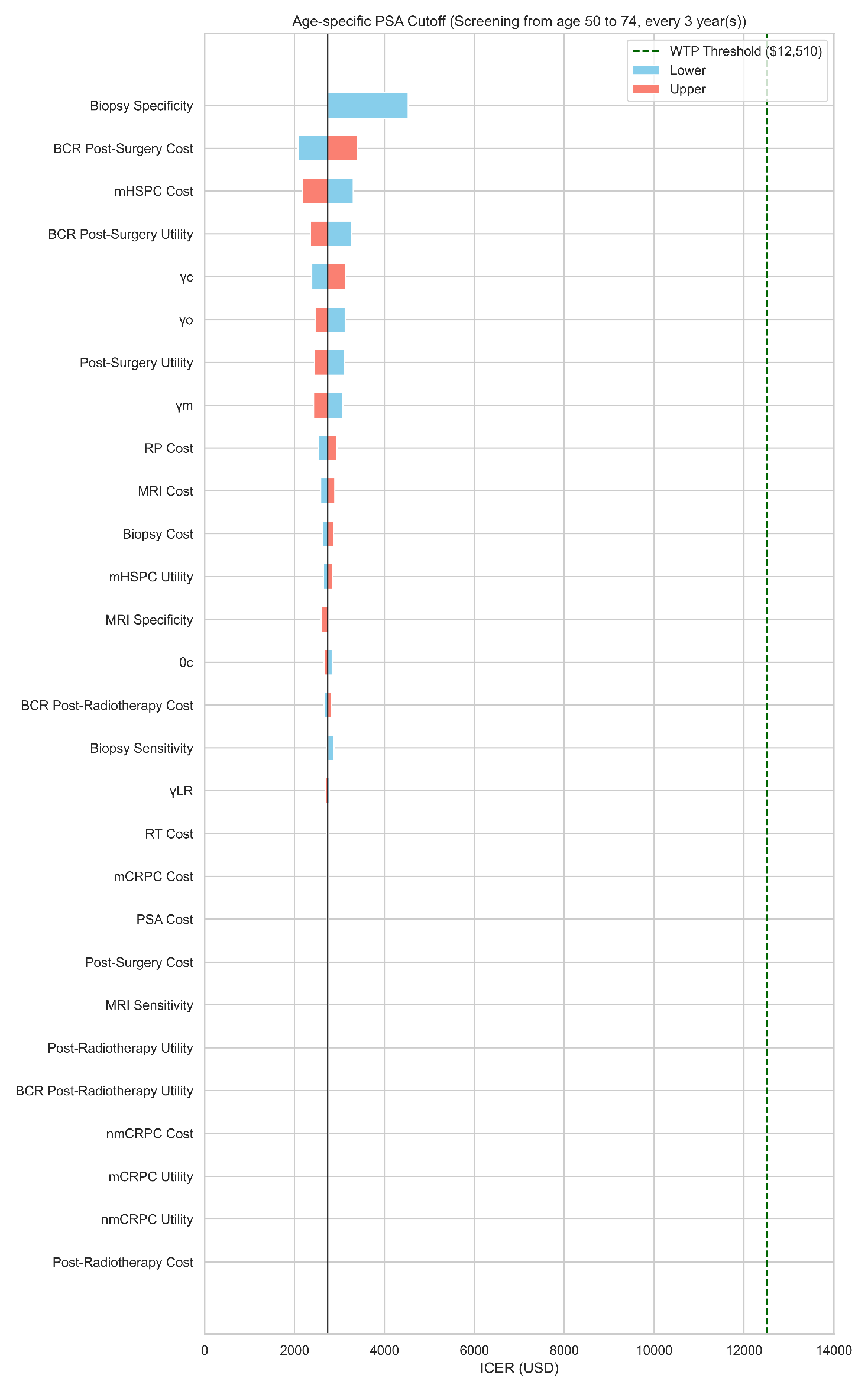


## Age-specific PSA Cutoff, Age 50-74, Interval 5 years


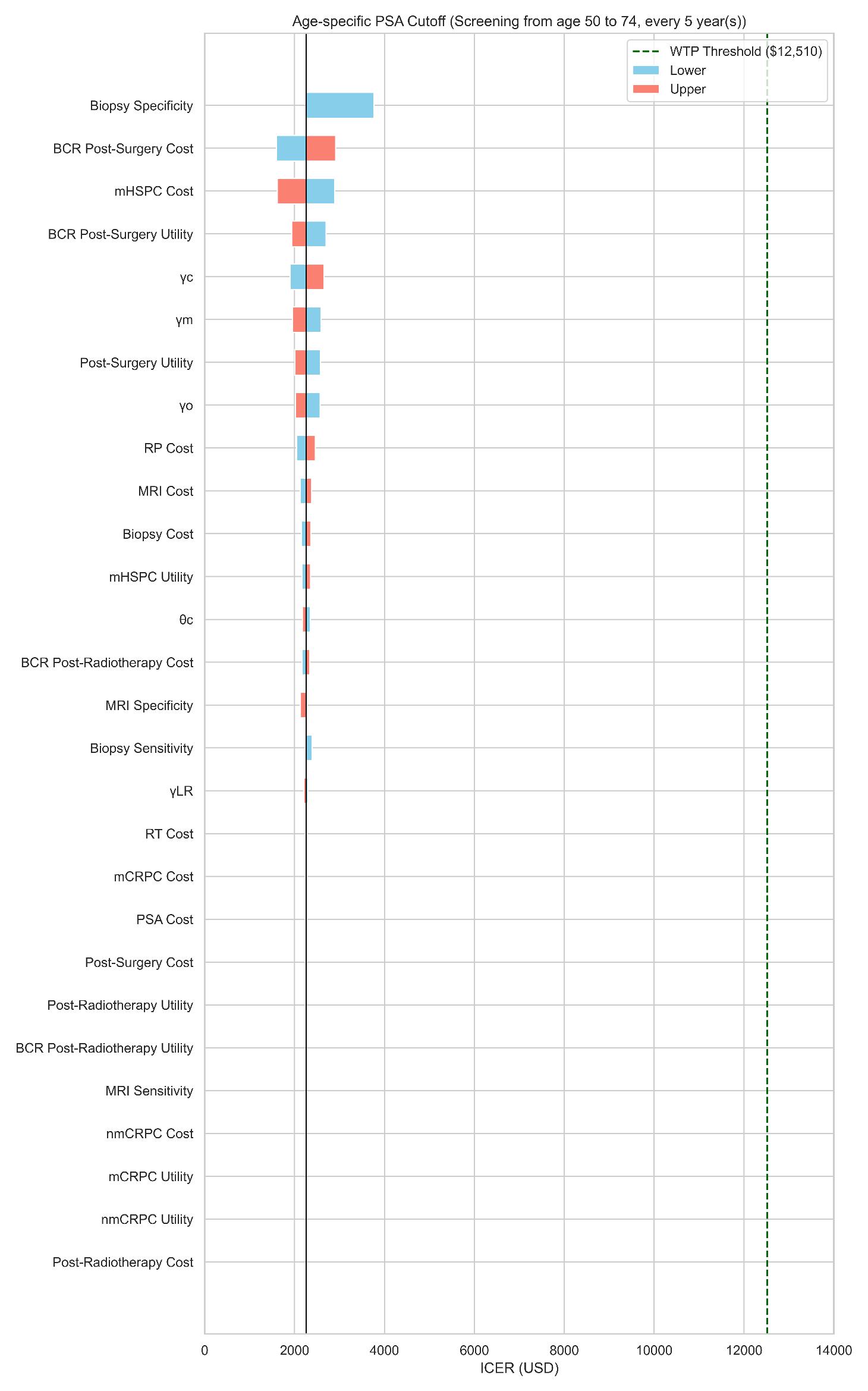


## Age-specific PSA Cutoff, Age 55-74, Interval 1 year


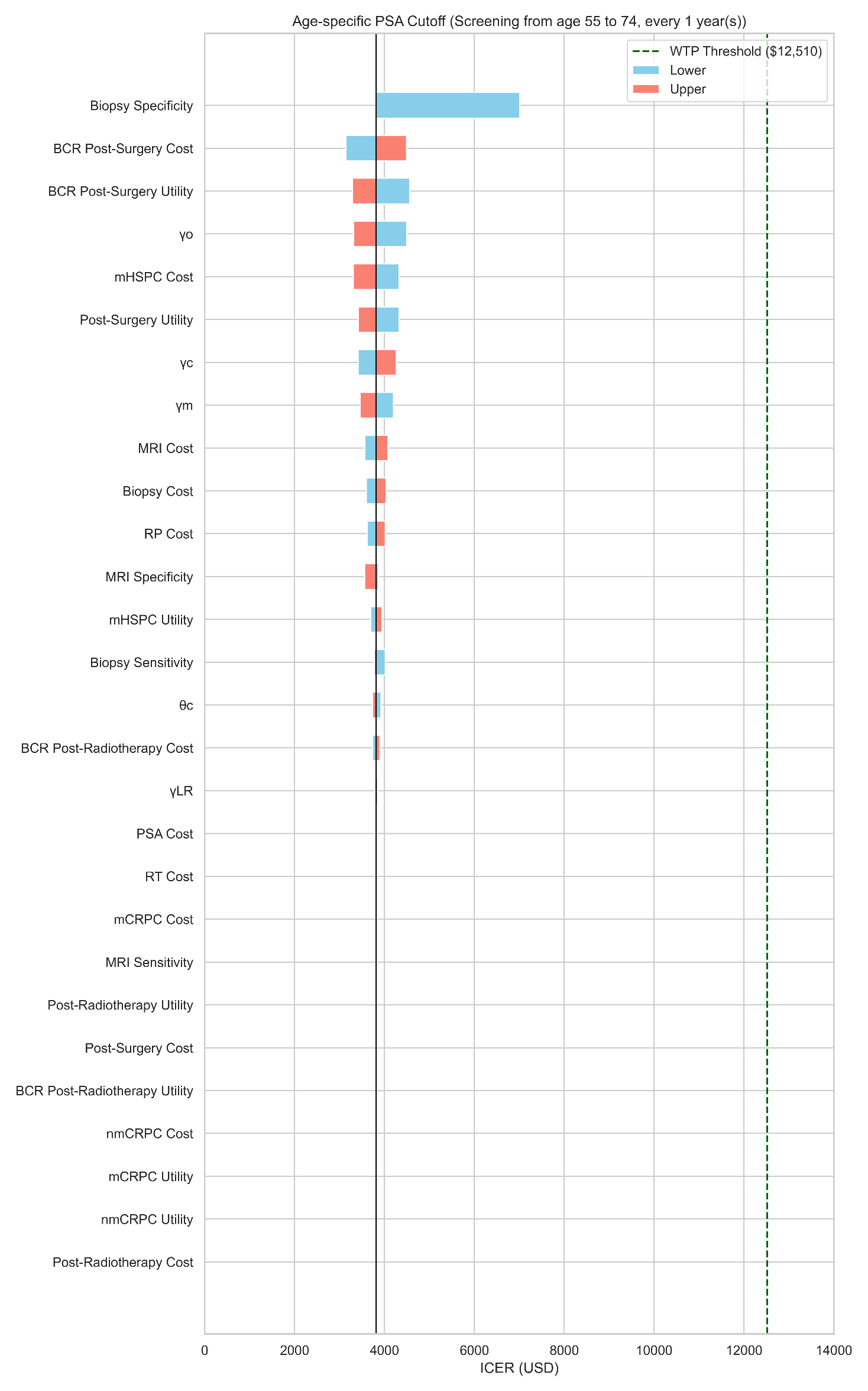


## Age-specific PSA Cutoff, Age 55-74, Interval 2 years


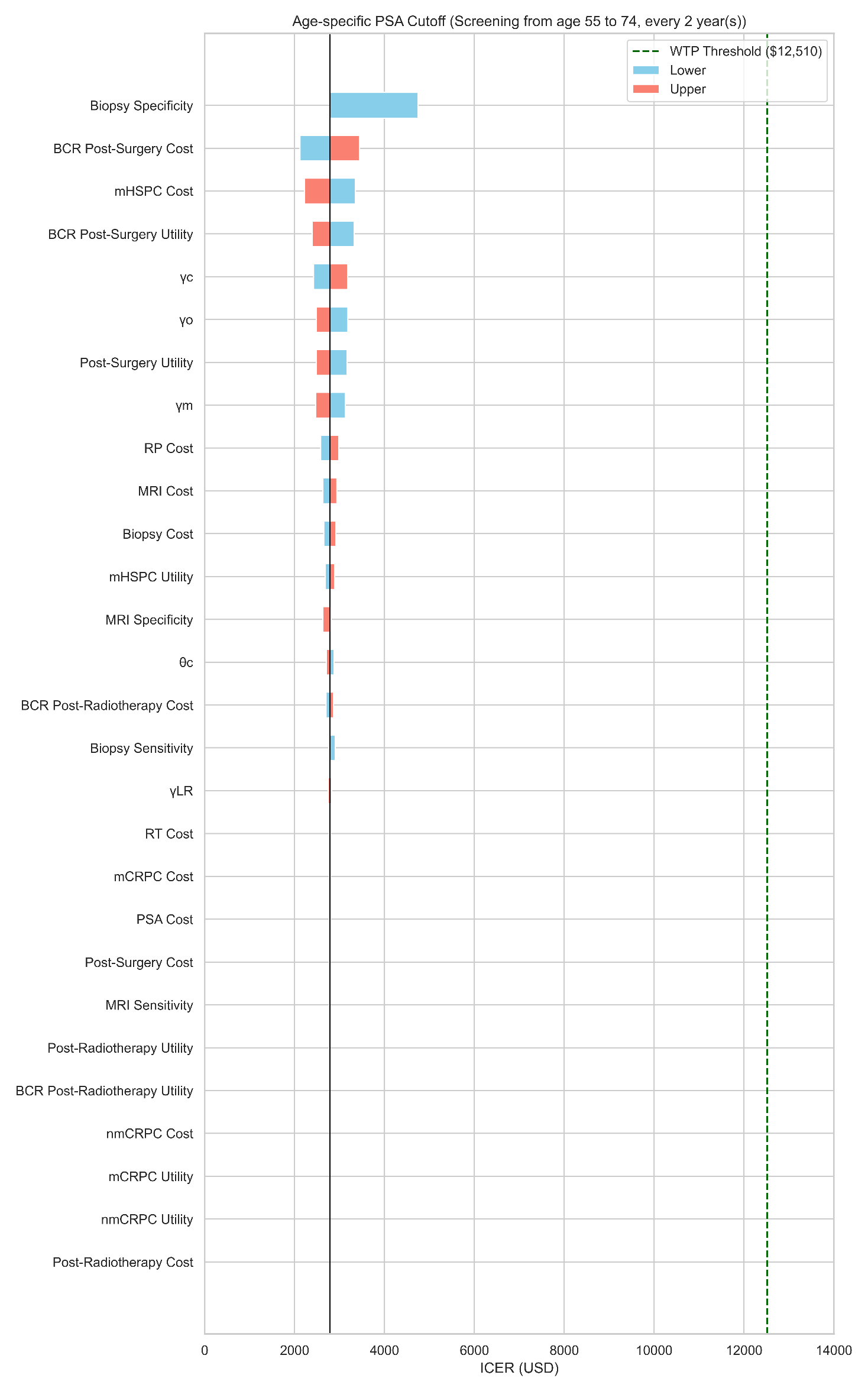


## Age-specific PSA Cutoff, Age 55-74, Interval 3 years


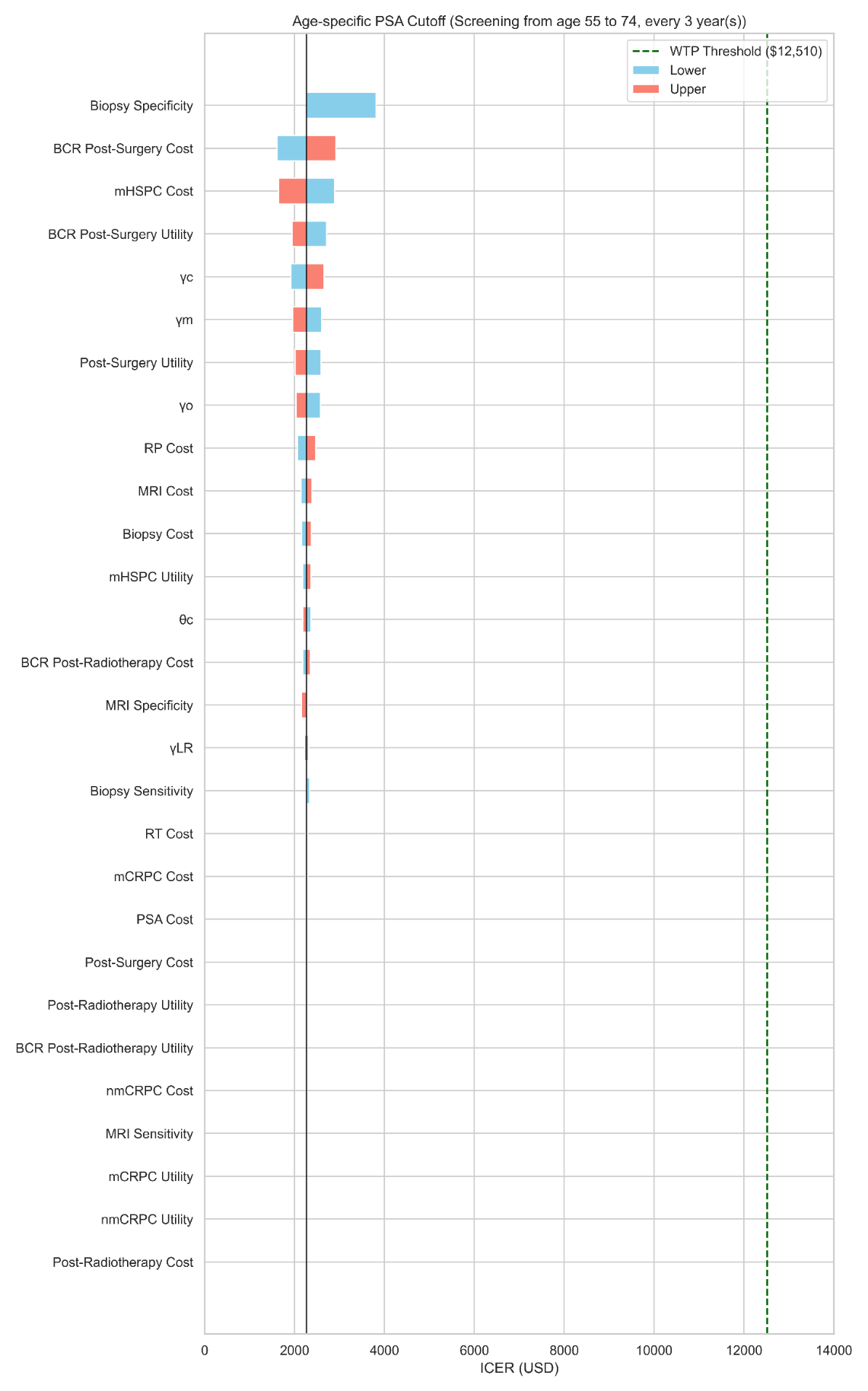


## Age-specific PSA Cutoff, Age 55-74, Interval 5 years


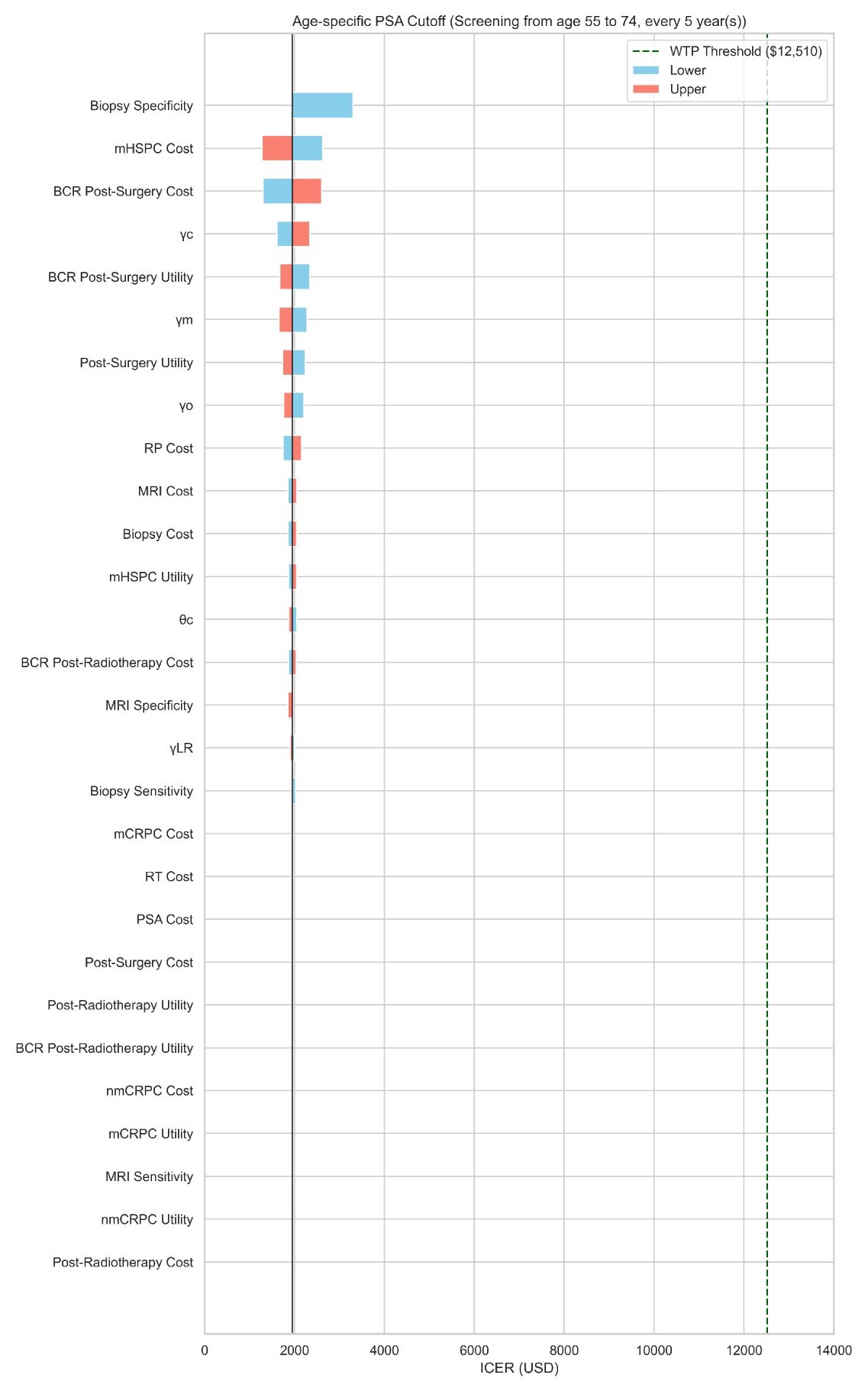


## Age-specific PSA Cutoff, Age 60-74, Interval 1 year


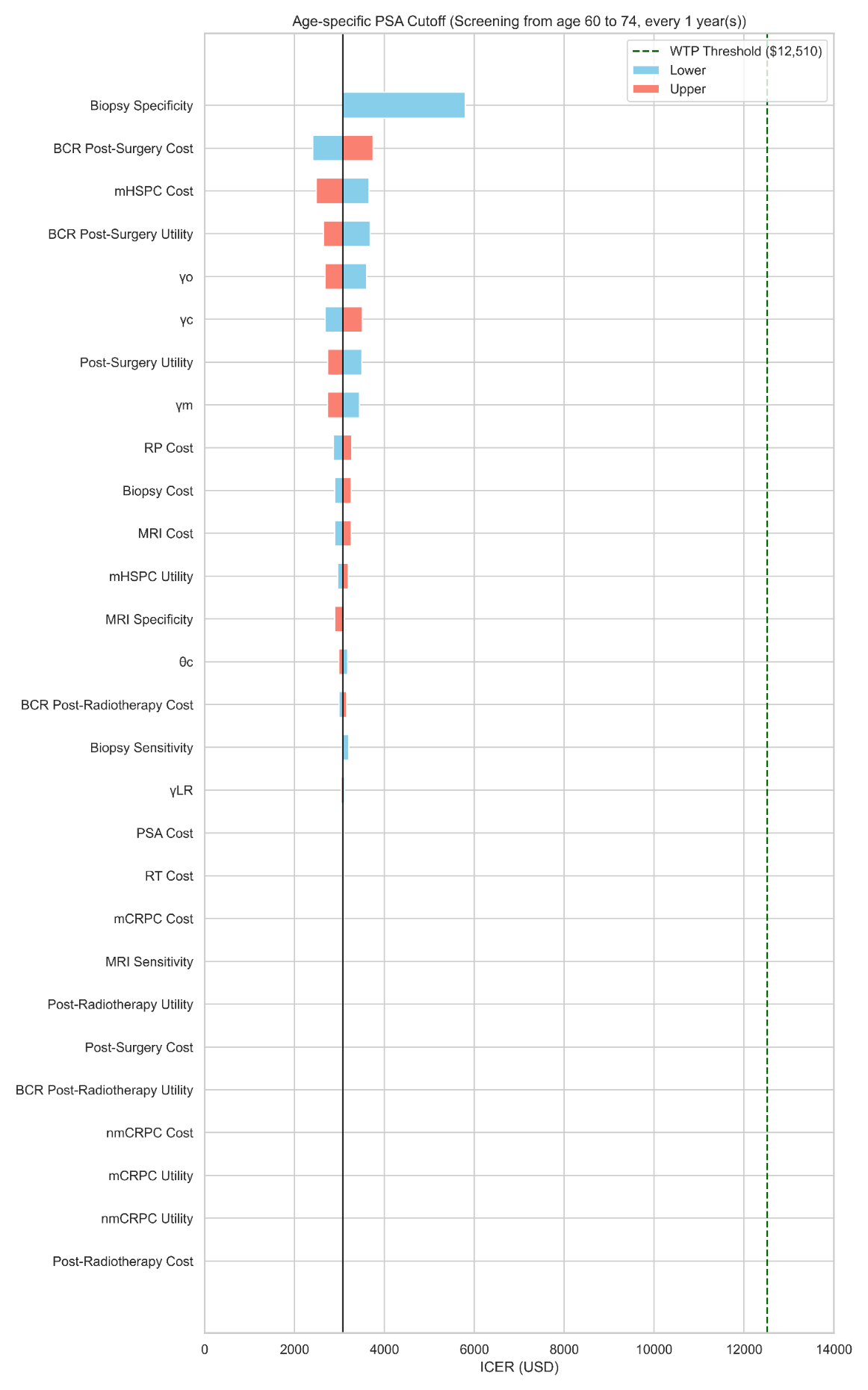


## Age-specific PSA Cutoff, Age 60-74, Interval 2 years


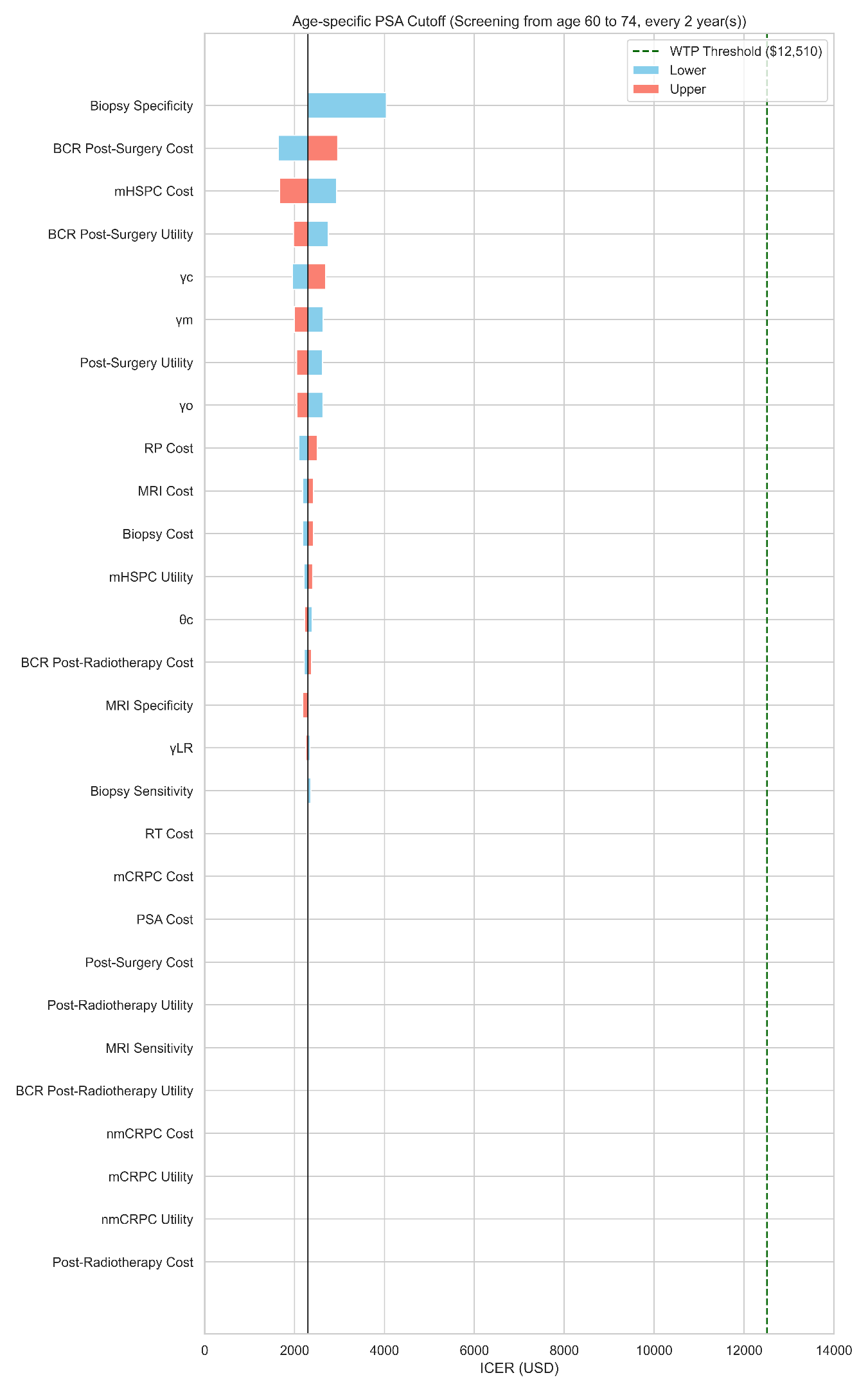


## Age-specific PSA Cutoff, Age 60-74, Interval 3 years


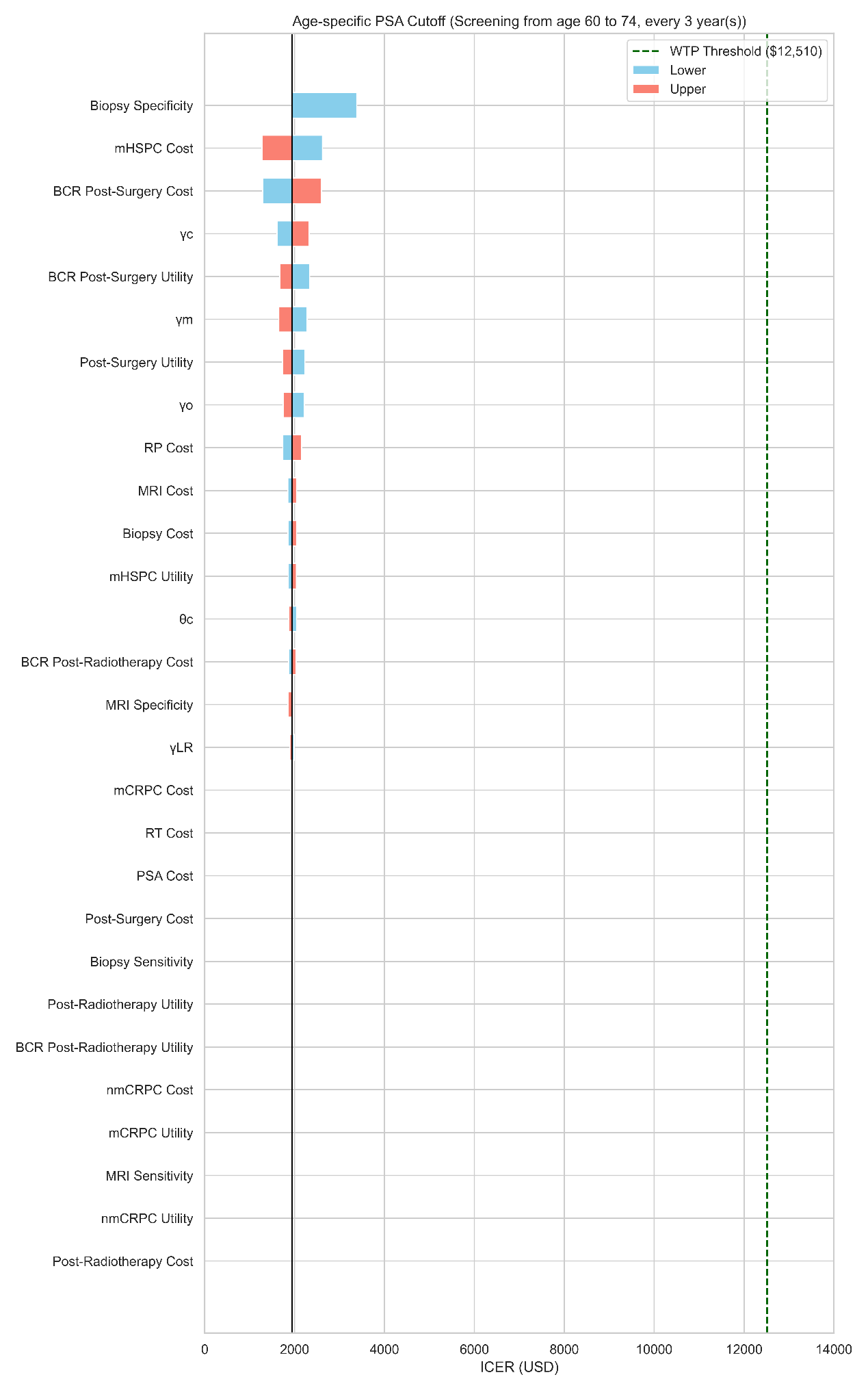


## Age-specific PSA Cutoff, Age 60-74, Interval 5 years


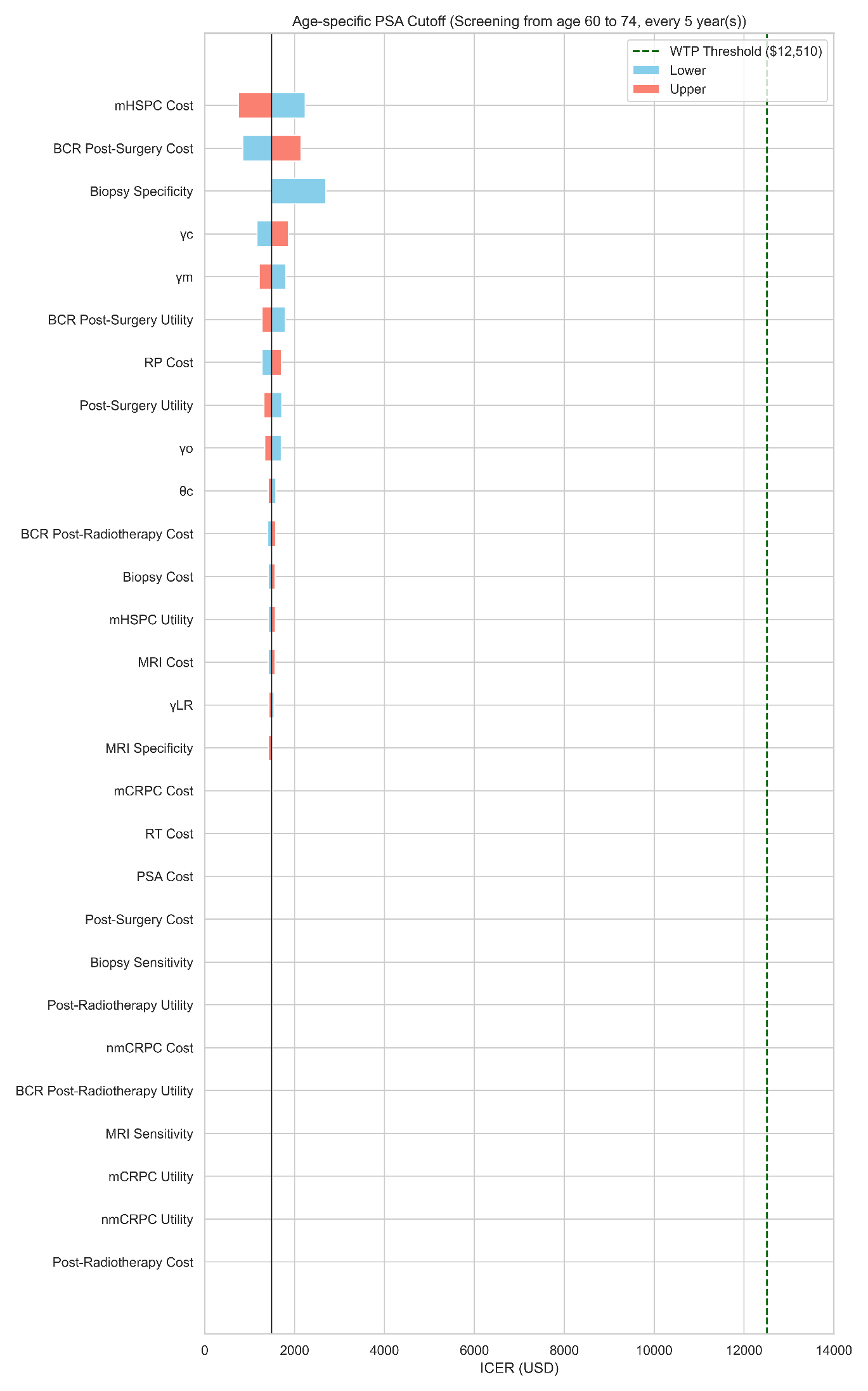


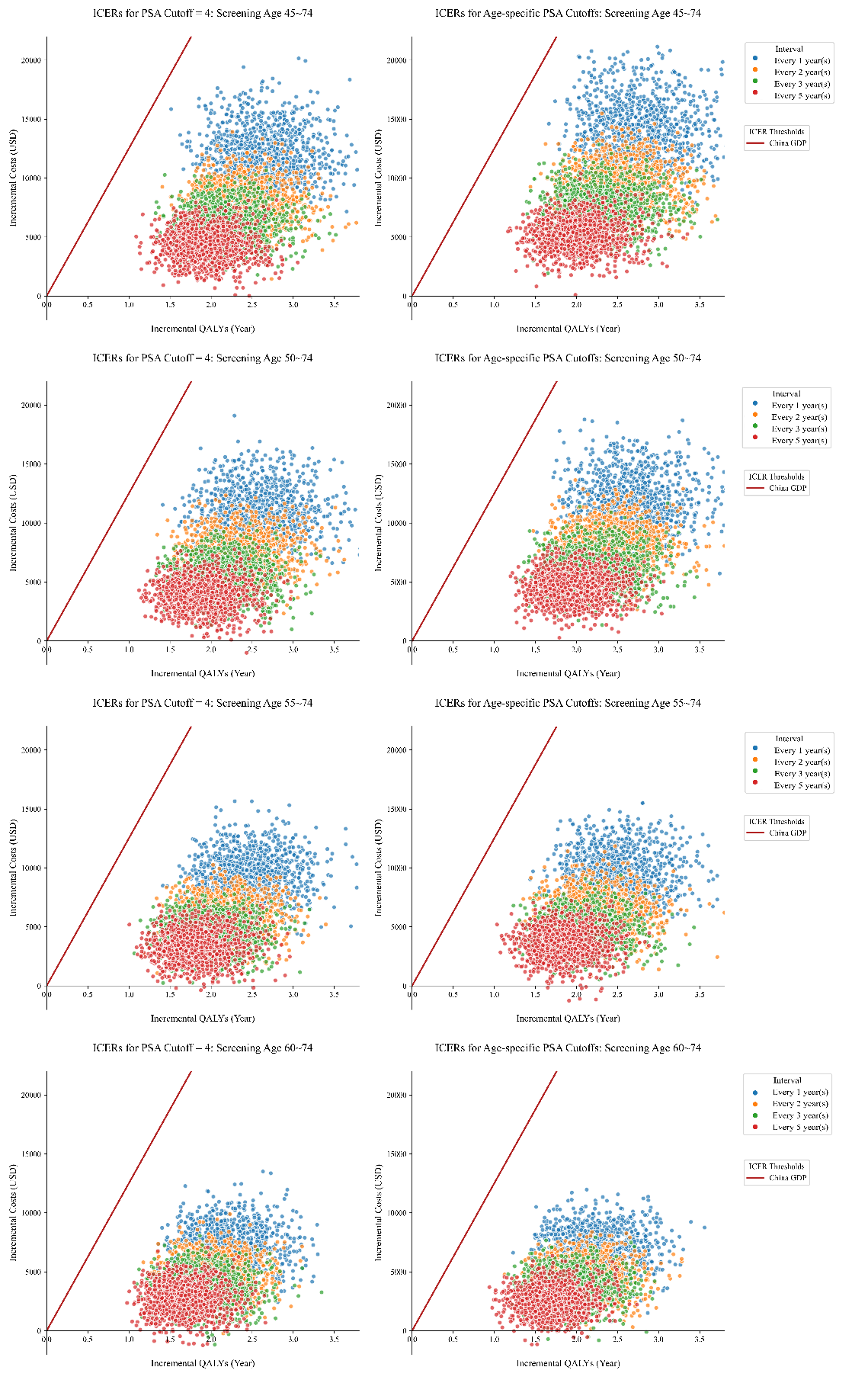


# Supplementary Figure 5. Probabilistic-Sensitivity Analysis

This Figure shows the results of probabilistic sensitivity analysis in different strategies. Panels on the left side (A, B, C, D) represent the results of strategies under the PSA Cutoff = 4 while those on the right side (E, F, G, H) represent the results of strategies under the Age specific PSA cutoffs. Each coloured point represents one simulation draw for a specific screening strategy, defined by screening ages (45-74, 50-74, 55-74 and 60-74) and intervals (1, 2, 3, or 5 years). The solid red line indicates a willingness‐to‐pay threshold of 1× China’s per capita GDP. Strategies falling below or to the right of the line indicate that they are cost-effective under the threshold.


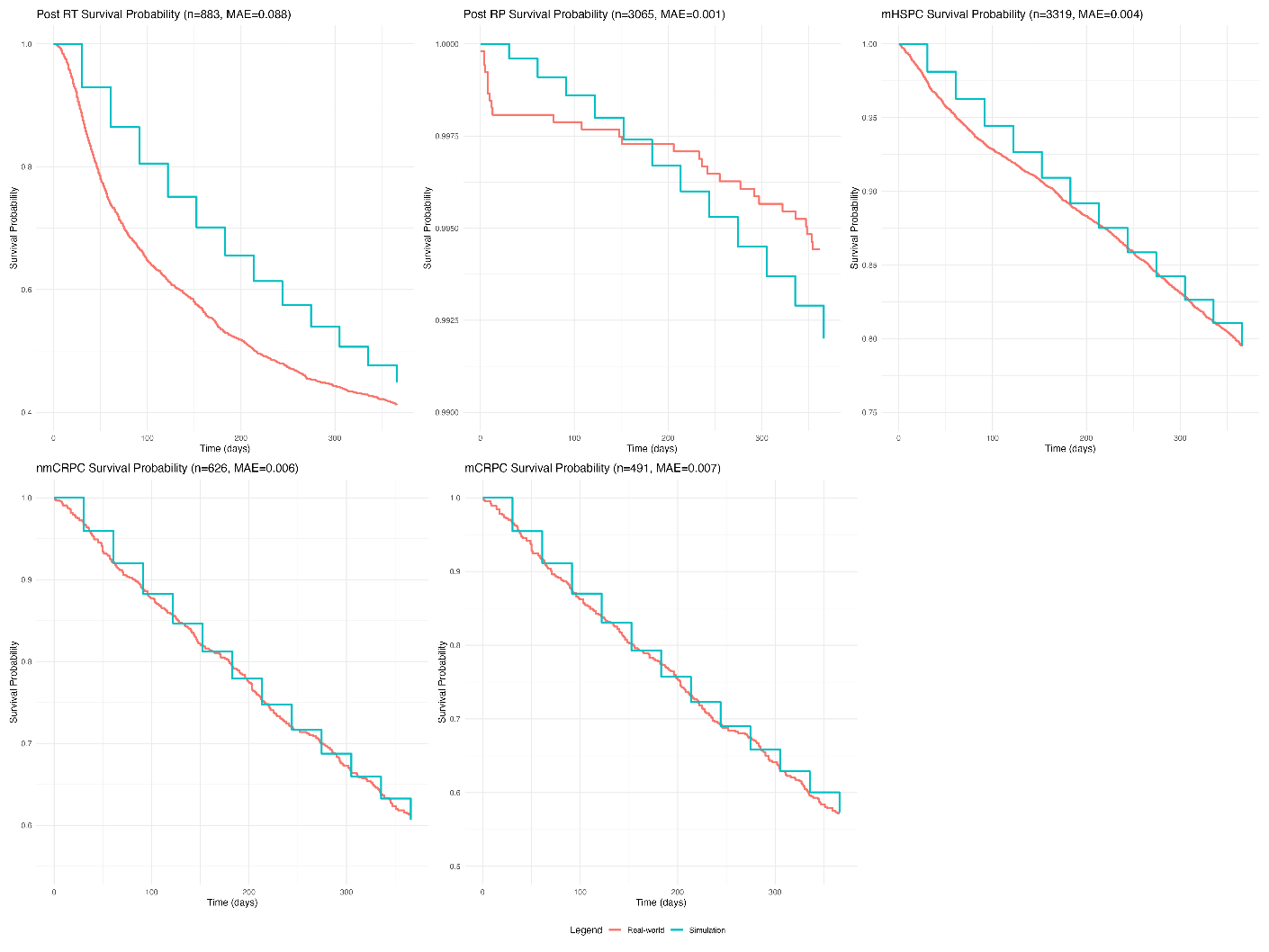


# Supplementary Figure 6. Goodness-of-Fit for the Post-Diagnosis

In the figure, blue lines represent the model's simulated survival curves, and red lines represent the actual survival curves observed in your real-world data. The MAE in each title quantifies the average difference between the simulated and real-world curves.

# Supplementary Table 1. Multipliers for Tumour Onset Hazard among High-Risk Populations

| **Risk Factors** | **Multiplier** | **Proportion** | **Reference** |
| --- | --- | --- | --- |
| **RPMs** | 2·83 | 1·6% | ^17^ |
| **PRS (high)** |  | 25% | ^17^ |
| **low vs average PRS** | 0·57 | / | / |
| **high vs average PRS** | 1·97 | / | / |
| **Family History** | 1·8 | 8·2% | ^17^ |
| PCa, prostate cancer; PRS, polygenetic risk score; RPM, rare pathogenic mutation  RPMs of three genes: BRCA2, HOXB13, and CHEK2.  PRS was divided into three groups: bottom quartile for low GRS (25%), middle two quartiles for average GRS (50%), and top quartile for high GRS (25%, positive).  No interaction effect was found among these three inherited risk factors.^17^ | | | |

# Supplementary Table 2. Validation of the Fred Hutchinson Cancer Research Centre Prostate Cancer Incidence Model Against WHO Estimates in China

|  | **WHO-Reported Incidence (per 100,000)** | **Model Projected Prostate Cancer Incidence in China (per 100,000)** | | | | | | | |
| --- | --- | --- | --- | --- | --- | --- | --- | --- | --- |
| multiplier | / | 1 | 2·4 | 2·6 | 2·8 | **3**^γ^ | 3·2 | 3·4 | 3·6 |
| γo | / | 0·00008 | 0·000192 | 0·000208 | 0·000224 | **0·00024**^γ^ | 0·000256 | 0·000272 | 0·000288 |
| γc | / | 0·0015 | 0·000625 | 0·000577 | 0·000536 | **0·0005**^γ^ | 0·000469 | 0·000441 | 0·000417 |
| <40 | 0·00 ^α^ | 0·016 | 0·0161 | 0·0161 | 0·0161 | **0·0161 ^α^** | 0·016 | 0·0161 | 0·0161 |
| 40~44 | 0·37 ^α^ | 1·009 | 1·021 | 1·022 | 1·022 | **1·022 ^α^** | 1·023 | 1·023 | 1·024 |
| 45~49 | 1·4 ^α^ | 3·777 | 3·913 | 3·920 | 3·927 | **3·928 ^α^** | 3·937 | 3·941 | 3·944 |
| 50~54 | 6·9 ^α^ | 9·838 | 10·65 | 10·703 | 10·748 | **10·784 ^α^** | 10·822 | 10·852 | 10·879 |
| 54~59 | 21·2 ^α^ | 21·466 | 24·887 | 25·151 | 25·386 | **25·571 ^α^** | 25·784 | 25·954 | 26·107 |
| 60~64 | 50·4 ^α^ | 41·434 | 52·456 | 53·449 | 54·359 | **55·151 ^α^** | 55·968 | 56·681 | 57·343 |
| 65~69 | 106·4 ^α^ | 72·489 | 101·549 | 104·488 | 107·244 | **109·838 ^α^** | 112·281 | 114·591 | 116·779 |
| 70~74 | 213·6 ^α^ | 110·495 | 170·288 | 176·810 | 183·030 | **188·942 ^α^** | 194·685 | 200·165 | 205·439 |
| 75~79 | 293·5 ^α^ | 155·417 | 261·729 | 274·016 | 285·852 | **297·244 ^α^** | 308·351 | 319·084 | 329·411 |
| Overall Incidence | 45·9 ^β^ | 22·416 | 34·286 | 35·617 | 36·893 | **38·118 ^β^** | 39·299 | 40·439 | 41·541 |
| MAE | / | 32·26 | 10·29 | 8·02 | 6·03 | **5·34** | 6·33 | 7·27 | 8·17 |
| PCa, prostate cancer; WHO, World Health Organization.  RMSE, Root Mean Square Error; MAE, Mean Absolute Error  ^α^ Age-standardized rage. Data represent a weighted average of 919 sub-national cancer registries (2015–2017), applied to the 2022 population estimate^18^.  ^β^ Crude incidence rate.  ^γ^ The optimal parameter. | | | | | | | | | |

# Supplementary Table 3. Markov Model Simulated Survival

| **Survival Rate** | **Post-RP Local-Regional Disease** | **Post-RT Local-Regional Disease** | **mHSPC** | **nmCRPC** | **mCRPC** |
| --- | --- | --- | --- | --- | --- |
| **1-year (%)** | 99·20 | 44·84 | 79·52 | 60·67 | 57·32 |
| **3-year (%)** | 96·23 | 13·59 | 50·02 | 22·30 | 18·83 |
| **5-year (%)** | 92·22 | 5·86 | 31·34 | 8·17 | 6·19 |
| **10-year (%)** | 80·96 | 1·39 | 9·66 | 0·66 | 0·38 |
| RP, radical prostatectomy; RT, radiotherapy; mHSPC, metastatic hormone-sensitive prostate cancer; nmCRPC, nonmetastatic castration-resistant prostate cancer; mCRPC, metastatic castration-resistant prostate cancer | | | | | |

# Supplementary Table 4. Full Incremental Cost‐Effectiveness Results for All Simulated Prostate Cancer Screening Strategies

| **Strategy** | **Incremental Costs (USD)** | **Incremental QALYs** | **ICERs (USD)** | **CFR  (%)** | **Incidence (per 100,000)** | **Metastasis at Diagnosis  (per 100,000)** | **M/I Ratio (%)** |
| --- | --- | --- | --- | --- | --- | --- | --- |
| No screening (Baseline) | 0·00 | 0·00 | 0·00 | 6·14 | 99·46 | 38·84 | 39·05 |
| PSA cutoff = 4·0ng/mL; Age 45-74 Interval 1 year | 12047·09 | 2·65 | 4538·10 | 2·86 | 857·94 | 9·18 | 1·07 |
| PSA cutoff = 4·0ng/mL; Age 50-74 Interval 1 year | 10836·90 | 2·59 | 4180·19 | 2·87 | 818·97 | 9·5 | 1·16 |
| PSA cutoff = 4·0ng/mL; Age 55-74 Interval 1 year | 9172·91 | 2·45 | 3740·32 | 2·89 | 739·01 | 10·42 | 1·41 |
| PSA cutoff = 4·0ng/mL; Age 60-74 Interval 1 year | 7160·90 | 2·24 | 3195·88 | 2·93 | 633·16 | 12·41 | 1·96 |
| PSA cutoff = 4·0ng/mL; Age 45-74 Interval 2 years | 7922·46 | 2·4 | 3301·60 | 2·94 | 642·55 | 12·08 | 1·88 |
| PSA cutoff = 4·0ng/mL; Age 50-74 Interval 2 years | 7300·70 | 2·36 | 3099·14 | 2·95 | 628·06 | 12·31 | 1·96 |
| PSA cutoff = 4·0ng/mL; Age 55-74 Interval 2 years | 5924·61 | 2·2 | 2688·15 | 2·97 | 582·97 | 13·35 | 2·29 |
| PSA cutoff = 4·0ng/mL; Age 60-74 Interval 2 years | 4747·92 | 2·04 | 2321·85 | 3·01 | 530·71 | 14·86 | 2·8 |
| PSA cutoff = 4·0ng/mL; Age 45-74 Interval 3 years | 6142·42 | 2·21 | 2777·74 | 2·99 | 580·67 | 13·82 | 2·38 |
| PSA cutoff = 4·0ng/mL; Age 50-74 Interval 3 years | 5473·27 | 2·16 | 2534·54 | 3 | 568·95 | 14·11 | 2·48 |
| PSA cutoff = 4·0ng/mL; Age 55-74 Interval 3 years | 4370·41 | 2·02 | 2164·71 | 3·03 | 531·47 | 15·2 | 2·86 |
| PSA cutoff = 4·0ng/mL; Age 60-74 Interval 3 years | 3663·46 | 1·91 | 1916·22 | 3·06 | 500·31 | 16·31 | 3·26 |
| PSA cutoff = 4·0ng/mL; Age 45-74 Interval 5 years | 4235·62 | 1·94 | 2186·35 | 3·10 | 476·52 | 17·25 | 3·62 |
| PSA cutoff = 4·0ng/mL; Age 50-74 Interval 5 years | 3889·86 | 1·91 | 2033·35 | 3·11 | 472·28 | 17·38 | 3·68 |
| PSA cutoff = 4·0ng/mL; Age 55-74 Interval 5 years | 3328·34 | 1·84 | 1805·01 | 3·12 | 458·46 | 17·88 | 3·9 |
| PSA cutoff = 4·0ng/mL; Age 60-74 Interval 5 years | 2494·55 | 1·71 | 1458·85 | 3·16 | 430·49 | 19·2 | 4·46 |
| Age-specific PSA cutoffs; Age 45-74 Interval 1 year | 14018·50 | 2·76 | 5083·31 | 2·86 | 819·47 | 9·26 | 1·13 |
| Age-specific PSA cutoffs; Age 50-74 Interval 1 year | 11753·50 | 2·67 | 4408·12 | 2·87 | 775·2 | 9·69 | 1·25 |
| Age-specific PSA cutoffs; Age 55-74 Interval 1 year | 9552·18 | 2·50 | 3817·36 | 2·89 | 701·97 | 10·67 | 1·52 |
| Age-specific PSA cutoffs; Age 60-74 Interval 1 year | 6917·96 | 2·25 | 3072·12 | 2·94 | 593·98 | 12·83 | 2·16 |
| Age-specific PSA cutoffs; Age 45-74 Interval 2 years | 9277·70 | 2·51 | 3693·24 | 2·94 | 608·96 | 12·24 | 2·01 |
| Age-specific PSA cutoffs; Age 50-74 Interval 2 years | 8099·10 | 2·44 | 3321·03 | 2·95 | 590·61 | 12·58 | 2·13 |
| Age-specific PSA cutoffs; Age 55-74 Interval 2 years | 6265·96 | 2·25 | 2783·53 | 2·98 | 544·84 | 13·73 | 2·52 |
| Age-specific PSA cutoffs; Age 60-74 Interval 2 years | 4719·44 | 2·05 | 2298·17 | 3·03 | 488·29 | 15·43 | 3·16 |
| Age-specific PSA cutoffs; Age 45-74 Interval 3 years | 7410·04 | 2·33 | 3180·15 | 2·98 | 556·85 | 13·81 | 2·48 |
| Age-specific PSA cutoffs; Age 50-74 Interval 3 years | 6136·21 | 2·24 | 2739·21 | 3 | 538·11 | 14·26 | 2·65 |
| Age-specific PSA cutoffs; Age 55-74 Interval 3 years | 4676·60 | 2·06 | 2266·08 | 3·04 | 498·39 | 15·5 | 3·11 |
| Age-specific PSA cutoffs; Age 60-74 Interval 3 years | 3754·19 | 1·93 | 1945·88 | 3·08 | 463·26 | 16·77 | 3·62 |
| Age-specific PSA cutoffs; Age 45-74 Interval 5 years | 5166·71 | 2·03 | 2551·04 | 3·11 | 440·15 | 17·65 | 4·01 |
| Age-specific PSA cutoffs; Age 50-74 Interval 5 years | 4458·10 | 1·98 | 2255·51 | 3·12 | 433·17 | 17·89 | 4·13 |
| Age-specific PSA cutoffs; Age 55-74 Interval 5 years | 3671·30 | 1·88 | 1950·13 | 3·15 | 418·1 | 18·48 | 4·42 |
| Age-specific PSA cutoffs; Age 60-74 Interval 5 years | 2544·55 | 1·71 | 1491·49 | 3·21 | 385·74 | 20·02 | 5·19 |
| PRS+FH; PSA cutoff = 4·0ng/mL; High-risk group Age 45-74 Interval 1 year; Normal-risk group no screening | 8348·86 | 2·30 | 3637·39 | 5·05 | 90·16 | 23·66 | 26·24 |
| PRS+FH; PSA cutoff = 4·0ng/mL; High-risk group Age 45-74 Interval 1 year; Normal-risk group Age 50-74 Interval 1 year | 11583·38 | 2·63 | 4402·04 | 2·87 | 822·28 | 9·32 | 1·13 |
| PRS+FH; PSA cutoff = 4·0ng/mL; High-risk group Age 45-74 Interval 1 year; Normal-risk group Age 50-74 Interval 2 years | 9386·41 | 2·53 | 3714·40 | 2·91 | 642·69 | 10·68 | 1·66 |
| PRS+FH; PSA cutoff = 4·0ng/mL; High-risk group Age 45-74 Interval 1 year; Normal-risk group Age 55-74 Interval 1 year | 10568·91 | 2·56 | 4120·91 | 2·88 | 754·46 | 9·76 | 1·29 |
| PRS+FH; PSA cutoff = 4·0ng/mL; High-risk group Age 45-74 Interval 1 year; Normal-risk group Age 55-74 Interval 2 years | 8658·57 | 2·46 | 3517·05 | 2·93 | 593·67 | 11·17 | 1·88 |
| PRS+FH; PSA cutoff = 4·0ng/mL; High-risk group Age 45-74 Interval 1 year; Normal-risk group Age 60-74 Interval 1 year | 9408·99 | 2·47 | 3811·27 | 2·91 | 647·30 | 10·71 | 1·65 |
| PRS+FH; PSA cutoff = 4·0ng/mL; High-risk group Age 45-74 Interval 1 year; Normal-risk group Age 60-74 Interval 2 years | 8093·77 | 2·40 | 3375·22 | 2·96 | 535·42 | 11·90 | 2·22 |
| PRS+FH; Age-specific PSA cutoffs; High-risk group Age 45-74 Interval 1 year; Normal-risk group no screening | 9562·82 | 2·37 | 4033·97 | 5·05 | 90·22 | 23·69 | 26·26 |
| PRS+FH; Age-specific PSA cutoffs; High-risk group Age 45-74 Interval 1 year; Normal-risk group Age 50-74 Interval 1 year | 12879·51 | 2·72 | 4734·38 | 2·87 | 780·35 | 9·44 | 1·21 |
| PRS+FH; Age-specific PSA cutoffs; High-risk group Age 45-74 Interval 1 year; Normal-risk group Age 50-74 Interval 2 years | 10626·80 | 2·62 | 4051·79 | 2·92 | 602·87 | 10·84 | 1·80 |
| PRS+FH; Age-specific PSA cutoffs; High-risk group Age 45-74 Interval 1 year; Normal-risk group Age 55-74 Interval 1 year | 11530·90 | 2·64 | 4363·06 | 2·88 | 712·76 | 9·91 | 1·39 |
| PRS+FH; Age-specific PSA cutoffs; High-risk group Age 45-74 Interval 1 year; Normal-risk group Age 55-74 Interval 2 years | 9661·66 | 2·54 | 3798·81 | 2·94 | 553·18 | 11·39 | 2·06 |
| PRS+FH; Age-specific PSA cutoffs; High-risk group Age 45-74 Interval 1 year; Normal-risk group Age 60-74 Interval 1 year | 10022·96 | 2·53 | 3959·59 | 2·91 | 604·32 | 10·95 | 1·81 |
| PRS+FH; Age-specific PSA cutoffs; High-risk group Age 45-74 Interval 1 year; Normal-risk group Age 60-74 Interval 2 years | 8938·68 | 2·47 | 3623·94 | 2·97 | 492·43 | 12·21 | 2·48 |
| WGS+PRS+FH; PSA cutoff = 4·0ng/mL; High-risk group Age 45-74 Interval 1 year; Normal-risk group no screening | 10166·55 | 2·32 | 4378·03 | 4·99 | 89·82 | 23·03 | 25·64 |
| WGS+PRS+FH; PSA cutoff = 4·0ng/mL; High-risk group Age 45-74 Interval 1 year; Normal-risk group Age 50-74 Interval 1 year | 14571·99 | 2·63 | 5534·56 | 2·87 | 822·59 | 9·31 | 1·13 |
| WGS+PRS+FH; PSA cutoff = 4·0ng/mL; High-risk group Age 45-74 Interval 1 year; Normal-risk group Age 50-74 Interval 2 years | 12620·27 | 2·53 | 4981·27 | 2·91 | 644·14 | 10·61 | 1·65 |
| WGS+PRS+FH; PSA cutoff = 4·0ng/mL; High-risk group Age 45-74 Interval 1 year; Normal-risk group Age 55-74 Interval 1 year | 13619·69 | 2·57 | 5301·11 | 2·88 | 755·08 | 9·73 | 1·29 |
| WGS+PRS+FH; PSA cutoff = 4·0ng/mL; High-risk group Age 45-74 Interval 1 year; Normal-risk group Age 55-74 Interval 2 years | 11959·72 | 2·47 | 4838·95 | 2·93 | 594·93 | 11·08 | 1·86 |
| WGS+PRS+FH; PSA cutoff = 4·0ng/mL; High-risk group Age 45-74 Interval 1 year; Normal-risk group Age 60-74 Interval 1 year | 12570·66 | 2·48 | 5073·52 | 2·91 | 648·29 | 10·64 | 1·64 |
| WGS+PRS+FH; PSA cutoff = 4·0ng/mL; High-risk group Age 45-74 Interval 1 year; Normal-risk group Age 60-74 Interval 2 years | 11479·15 | 2·41 | 4761·32 | 2·96 | 536·58 | 11·78 | 2·19 |
| WGS+PRS+FH; Age-specific PSA cutoffs; High-risk group Age 45-74 Interval 1 year; Normal-risk group no screening | 11458·73 | 2·40 | 4775·95 | 4·99 | 89·88 | 23·06 | 25·66 |
| WGS+PRS+FH; Age-specific PSA cutoffs; High-risk group Age 45-74 Interval 1 year; Normal-risk group Age 50-74 Interval 1 year | 16008·99 | 2·72 | 5880·06 | 2·87 | 780·94 | 9·44 | 1·21 |
| WGS+PRS+FH; Age-specific PSA cutoffs; High-risk group Age 45-74 Interval 1 year; Normal-risk group Age 50-74 Interval 2 year | 14026·73 | 2·63 | 5334·04 | 2·92 | 604·50 | 10·77 | 1·78 |
| WGS+PRS+FH; Age-specific PSA cutoffs; High-risk group Age 45-74 Interval 1 year; Normal-risk group Age 55-74 Interval 1 year | 13147·27 | 2·65 | 4964·07 | 2·88 | 717·03 | 9·88 | 1·38 |
| WGS+PRS+FH; Age-specific PSA cutoffs; High-risk group Age 45-74 Interval 1 year; Normal-risk group Age 55-74 Interval 2 years | 13367·91 | 2·55 | 5233·80 | 2·94 | 554·63 | 11·29 | 2·04 |
| WGS+PRS+FH; Age-specific PSA cutoffs; High-risk group Age 45-74 Interval 1 year; Normal-risk group Age 60-74 Interval 1 year | 12533·62 | 2·54 | 4930·22 | 2·91 | 605·56 | 10·87 | 1·80 |
| WGS+PRS+FH; Age-specific PSA cutoffs; High-risk group Age 45-74 Interval 1 year; Normal-risk group Age 60-74 Interval 2 years | 13303·73 | 2·48 | 5361·45 | 2·97 | 493·50 | 12·08 | 2·45 |
| PSA, Prostate‐Specific Antigen; PRS, Polygenic Risk Score; WGS, Whole Genome Sequencing.  Incremental Costs and Incremental QALYs are calculated compared to a no‐screening situation; ICER (Incremental Cost‐Effectiveness Ratio) = (Incremental Costs)/(Incremental QALYs); CFR, case fatality rate; Metastasis at Diagnosis, number of patient diagnosed with metastasis when initially detected (per 100000); Metastasis/Incidence Ratio (%), metastasis number when initially detected/annual incidence.  High‐risk group and Low‐risk group definitions reflect different starting ages or intervals triggered by PRS or WGS findings. To be specific, individuals with RPMs (rare pathogenic mutation) of three genes: BRCA2, HOXB13, and CHEK2, tested by WGS, or with family history, or divided by PRS into the top quartile are grouped as high-risk. Colour shading indicates different strategies: Normal PSA screening (cutoff=4 or age-specific cutoffs), one-time PRS test + PSA screening or WGS + PSA screening. | | | | | | | |

# Supplementary Table 5. Robustness Analysis: The Influence of High-Risk Group Proportion on Cost‐Effectiveness Results

| **Strategy** | **Increment QALYs (FH = 8·2%)** | **ICERs**  **(USD, FH = 8·2%)** | **Increment QALYs (FH = 0%)** | **ICERs  (USD, FH = 0%)** |
| --- | --- | --- | --- | --- |
| **PRS** ^ß^ **Test + Age-specific PSA cutoffs** ^α^**,  High-risk Group**ᵞ**: Age 45-74, Interval: 1 year;  Low-risk Group: 50-74, Interval: 1 year** | 2·72 | 4734·38 | 2·71 | 4679·97 |
| **PRS Test + Age-specific PSA cutoffs,**  **High-risk Group: Age 45-74, Interval: 1 year;**  **Low-risk Group: 50-74, Interval: 2 years** | 2·62 | 4051·79 | 2·59 | 3907·65 |
| **PRS Test + Age-specific PSA cutoffs,**  **High-risk Group: Age 45-74, Interval: 1 year;**  **Low-risk Group: 55-74, Interval: 1 year** | 2·64 | 4363·06 | 2·62 | 4259·03 |
| **PRS Test + Age-specific PSA cutoffs,**  **High-risk Group: Age 45-74, Interval: 1 year;**  **Low-risk Group: 55-74, Interval: 2 years** | 2·54 | 3798·81 | 2·49 | 3606·10 |
| **PRS Test + Age-specific PSA cutoffs,**  **High-risk Group: Age 45-74, Interval: 1 year;**  **Low-risk Group: 60-74, Interval: 1 year** | 2·53 | 3959·59 | 2·48 | 3790·36 |
| **PRS Test + Age-specific PSA cutoffs,**  **High-risk Group: Age 45-74, Interval: 1 year;**  **Low-risk Group: 60-74, Interval: 2 years** | 2·47 | 3623·94 | 2·40 | 3388·65 |
| **PRS Test + PSA cutoff = 4,**  **High-risk Groupᵞ: Age 45-74, Interval: 1 year;**  **Low-risk Group: 50-74, Interval: 1 year** | 2·63 | 4402·04 | 2·62 | 4373·55 |
| **PRS Test + PSA cutoff = 4·0ng/mL,**  **High-risk Group: Age 45-74, Interval: 1 year;**  **Low-risk Group: 50-74, Interval: 2 years** | 2·53 | 3714·40 | 2·50 | 3597·11 |
| **PRS Test + PSA cutoff = 4·0ng/mL,**  **High-risk Group: Age 45-74, Interval: 1 year;**  **Low-risk Group: 55-74, Interval: 1 year** | 2·56 | 4120·91 | 2·54 | 4055·98 |
| **PRS Test + PSA cutoff = 4·0ng/mL,**  **High-risk Group: Age 45-74, Interval: 1 year;**  **Low-risk Group: 55-74, Interval: 2 years** | 2·46 | 3517·05 | 2·42 | 3362·62 |
| **PRS Test + PSA cutoff = 4·0ng/mL,**  **High-risk Group: Age 45-74, Interval: 1 year;**  **Low-risk Group: 60-74, Interval: 1 year** | 2·47 | 3811·27 | 2·43 | 3699·62 |
| **PRS Test + PSA cutoff = 4·0ng/mL,**  **High-risk Group: Age 45-74, Interval: 1 year;**  **Low-risk Group: 60-74, Interval: 2 years** | 2·40 | 3375·22 | 2·34 | 3188·24 |
| QALY, quality-adjusted life year; ICER, incremental cost-effectiveness ratio; PSA, prostate-specific antigen;  PRS, polygenic risk score; PCa, prostate cancer.  ^α^ Age-specific cutoff denotes the cutoff is 2·0 ng/mL 3·0 ng/mL 4·0 ng/mL and 7·0 ng/mL for age categories <50 years 50–59 years 60–69 years and ≥70 years  ^ß^ PRS was divided into three groups: bottom quartile for low PRS, middle two quartiles for average PRS, and top quartile for high PRS.  ᵞ High-risk group includes males either with family history or with top quartile for high PRS. | | | | |

# Supplementary Table 6. Numbers of Extra PSA Tests, Biopsies, Treatments per Life Saved with Different Screening Strategies compared with no-Screening Scenario Projected by the Models

| **Screening Strategy** | **PSA test per life gained** | **Biopsy per life gained** | **Treatment per life gained** | **Incremental QALYs** |
| --- | --- | --- | --- | --- |
| PSA cutoff = 4; Age 45-74 Interval 1 year | 907·89 | 162·80 | 5·30 | 2·65 |
| PSA cutoff = 4; Age 50-74 Interval 1 year | 749·17 | 144·58 | 5·25 | 2·59 |
| PSA cutoff = 4; Age 55-74 Interval 1 year | 585·26 | 122·36 | 5·13 | 2·45 |
| PSA cutoff = 4; Age 60-74 Interval 1 year | 422·96 | 96·46 | 4·85 | 2·24 |
| PSA cutoff = 4; Age 45-74 Interval 2 years | 457·43 | 84·38 | 4·59 | 2·40 |
| PSA cutoff = 4; Age 50-74 Interval 2 years | 392·36 | 76·91 | 4·56 | 2·36 |
| PSA cutoff = 4; Age 55-74 Interval 2 years | 291·57 | 63·22 | 4·42 | 2·20 |
| PSA cutoff = 4; Age 60-74 Interval 2 years | 224·85 | 52·52 | 4·22 | 2·04 |
| PSA cutoff = 4; Age 45-74 Interval 3 years | 338·29 | 63·46 | 4·42 | 2·21 |
| PSA cutoff = 4; Age 50-74 Interval 3 years | 272·29 | 56·00 | 4·38 | 2·16 |
| PSA cutoff = 4; Age 55-74 Interval 3 years | 203·81 | 46·53 | 4·24 | 2·02 |
| PSA cutoff = 4; Age 60-74 Interval 3 years | 169·82 | 41·06 | 4·10 | 1·91 |
| PSA cutoff = 4; Age 45-74 Interval 5 years | 222·72 | 42·95 | 3·89 | 1·94 |
| PSA cutoff = 4; Age 50-74 Interval 5 years | 188·92 | 39·33 | 3·87 | 1·91 |
| PSA cutoff = 4; Age 55-74 Interval 5 years | 153·91 | 34·88 | 3·81 | 1·84 |
| PSA cutoff = 4; Age 60-74 Interval 5 years | 118·44 | 29·52 | 3·65 | 1·71 |
| Age-specific PSA cutoffs; Age 45-74 Interval 1 year^α^ | 905·72 | 173·48 | 5·04 | 2·76 |
| Age-specific PSA cutoffs; Age 50-74 Interval 1 year^α^ | 748·86 | 146·36 | 4·98 | 2·67 |
| Age-specific PSA cutoffs; Age 55-74 Interval 1 year^α^ | 586·10 | 120·33 | 4·85 | 2·50 |
| Age-specific PSA cutoffs; Age 60-74 Interval 1 year^α^ | 425·78 | 90·52 | 4·54 | 2·25 |
| Age-specific PSA cutoffs; Age 45-74 Interval 2 years^α^ | 456·51 | 88·67 | 4·31 | 2·51 |
| Age-specific PSA cutoffs; Age 50-74 Interval 2 years^α^ | 392·42 | 77·57 | 4·26 | 2·44 |
| Age-specific PSA cutoffs; Age 55-74 Interval 2 years^α^ | 292·56 | 61·55 | 4·11 | 2·25 |
| Age-specific PSA cutoffs; Age 60-74 Interval 2 years^α^ | 226·95 | 49·24 | 3·87 | 2·05 |
| Age-specific PSA cutoffs; Age 45-74 Interval 3 years^α^ | 337·29 | 67·33 | 4·17 | 2·33 |
| Age-specific PSA cutoffs; Age 50-74 Interval 3 years^α^ | 272·29 | 56·20 | 4·10 | 2·24 |
| Age-specific PSA cutoffs; Age 55-74 Interval 3 years^α^ | 204·58 | 45·14 | 3·93 | 2·06 |
| Age-specific PSA cutoffs; Age 60-74 Interval 3 years^α^ | 171·23 | 38·84 | 3·77 | 1·93 |
| Age-specific PSA cutoffs; Age 45-74 Interval 5 years^α^ | 223·69 | 44·15 | 3·54 | 2·03 |
| Age-specific PSA cutoffs; Age 50-74 Interval 5 years^α^ | 190·22 | 38·62 | 3·49 | 1·98 |
| Age-specific PSA cutoffs; Age 55-74 Interval 5 years^α^ | 155·48 | 33·35 | 3·41 | 1·88 |
| Age-specific PSA cutoffs; Age 60-74 Interval 5 years^α^ | 120·64 | 27·13 | 3·23 | 1·71 |
| PRS+FH; Age-specific PSA cutoffs; High-risk group Age 45-74 Interval 1 year; Normal-risk group Age 50-74 Interval 1 year | 801·88 | 155·53 | 4·88 | 2·72 |
| PRS+FH; Age-specific PSA cutoffs; High-risk group Age 45-74 Interval 1 year; Normal-risk group Age 50-74 Interval 2 years | 547·86 | 106·52 | 4·51 | 2·62 |
| PRS+FH; Age-specific PSA cutoffs; High-risk group Age 45-74 Interval 1 year; Normal-risk group Age 55-74 Interval 1 year | 687·74 | 137·28 | 4·82 | 2·64 |
| PRS+FH; Age-specific PSA cutoffs; High-risk group Age 45-74 Interval 1 year; Normal-risk group Age 55-74 Interval 2 years | 477·90 | 95·32 | 4·43 | 2·54 |
| Extra tests, extra biopsies and extra treatments are defined as those managements performed in screening scenarios which would not be performed in no-screening scenario.  QALY, quality-adjusted life year; PSA, prostate specific antigen; PCa, prostate cancer.  ^α^ Age-specific cutoff denotes the cutoff is 2·0 ng/mL 3·0 ng/mL 4·0 ng/mL and 7·0 ng/mL for age categories <50 years 50–59 years 60–69 years and ≥70 years | | | | |

# Reference

1 Gulati R, Inoue L, Katcher J, Hazelton W, Etzioni R. Calibrating disease progression models using population data: a critical precursor to policy development in cancer control. *Biostatistics* 2010; **11**: 707–19.

2 Keeney E, Sanghera S, Martin RM, *et al.* Cost-Effectiveness Analysis of Prostate Cancer Screening in the UK: A Decision Model Analysis Based on the CAP Trial. *Pharmacoeconomics* 2022; **40**: 1207–20.

3 Heijnsdijk EAM, Gulati R, Lange JM, Tsodikov A, Roberts R, Etzioni R. Evaluation of Prostate Cancer Screening Strategies in a Low-Resource, High-risk Population in the Bahamas. *JAMA Health Forum* 2022; **3**: e221116.

4 Karlsson A, Jauhiainen A, Gulati R, *et al.* A natural history model for planning prostate cancer testing: Calibration and validation using Swedish registry data. *PLoS One* 2019; **14**: e0211918.

5 Caruana M, Gulati R, Etzioni R, *et al.* Benefits and harms of prostate specific antigen testing according to Australian guidelines. *Int J Cancer* 2024; **154**: 648–58.

6 Pashayan N, Duffy SW, Pharoah P, *et al.* Mean sojourn time, overdiagnosis, and reduction in advanced stage prostate cancer due to screening with PSA: implications of sojourn time on screening. *Br J Cancer* 2009; **100**: 1198–204.

7 Hutchinson F. Fred Hutchinson Cancer Research Centre (PSAPC). .

8 He J, Chen W, Li N, *et al.* China Guideline for the Screening and Early Detection of Prostate Cancer (2022, Beijing). *China Oncology* 2022; **31**: 1–30.

9 Liu J. Prostate cancer treatment – China’s perspective. *Cancer Letters* 2022; **1**: 215827.

10 Woo S, Suh CH, Kim SY, Cho JY, Kim SH. Diagnostic Performance of Prostate Imaging Reporting and Data System Version 2 for Detection of Prostate Cancer: A Systematic Review and Diagnostic Meta-analysis. *European Urology* 2017; **72**: 177–88.

11 Padhani AR, Weinreb J, Rosenkrantz AB, Villeirs G, Turkbey B, Barentsz J. Prostate Imaging-Reporting and Data System Steering Committee: PI-RADS v2 Status Update and Future Directions. *European Urology* 2019; **75**: 385–96.

12 Tonttila PP, Lantto J, Pääkkö E, *et al.* Prebiopsy Multiparametric Magnetic Resonance Imaging for Prostate Cancer Diagnosis in Biopsy-naive Men with Suspected Prostate Cancer Based on Elevated Prostate-specific Antigen Values: Results from a Randomized Prospective Blinded Controlled Trial. *European Urology* 2016; **69**: 419–25.

13 Drost F-JH, Osses D, Nieboer D, *et al.* Prostate Magnetic Resonance Imaging, with or Without Magnetic Resonance Imaging-targeted Biopsy, and Systematic Biopsy for Detecting Prostate Cancer: A Cochrane Systematic Review and Meta-analysis. *Eur Urol* 2020; **77**: 78–94.

14 Schaeffer EM, Srinivas S, Adra N, *et al.* NCCN Guidelines Version 1.2025 Prostate Cancer. *Prostate Cancer* 2024.

15 Zhongguo Linchuang Zhongliu Xuehui(CSCO) Qianliexian Ai Zhenliao Zhinan 2023. Beijing, China: People’s Medical Publishing House, 2023.

16 China Population Census Book-2020. https://www.stats.gov.cn/sj/pcsj/rkpc/7rp/indexch.htm (accessed Jan 18, 2025).

17 Shi Z, Platz EA, Wei J, *et al.* Performance of Three Inherited Risk Measures for Predicting Prostate Cancer Incidence and Mortality: A Population-based Prospective Analysis. *Eur Urol* 2021; **79**: 419–26.

18 Cancer Over Time. https://gco.iarc.fr/overtime (accessed Jan 18, 2025).
